# Supplementary material for: Accelerating clinical development of a live attenuated vaccine against Salmonella Paratyphi A (VASP): study protocol for an observer-participant-blind randomised control trial of a novel oral vaccine using a human challenge model of Salmonella Paratyphi A infection in healthy adult volunteers
Source: BMJ Open. 2023 May 23;13(5):e068966. doi: 10.1136/bmjopen-2022-068966 (PMC10230971; doi:10.1136/bmjopen-2022-068966)
Supplement: Supplementary data [file bmjopen-2022-068966supp001.pdf]

Supplementary Material 1: Inclusion and Exclusion Criteria

Inclusion Criteria

Participants must satisfy all the following criteria to be considered eligible for the study:

- Willing and able to give informed consent for participation in the study.
- Aged between 18 and 55 years inclusive at time of vaccination.
- In good health as determined by medical history, physical examination and clinical judgment of the study team.
- Willing to be available in Oxford for all required appointments
- Agree (in the study team's opinion) to comply with all study requirements, including capacity to adhere to good personal hygiene and infection control precautions.
- Agree to allow study staff to contact his or her GP to access the participant's vaccination records, medical history and have their opinion solicited as to the participant's appropriateness for inclusion.
- Agree to allow study staff to access NHS health records as required for study purposes.
- Agree to allow his or her GP (and/or Consultant if appropriate), to be notified of participation in the study.
- Agree to allow UKHSA to be informed of their participation in the study.
- Agree to give his or her close household contacts written information informing them of the participants' involvement in the study and offering them voluntary screening for *S.* Paratyphi A carriage.
- Agree to have 24-hour contact with study staff during the four weeks post challenge and are able to ensure that they are contactable by mobile phone for the duration of the vaccination and challenge period until antibiotic completion.
- Have internet access to allow completion of the e-diary and real-time safety monitoring.
- Agree to avoid antipyretic/anti-inflammatory treatment from challenge until advised by a study doctor or until 14 days after challenge.
- Agree to refrain from donating blood for the duration of the study.
- Agree to provide their National Insurance/Passport number for the purposes of TOPS registration and for payment of reimbursement expenses.
- Participants must have received at least one dose of a SARS-CoV-2 vaccine that has been approved for use by the MHRA (or other national regulatory authority) $\geq$ four weeks prior to enrollment.

- Agree to not receive other vaccinations (eg Covid-19 vaccines) during the 7 days before and after study vaccination and during the 7 days before or 21 days post-challenge.

1.1. Exclusion Criteria

The participant will not be enrolled if any of the following apply:

- History of significant organ/system disease that could interfere with trial conduct or completion. Including, for example, but not restricted to:
    - Cardiovascular disease including a diagnosis of hypertension
    - Respiratory disease
    - Haematological disease[1]
    - Endocrine disorders
    - Renal or bladder disease, including history of renal calculi
    - Biliary tract disease, including biliary colic, asymptomatic gallstones or previous cholecystectomy
    - Gastro-intestinal disease including requirement for antacids, H_2-receptor antagonists, proton pump inhibitors or laxatives
    - Neurological disease
    - Metabolic disease
    - Autoimmune disease
    - Psychiatric illness requiring hospitalisation
    - Known or suspected drug misuse
    - Known or suspected alcohol misuse (alcohol misuse defined as an intake exceeding 42 units per week)
    - Infectious disease
- Have any known or suspected impairment of immune function, alteration of immune function, or prior immune exposure that may alter immune function to paratyphoid resulting from, for example:
    - Congenital or acquired immunodeficiency, including IgA deficiency
    - Human Immunodeficiency Virus infection or symptoms/signs suggestive of an HIV-associated condition

[1] This includes anaemia. The acceptable lower limits for [haemoglobin] are 125 g/L for female participants and 135 g/L for male participants (Guidelines for the Blood Transfusion Services in the UK, 8th edition, 2018 <https://www.transfusionguidelines.org/red-book> Accessed 5th December 2018).

    - Receipt of immunosuppressive therapy such as anti-cancer chemotherapy or radiation therapy within the preceding 12 months or long-term systemic corticosteroid therapy.
    - Receipt of immunoglobulin or any blood product transfusion within 3 months of study start.
    - History of cancer (except squamous cell or basal cell carcinoma of the skin and cervical carcinoma in situ).
- HLA-B27 positive.
- Moderate or severe depression or anxiety as classified by the Hospital Anxiety and Depression Score at screening or challenge that is deemed clinically significant by the study doctors[2].
- Weight less than 50 kg.
- Presence of implants or prosthetic material.
- Anyone taking long-term medication (e.g. analgesia, anti-inflammatories or antibiotics) that may affect symptom reporting or interpretation of the study results.
- Contraindication to fluoroquinolones, macrolide antibiotics, co-trimoxazole or ceftriaxone.
- Family history of aneurysmal disease
- Female participants who are pregnant, lactating or who are unwilling to ensure that they or their partner use effective contraception[3] 30 days prior to vaccination and continue to do so until three negative stool samples have been obtained after completion of antibiotic treatment.
- Full-time, part-time or voluntary occupations involving:
    - Clinical or social work with direct contact with young children (defined as those attending pre-school groups or nursery or aged under 2 years), or
    - Clinical or social work with direct contact with highly susceptible patients or persons in whom typhoid infection would have particularly serious consequences
    - Commercial food handling (involving preparing or serving unwrapped foods not subjected to further heating)

  (unless willing to avoid work from vaccination until demonstrated not to be infected with *S.* Paratyphi A after challenge by clearance samples in accordance with guidance from UKHSA and willing to allow study staff to inform their employer).

---

[2] If elevated scores are due to temporary significant life events, the questionnaire may be repeated after resolution of the event with a view to inclusion if normal.

[3] As defined by CTFG Recommendations related to contraception and pregnancy testing in clinical trials, current document: https://www.hma.eu/fileadmin/dateien/Human_Medicines/01-About_HMA/Working_Groups/CTFG/2014_09_HMA_CTFG_Contraception.pdf [accessed 23rd July 2019]

- Close household contact with:
  - Young children (defined as those attending pre-school groups, nursery or those aged less than 2 years)
  - Individuals who are immunocompromised (including pregnancy).
- Scheduled elective surgery or other procedures requiring general anaesthesia during the study period.
- Participants who have participated in another research study involving an investigational product that might affect risk of paratyphoid infection or compromise the integrity of the study within the 30 days prior to enrolment (e.g. significant volumes of blood already taken in previous study)[4].
- Detection of any abnormal results from screening investigations (at the clinical discretion of the study team).
- Inability to comply with any of the study requirements (at the discretion of the study staff and the participant's General Practitioner).
- Any other social, psychological or health issues which, in the opinion of the study staff, may
  - put the participant or their contacts at risk because of participation in the study,
  - adversely affect the interpretation of the primary endpoint data,
  - Impair the participant's ability to participate in the study.
- Have any history of allergy to vaccine/placebo components
- Having been resident in an enteric fever endemic country for 6 months or more.
- Have previously been diagnosed with laboratory-confirmed typhoid or paratyphoid infection or been given a diagnosis compatible with enteric fever.
- Have participated in previous typhoid or paratyphoid challenge studies (with ingestion of challenge agent).
- Have received any oral typhoid vaccination (e.g. Ty21a or M01ZH09) at any time.
- Have a prolonged corrected QT interval (>450 milliseconds) on ECG screening.
- Significant blood donation or planned blood donation prior to enrolment.

[4] As assessed by both participant questioning and registration with The Over Volunteering Prevention System (TOPS) database.
