# Supplementary material for: Accelerating clinical development of a live attenuated vaccine against Salmonella Paratyphi A (VASP): study protocol for an observer-participant-blind randomised control trial of a novel oral vaccine using a human challenge model of Salmonella Paratyphi A infection in healthy adult volunteers
Source: BMJ Open. 2023 May 23;13(5):e068966. doi: 10.1136/bmjopen-2022-068966 (PMC10230971; doi:10.1136/bmjopen-2022-068966)
Supplement: Supplementary data [file bmjopen-2022-068966supp002.pdf]

Supplementary Material 2: Temporary Exclusion Criteria

Temporary Exclusion at Vaccination

Participants will be temporarily excluded from receiving vaccination if presenting at a vaccination visit with the following:

- Acute gastrointestinal illness within 24-hours prior to vaccination.
- Significant infection within the previous 7 days.
- Participant has experienced fever (>37.5□C) or subjective febrile symptoms within the previous 3 days (even with a negative COVID-19 test).
- Symptoms of COVID-19, or confirmed infection (according to government guidelines) within 14 days prior to vaccination visit; as per guidance in Section 9.11
- Validated positive SARS-CoV-2 test (NAAT or antigen) within 2 weeks prior to vaccination visit
- History of any antibiotic therapy during the previous 5 days.
- Any systemic corticosteroid (or equivalent) treatment in the previous 14 days, or for more than seven consecutive days within the past 3 months).
- Receipt of another enteric live vaccine within 4 weeks prior to vaccination or an injected live or killed vaccine within 7 days prior to vaccination.
- Plan to receive any vaccine other than the study vaccine within 7 days following vaccination.
- Therapy with antacids, proton pump inhibitors or H_2-receptor antagonists within 24 hours prior to vaccination.
- Unavailable for post-vaccination visits, second vaccination visit, and challenge visit as outlined in study procedures table (see section 7.4 table 1b).

If this is the first vaccine to be received and the temporary exclusion does not result in the participant becoming ineligible, then this vaccine visit can be rescheduled.

If this is the second vaccine to be received and the temporary exclusion does not result in the participant becoming ineligible, then this should be discussed with a study doctor as to whether they can be rescheduled for their second vaccination (see table 2, section 7.5 for visit windows) or withdrawn.

Temporary Exclusion at Challenge

Participants will be temporarily excluded from challenge if presenting at the challenge visit with the following. Participants can be challenged up to 28 days after their original challenge date (see table 2, section 7.5)

- Significant acute or acute-on-chronic infection within the previous 7 days or have experienced fever (>37.5°C) or subjective febrile symptoms within the previous 3 days (even with a negative COVID-19 test).
- Symptoms of COVID-19, or confirmed infection (according to government guidelines) within 14 days prior to challenge visit; as per guidance in Section 9.11
- Validated positive SARS-CoV-2 test (NAAT or antigen) within 2 weeks prior to challenge visit
- History of any antibiotic therapy during the previous 5 days.
- Any systemic corticosteroid (or equivalent) treatment in the previous 14 days, or for more than seven consecutive days within the past 3 months.
- Therapy with antacids, proton pump inhibitors or H_2-receptor antagonists within 24 hours prior to challenge.
- Participant has not received two doses of the study vaccine/placebo.
- Anaemia felt to be clinically significant by the study team.
- Receipt of a COVID-19 vaccine in preceding 7 days
- Plan to receive any vaccine within 21 days following challenge.
