# Supplementary material for: Accelerating clinical development of a live attenuated vaccine against Salmonella Paratyphi A (VASP): study protocol for an observer-participant-blind randomised control trial of a novel oral vaccine using a human challenge model of Salmonella Paratyphi A infection in healthy adult volunteers
Source: BMJ Open. 2023 May 23;13(5):e068966. doi: 10.1136/bmjopen-2022-068966 (PMC10230971; doi:10.1136/bmjopen-2022-068966)
Supplement: Supplementary data [file bmjopen-2022-068966supp003.pdf]

Oxford Vaccine Group
University of Oxford
Centre for Clinical Vaccinology and Tropical Medicine,
Churchill Hospital, Headington, Oxford OX3 7LE
Tel: 01865 611400 info@ovg.ox.ac.uk www.ovg.ox.ac.uk

Development of a vaccine against *Salmonella* Paratyphi A (VASP)

Informed Consent Form

Participant's Name: ____

Participant Initials: __________

Participant screening number: | S | P | T | — | 0 | 6 | — | | | | |

Name of Researcher: ***If you agree, please initial box:***

**Section 1: Study Procedures.**	
1. I confirm that I have read the information sheet dated................... (version............) for this study. I have had the opportunity to consider the information, ask questions and have had these answered satisfactorily	
2. I have spoken with Dr/Nurse____________________________________	
3. I understand that my participation is voluntary and that I am free to withdraw at any time without giving any reason, without my medical care or legal rights being affected.	
4. I have received detailed information about the treatment schedule and potential side effects.	
5. I agree to be randomised to receive either the experimental vaccine CVD 1902 or the placebo and I am aware of the risks and side effects associated with vaccination	
6. Should I wish to withdraw after I have been challenged with *Salmonella* Paratyphi A, I understand that I must take a course of antibiotics and will be asked to attend for further visits for safety reasons and that if I fail to do so United Kingdom Health Security Agency (UKHSA) will be informed.	
7. I understand that should I fail to return for review or to take the full course of antibiotics I may become seriously ill and could even die.	
8. I will bring the 24-hour contact reply slip to the first study visit, signed by my 24-hour contact. I agree that the study team may contact this person if I cannot be contacted during the study	

McCann N, *et al. BMJ Open* 2023; 13:e068966. doi: 10.1136/bmjopen-2022-068966

9. I agree to refrain from donating blood for the duration of the study.	
**Section 2: Personal Information:**	
10. I agree to my GP being informed of my participation in this study including information about diagnosis, treatment and clearance samples.	
11. I agree to my GP and/or other treating doctors being approached for additional information regarding my medical and vaccination history and study staff to access my NHS medical records.	
12. I understand that United Kingdom Health Security Agency (UKHSA) will be informed of my participation in this study including information about diagnosis, treatment and clearance samples.	
13. I understand that relevant sections of my medical notes and data collected during the study may be looked at by individuals from University of Oxford, from regulatory authorities [and from the NHS Trust(s)], where it is relevant to my taking part in this research. I give permission for these individuals to have access to my records.	
14. I understand TOPS is the Health Research Authority database that aims to prevent healthy volunteers from taking part in too many studies. I understand that only staff at OVG and other research units can use the database and OVG may call other units, or OVG may be called, to check volunteer details. I agree to my National Insurance (if UK citizen) or Passport number being used to register me on TOPS. I understand that it will be stored electronically for the duration of the study.	
**Section 3: Research Samples**	
15. I agree to donate saliva, blood and stool samples. I consider these samples a gift to the University of Oxford and I understand I will not gain any direct personal or financial benefit from them.	
16. I understand that my anonymised data and biological samples may be sent and stored within and outside of the European Union for analysis by collaborating research groups as described in the information booklet	
17. I understand and agree that my samples will be used in research aimed at understanding the genetic influences on *Salmonella* Paratyphi A and that the results of these investigations are unlikely to have any implications for me personally.	
**Section 4: Occupational**	
18. I understand that I should not be involved in commercial food handling until I am shown not to be infected with *Salmonella* Paratyphi A	

McCann N, *et al. BMJ Open* 2023; 13:e068966. doi: 10.1136/bmjopen-2022-068966

19. I understand my occupation must not involve direct contact with young children (defined as those attending pre-school groups or nursery or aged under 2 years) or patient contact in a health or social care setting until I am shown not to be infected with *Salmonella* Paratyphi A		
**Section 5: Additional**:		
**20.** I understand I should not have a household contact who is immunocompromised or children under the age of 2 or older children who are attending pre-school or nursery		
21. **For those involved in the provision of health or social care to vulnerable groups only:** I agree to my employer being informed of my participation in the trial.	 N/A	
22. **Women only:** I understand the need to ensure that I or my partner use effective contraception one month prior to vaccination and continue to do so until I am shown not to be infected with *Salmonella* Paratyphi A. I also understand that if I use oral hormonal contraception there is a need to use barrier contraception from vaccination until I am shown to be cleared of infection.	 N/A	
23. I agree to take part in this study.		
24. I agree to be contacted about other ethically approved research studies for which I may be suitable. I understand that agreeing to be contacted does not oblige me to participate in any further studies.	Yes	No

Name of Participant	*Date*	*Signature*
Name of Person taking Consent	*Date*	*Signature*

**1 copy for participant; Original for participant CRF;*

McCann N, *et al. BMJ Open* 2023; 13:e068966. doi: 10.1136/bmjopen-2022-068966
