# Supplementary material for: Accelerating clinical development of a live attenuated vaccine against Salmonella Paratyphi A (VASP): study protocol for an observer-participant-blind randomised control trial of a novel oral vaccine using a human challenge model of Salmonella Paratyphi A infection in healthy adult volunteers
Source: BMJ Open. 2023 May 23;13(5):e068966. doi: 10.1136/bmjopen-2022-068966 (PMC10230971; doi:10.1136/bmjopen-2022-068966)

Supplementary Material 4: Visit Structure

Figure 1: Visit Structure of Trial

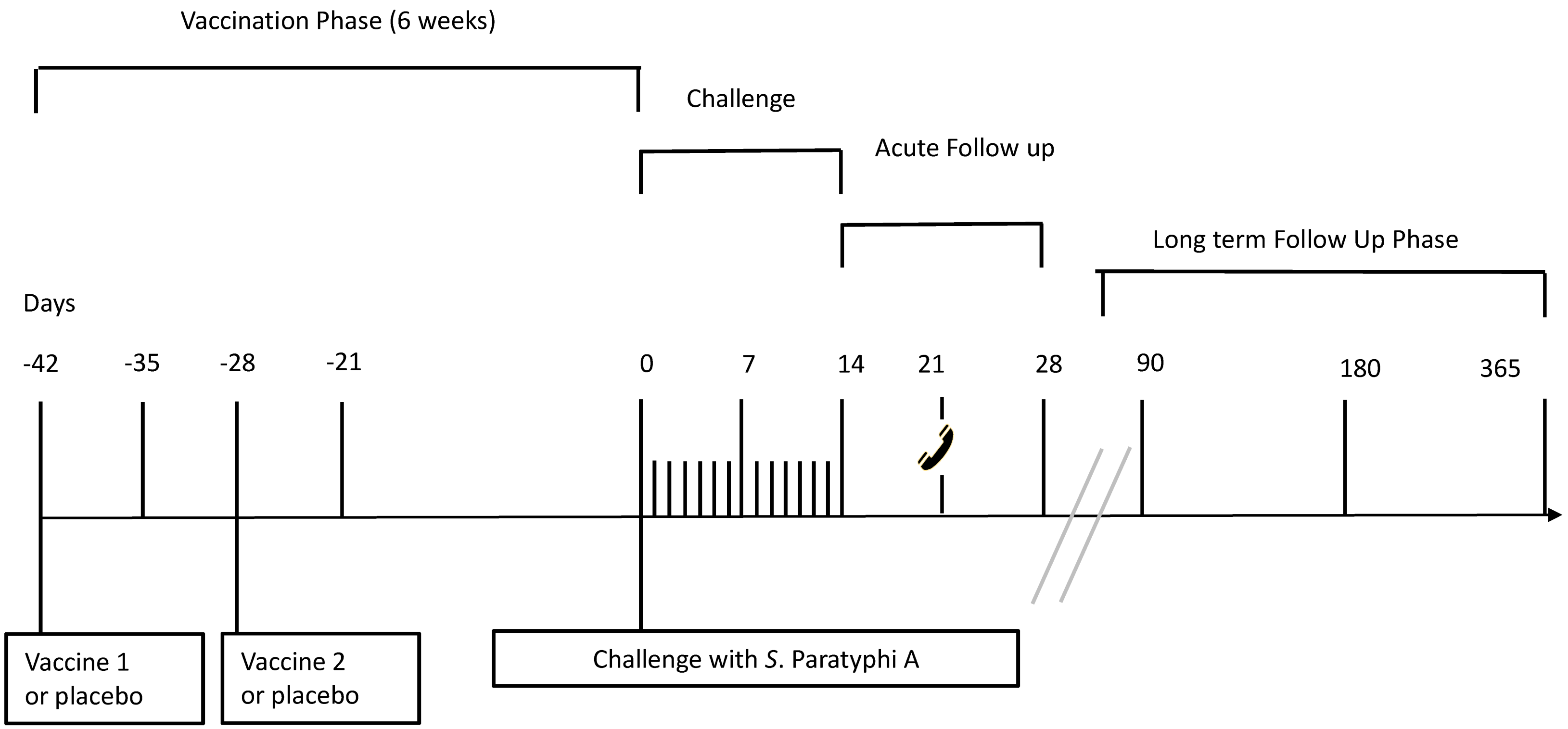

Figure 2: Structure of PD visits

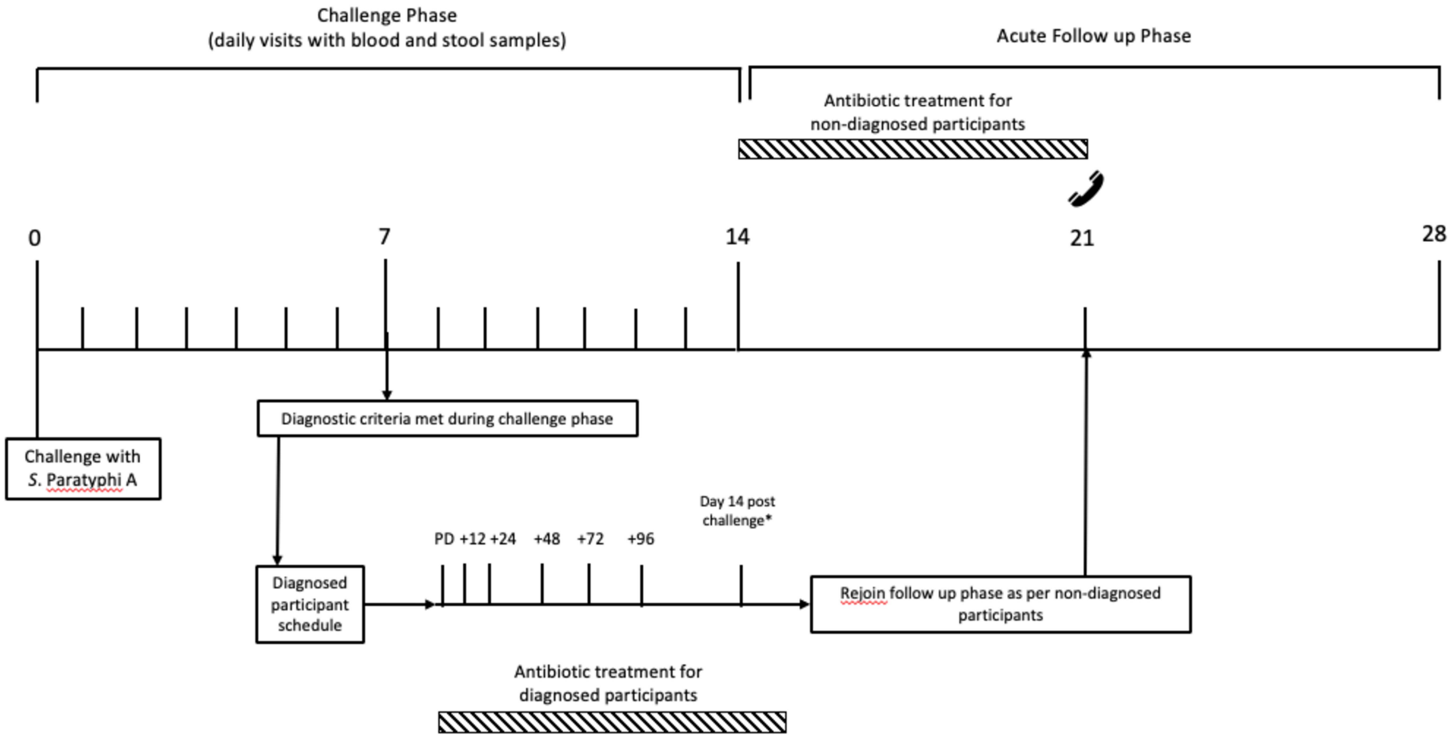

Supplement: Supplementary data [file bmjopen-2022-068966supp004.pdf]
