# Supplementary material for: Accelerating clinical development of a live attenuated vaccine against Salmonella Paratyphi A (VASP): study protocol for an observer-participant-blind randomised control trial of a novel oral vaccine using a human challenge model of Salmonella Paratyphi A infection in healthy adult volunteers
Source: BMJ Open. 2023 May 23;13(5):e068966. doi: 10.1136/bmjopen-2022-068966 (PMC10230971; doi:10.1136/bmjopen-2022-068966)
Supplement: Supplementary data [file bmjopen-2022-068966supp005.pdf]

Supplementary Material 5: Vaccination e-diary

Sample of Daily Vaccination e-diary

Remember to measure your temperature **twice a day** following vaccination

	Day of First Vaccine	
Date		
AM/PM	AM	PM
Time		
Oral (mouth) temperature	ºC	ºC

Symptom	Severity				
Headache	0	1	2	3	4
Generally unwell	0	1	2	3	4
Loss of appetite	0	1	2	3	4
Abdominal/stomach pain	0	1	2	3	4
Nausea or vomiting	0	1	2	3	4
Diarrhoea	0	1	2	3	4

If you have not experienced a symptom please circle "0" on your diary page for the relevant day. If you have experienced a symptom please circle the number indicating the severity of the symptom using the scale.

Symptom	Severity			
	1	2	3	4
Headache	Present but no interference with activity	Some interference with activity	Significant; prevents daily activity	Emergency department visit or hospitalisation
Generally unwell	Present but no interference with activity	Some interference with activity	Significant; prevents dailyactivity	Emergency department visit or hospitalisation
Eating less than usual or loss of appetite	Eat less than normal for 1-2 meals	Miss 1-2 meals completely	Miss all meals	Emergency department visit or hospitalisation
Abdominal/ stomach pain	Present but no interference with activity	Some interference with activity	Significant; any use of codeine phosphate or prevents daily activity	Emergency department visit or hospitalisation
Nausea/vomiting	Present but no interference with activity or 1 – 2 episodes in 24 hours	Some interference with activity or more than 2 episodes in 24 hours	Significant; Prevents daily activity	Emergency department visit or hospitalisation
Diarrhoea	3-4 loose stools in 24 hrs	5-6 loose stools in 24 hrs	7 or more loose stools in 24 hrs	Emergency department visit or hospitalisation

BMJ Publishing Group Limited (BMJ) disclaims all liability and responsibility arising from any reliance placed on this supplemental material which has been supplied by the author(s)

If you feel feverish at any other time please check your temperature and record below:

Date								
Day (post vaccine)								
Time								
Oral (mouth) temperature	ºC	ºC	ºC	ºC	ºC	ºC	ºC	ºC

Date								
Day (post vaccine)								
Time								
Oral (mouth) temperature	ºC	ºC	ºC	ºC	ºC	ºC	ºC	ºC

Additional symptoms

nay experience other symptoms such as feeling hot or cold, sweating, shivering or constipation eel unable to cope with any of the symptoms you are experiencing, please contact the study tea diately.

e record any additional symptoms on the following tables. Please include any illness/injury even i ink it is not related to the vaccine. Please include the start date, start time, finish date, finish time ate ongoing if not resolved by day 28), and severity of symptoms (see below scale).

e grade any symptoms using the following scale:

1	Mild – easily tolerated with no limitation on normal activity
2	Moderate – some limitation of daily activity
3	Severe – unable to perform normal daily activity
4	Emergency department or hospital visit required

**Start Date/time**	**Symptom details**	**Severity** (please circle)	**Finish date/time** (state ongoing if continu
__/__/__ __:__		1 2 3 4	__/__/__ __:__
__/__/__ __:__		1 2 3 4	__/__/__ __:__
__/__/__ __:__		1 2 3 4	__/__/__ __:__
__/__/__ __:__		1 2 3 4	__/__/__ __:__
__/__/__ __:__		1 2 3 4	__/__/__ __:__
__/__/__ __:__		1 2 3 4	__/__/__ __:__

**Date**	**(24 hr clock)**	**Medication**	**Dose**	**Reason for takin**

BMJ Publishing Group Limited (BMJ) disclaims all liability and responsibility arising from any re
placed on this supplemental material which has been supplied by the author(s)
