# Supplementary material for: Accelerating clinical development of a live attenuated vaccine against Salmonella Paratyphi A (VASP): study protocol for an observer-participant-blind randomised control trial of a novel oral vaccine using a human challenge model of Salmonella Paratyphi A infection in healthy adult volunteers
Source: BMJ Open. 2023 May 23;13(5):e068966. doi: 10.1136/bmjopen-2022-068966 (PMC10230971; doi:10.1136/bmjopen-2022-068966)
Supplement: Supplementary data [file bmjopen-2022-068966supp006.pdf]

Supplementary Material 6: Challenge e-diary

Remember to measure your temperature **twice a day** following challenge

Day 0

	Day 0	
Date		
AM/PM	AM	PM
Time		
Oral (mouth) temperature	ºC	ºC

**Symptom**	**Severity**				
Headache	0	1	2	3	4
Generally unwell	0	1	2	3	4
Loss of appetite	0	1	2	3	4
Abdominal/stomach pain	0	1	2	3	4
Nausea or vomiting	0	1	2	3	4
Muscle pain	0	1	2	3	4
Joint pain	0	1	2	3	4
Cough	0	1	2	3	4
Diarrhoea	0	1	2	3	4
Constipation	0	1	2	3	4
Rash	YES/ NO delete as appropriate	Describe where, colour, size (mm):			

If you have not experienced a symptom please circle "0" on your diary page for the relevant day. If you have experienced a symptom please circle the number indicating the severity of the symptom using the scale.

Symptom	Severity			
	1	2	3	4
Headache	Present but no interference with activity	Some interference with activity	Significant; any use of codeine phosphate or prevents daily activity	Emergency department visit or hospitalisation
Generally unwell	Present but no interference with activity	Some interference with activity	Significant; prevents daily activity	Emergency department visit or hospitalisation
Eating less than usual or loss of appetite	Eat less than normal for 1-2 meals	Miss 1-2 meals completely	Miss all meals	Emergency department visit or hospitalisation
Abdominal/ stomach pain	Present but no interference with activity	Some interference with activity	Significant; any use of codeine phosphate or prevents daily activity	Emergency department visit or hospitalisation
Nausea/ vomiting	Present but no interference with activity or 1 – 2 episodes in 24 hours	Some interference with activity or more than 2 episodes in 24 hours	Significant; Prevents daily activity	Emergency department visit or hospitalisation

Symptom	Severity			
	1	2	3	4
Muscle pain	Present but no interference with activity	Some interference with activity	Significant; prevents daily activity	Emergency department visit or hospitalisation
Joint pain	Present but no interference with activity	Some interference with activity	Significant; prevents daily activity	Emergency department visit or hospitalisation

Cough	Present but no interference with activity	Some interference with activity	Significant; prevents daily activity	Emergency department visit or hospitalisation
Diarrhoea	3-4 loose stools in 24 hrs (loose stool is defined as ≥200ml volume)	5-6 loose stools in 24 hrs (loose stool is defined as ≥200ml volume)	7 or more loose stools in 24 hrs (loose stool is defined as ≥200ml volume)	Emergency department visit or hospitalisation
Constipation	Present but no interference with activity	Some interference with activity	Significant; prevents daily activity	Emergency department visit or hospitalisation

BMJ Publishing Group Limited (BMJ) disclaims all liability and responsibility arising from any reliance placed on this supplemental material which has been supplied by the author(s)

If you feel feverish at any other time please check your temperature and record below:

Date								
Day (post challenge)								
Time								
Oral (mouth) temperature	ºC	ºC	ºC	ºC	ºC	ºC	ºC	ºC

Date								
Day (post challenge)								
Time								
Oral (mouth) temperature	ºC	ºC	ºC	ºC	ºC	ºC	ºC	ºC

Additional Symptoms

You may experience other symptoms such as feeling hot or cold, sweating or shivering. If you feel unable to manage any of the symptoms you are experiencing, please contact the study team immediately.

Please record any additional symptoms on the following tables. Please include any illness/injury even if you think it is not related to the vaccine or paratyphoid challenge. Please include the start date, start time, finish date, finish time (or state ongoing if not resolved by day 28), and severity of symptoms (see below scale).

Please grade any symptoms using the following scale:

- 1 Mild – easily tolerated with no limitation on normal activity
- 2 Moderate – some limitation of daily activity
- 3 Severe – unable to perform normal daily activity
- 4 Emergency department or hospital visit required

BMJ Publishing Group Limited (BMJ) disclaims all liability and responsibility arising from any reliance placed on this supplemental material which has been supplied by the author(s)

**Start Date/time**	**Symptom details**	**Severity** (please circle)	**Finish date/time** (state ongoing if continuing)
__/__/__ __:__		1 2 3 4	__/__/__ __:__
__/__/__ __:__		1 2 3 4	__/__/__ __:__
__/__/__ __:__		1 2 3 4	__/__/__ __:__
__/__/__ __:__		1 2 3 4	__/__/__ __:__
__/__/__ __:__		1 2 3 4	__/__/__ __:__
__/__/__ __:__		1 2 3 4	__/__/__ __:__

BMJ Publishing Group Limited (BMJ) disclaims all liability and responsibility arising from any reliance placed on this supplemental material which has been supplied by the author(s)

Medications:

Please record any medication taken in addition to any regular medication that you take. Please do not take any r agreed by the study team.

Date	Time (24 hr clock)	Medication	Dose	Reason for takin

BMJ Publishing Group Limited (BMJ) disclaims all liability and responsibility arising from any re
placed on this supplemental material which has been supplied by the author(s)
