# Supplementary material for: Accelerating clinical development of a live attenuated vaccine against Salmonella Paratyphi A (VASP): study protocol for an observer-participant-blind randomised control trial of a novel oral vaccine using a human challenge model of Salmonella Paratyphi A infection in healthy adult volunteers
Source: BMJ Open. 2023 May 23;13(5):e068966. doi: 10.1136/bmjopen-2022-068966 (PMC10230971; doi:10.1136/bmjopen-2022-068966)
Supplement: Supplementary data [file bmjopen-2022-068966supp007.pdf]

Supplementary Material 7: Sample testing during VASP Trial

Table 1: Sample collection - screening

Screening		
	Specimen	Container
FBC, ESR	Blood	EDTA vacutainer
Urea, Electrolytes & Creatinine	Blood	Heparin vacutainer
HIV, HBsAg, HCVA	Blood	Serum vacutainer
TTG and IgA	Blood	Serum vacutainer
HLA B27	Blood	EDTA vacutainer
Total blood volume		Maximum 17 mL
Blood glucose	Blood	NA
Urine dipstick	Urine	Standard urine pot
Urine Pregnancy test	Urine	Standard urine pot

Table 2: Sample collection – vaccination and post vaccination

Vaccine	Investigation	Pregnancy Test	Saliva	Stool sample	Blood culture	Full blood count	CRP, U+Es, LFTs	Blood for OVG laboratories	
	Sample tube	std urine pot	saliva coll-ection device	Stool Pot	Aerobic BACTEC bottle	EDTA Vacutainer	Heparin Vacutainer		
	**Volume blood (mL)**				10	1 → 3	2		**TOTAL***
	**Day**								
1	**D-42 (Va)**	x	x	x		2	3	74.5	79.5
	**D-35 (Vb)**			x	10	2	3	32.5	47.5
2	**D-28 (Vc)**	x	x	x	10	2	3	71	86
	**D-21 (Vd)**			x	10	2	3	32.5	47.5
								Total	260.5

Specific volumes and sample tubes for different assays for the OVG laboratories will be detailed in the Laboratory Analysis Plan and Clinical Study Plan.

Table 3: Sample collection – challenge period

Challenge	Investigation	SARS-CoV-2 Test	Pregnancy Test	Saliva	Stool sample	Blood culture	Full blood count	CRP, U+Es, LFTs	Blood for OVG laboratories	
	Sample tube	NP swab	std urine pot	saliva coll-ection device	Stool Pot	Aerobic BACTEC bottle	EDTA Vacutainer	Heparin Vacutainer		
	**D-2**	x								
→	**D0**		x	x	x	10	2	3	81	96
	**D1**				x	10			20	30
	**D2**				x	10	2	3	up to 4	19
	**D3**				x	10			0	10
	**D4**				x	10	2	3	up to 4	19
	**D5**				x	10			0	10
	**D6**				x	10	2	3	0	15
	**D7**				x	10	2		58.5	70.5
	**D8**				x	10	2	3	0	15
	**D9**				x	10			0	10
	**D10**				x	10	2	3	0	15
	**D11**				x	10			0	10
	**D12**				x	10	2	3	0	15
	**D13**				x	10			0	10
	**D14**	x	x		x	10	2	3	58.5	73.5
									Total*	418

Specific volumes and sample tubes for different assays for the OVG laboratories will be detailed in the Laboratory Analysis Plan and Clinical Study Plan.

Table 4: Sample collection – paratyphoid diagnosis, follow up and totals

Investigation	SARS-CoV-2 Test	Pregnancy Test	Saliva	Stool sample	Blood culture	Full Blood count	CRP, U+Es, LFTs	Blood for OVG laboratories	
Sample tube	NP swab	std urine pot	saliva coll-ection device	Stool Pot	Aerobic BACTEC bottle	EDTA Vacutain er	Heparin Vacutain er		
**Day**									**TOTAL**
**PD**	x	x		x	10	2	3	12.5	27.5
**PD +12hrs**				x	10	2		50	62
**PD +24hrs**				x	10	2	3	**	15
**PD +48hrs**				x	10	2	3	0	15
**PD +72hrs**				x	10	2	3	0	15
**PD +96hrs**				x	10	2	3	50	65
**D14PD gp**				x		2	3	0	5
**D28**			x	x		2	3	58.5	63.5
								Total	268
	**Maximum total for first 3 months (excluding screening)**			**If Paratyphoid NOT diagnosed**				**Total**	**738**
	**Maximum total for first 3 months (excluding screening)**			**If Paratyphoid Diagnosed (maximum blood volume)**				**Total**	**937.5**
**D90**			x	x		2	3	58.5	63.5
**D180**			x	x		2	3	48.5	53.5
**D365**			x	x		2	3	58.5	63.5
	**Maximum total in 14 months (excluding screening)**			**If Paratyphoid NOT diagnosed**				**Total**	**922.5**
	**Maximum total in 14 months (excluding screening)**			**If Paratyphoid Diagnosed (maximum blood volume)**				**Total**	**1122**

Specific volumes and sample tubes for different assays for the OVG laboratories will be detailed in the Laboratory Analysis Plan and Clinical Study Plan.
