# Supplementary material for: Accelerating clinical development of a live attenuated vaccine against Salmonella Paratyphi A (VASP): study protocol for an observer-participant-blind randomised control trial of a novel oral vaccine using a human challenge model of Salmonella Paratyphi A infection in healthy adult volunteers
Source: BMJ Open. 2023 May 23;13(5):e068966. doi: 10.1136/bmjopen-2022-068966 (PMC10230971; doi:10.1136/bmjopen-2022-068966)
Supplement: Supplementary data [file bmjopen-2022-068966supp008.pdf]

Supplementary Material 8: Medications used in the VASP trial

Table 1: Concomitant medications used

Drug	Indication	Dose	Route	Frequency
Paracetamol	Fever and discomfort (**after antibiotic therapy started**)	500mg - 1 Gram	Oral	PRN, max QDS
Codeine	Pain including headache	15-60mg	Oral	PRN (max. 240mg/24 hours)
Senna	Constipation	2-4 tablets	Oral	PRN
Cyclizine	Nausea and/or vomiting	50mg	Oral	PRN (max. 150mg/24 hours)
Chlorpheniramine	Allergy	4mg	Oral	PRN TDS-QDS (max. 24mg/24 hours)
Oral rehydration salts	Dehydration, vomiting or diarrhoea	1-2 sachets	Oral	PRN
Sando-K	Hypokalaemia	2-4 tablets	Oral	PRN, up to TDS dependent on potassium deficit

Table 2: Antibiotics used

Drug	Dose	Route	Frequency	Duration
Ciprofloxacin	500mg	Oral	BD	7 days
Co-trimoxazole	160/800mg	Oral	BD	7 days
Azithromycin	1g stat followed by 500mg	Oral	OD	7 days
Amoxicillin	500mg	Oral	TDS	7 days
