# Supplementary material for: Accelerating clinical development of a live attenuated vaccine against Salmonella Paratyphi A (VASP): study protocol for an observer-participant-blind randomised control trial of a novel oral vaccine using a human challenge model of Salmonella Paratyphi A infection in healthy adult volunteers
Source: BMJ Open. 2023 May 23;13(5):e068966. doi: 10.1136/bmjopen-2022-068966 (PMC10230971; doi:10.1136/bmjopen-2022-068966)
Supplement: Supplementary data [file bmjopen-2022-068966supp009.pdf]

Supplementary Material 9: Trial Stopping Rules

Safety holding Rules

In the event of any of the following, vaccination of further individuals will be paused pending DSMC review:

- New scientific information is published to indicate that subjects in the trial are being exposed to undue risks as a result of administration of the IMP, or as a result of the trial procedures or follow-up schedule.
- Bacteraemia with *Salmonella* Paratyphi A of any participant during the vaccination period
- Serious concerns about the safety of the IMP arise as a result of one or more vaccine related SAE(s) occurring in the subjects enrolled
- If at least two subjects develop a 'severe' adverse event related to the study drug, as assessed by a clinician, independent of within or not within the same-organ-class.

The DSMC chair will then undertake a review of the data to decide whether a temporary halt is required as an urgent safety measure and what the scope of the halt will be. A full meeting of the DSMC can be called at the chair's discretion. If, following DSMC review, a halt is required, the Sponsor, REC and appropriate regulatory authorities will be notified within 3 days and a substantial amendment submitted within 15 days. If the DSMC decide that a halt is not required then the trial may continue.

Following a halt to the trial, if it is decided that the trial may re-start, then a request and substantial amendment will be made to the Sponsor, the REC and the MHRA in order to do so, otherwise the trial will be terminated.

Individual stopping Rules

Stopping rules for individual volunteers will apply (i.e., indications to withdraw individuals from further vaccinations). Study participants who present with at least one of the following stopping rules will trigger a clinical review as to whether the participant should be withdrawn from further vaccination in the study, would not undergo challenge and would be followed up only from a safety perspective if participants consent to this.

- Laboratory Aes: the participant develops a confirmed $\geq$ grade 3 laboratory AE considered possibly, probably or definitely related within 7 days after vaccination
- Solicited adverse events: the participant develops a $\geq$ grade 3 systemic solicited AE considered possibly, probably or definitely related within 2 days after vaccination (day of vaccination and one subsequent day) which is deemed severe by clinician assessment
- Unsolicited adverse events: the participant has a $\geq$ grade 3 adverse event, considered possibly, probably or definitely related to vaccination which is deemed severe by clinician assessment or has a SAE considered possibly, probably or definitely related to vaccination.

- The participant has an acute allergic reaction or anaphylactic shock following the administration of the vaccine investigational product.
