# Supplementary material for: Accelerating clinical development of a live attenuated vaccine against Salmonella Paratyphi A (VASP): study protocol for an observer-participant-blind randomised control trial of a novel oral vaccine using a human challenge model of Salmonella Paratyphi A infection in healthy adult volunteers
Source: BMJ Open. 2023 May 23;13(5):e068966. doi: 10.1136/bmjopen-2022-068966 (PMC10230971; doi:10.1136/bmjopen-2022-068966)
Supplement: Supplementary data [file bmjopen-2022-068966supp010.pdf]

Supplementary material 10: Adverse Event grading

Adverse event	Grade	Definition (in degrees Celsius)
Temperature	0	< 37.6
	1	37.6 – 38.0
	2	38.1 – 39.0
	3	> 39.0

Participant grading of severity (vaccine phase)

	0	1	2	3	4
**Nausea /vomiting**	No symptoms	Present but no interference with activity or 1 – 2 episodes in 24 hours	Some interference with activity or more than 2 episodes in 24 hours	Significant; prevents daily activity	Emergency department visit or hospitalisation
**Diarrhoea**	No symptoms	3-4 loose stools in 24 hrs	5-6 loose stools in 24 hrs	7 or more loose stools in 24 hrs	Emergency department visit or hospitalisation
**Eating less than usual or loss of appetite**	No symptoms	Eat less than normal for 1-2 meals	Miss 1-2 meals completely	Miss all meals	Emergency department or hospital visit required
**Generally unwell**	No symptoms	Present but no interference with activity	Some interference with activity	Significant; prevents daily activity	Emergency department visit or hospitalisation
**Abdominal / stomach pain**	No symptoms	Present but no interference with activity	Some interference with activity	Significant; prevents daily activity	Emergency department visit or hospitalisation
**Headache**	No symptoms	Present but no interference with activity	Some interference with activity	Significant; prevents daily activity	Emergency department visit or hospitalisation

Participant grading of severity (challenge phase)

	0	1	2	3	4
**Headache**	No symptoms	Present but no interference with activity	Some interference with activity	Significant; any use of codeine phosphate or prevents daily activity	Emergency department visit or hospitalisation
**Generally unwell**	No symptoms	Present but no interference with activity	Some interference with activity	Significant; prevents daily activity	Emergency department visit or hospitalisation
**Eating less than usual or loss of appetite**	No symptoms	Eat less than normal for 1-2 meals	Miss 1-2 meals completely	Miss all meals	Emergency department or hospital visit required
**Abdominal/ stomach pain**	No symptoms	Present but no interference with activity	Some interference with activity	Significant; any use of codeine phosphate or prevents daily activity	Emergency department visit or hospitalisation
**Nausea/vomiting**	No symptoms	Present but no interference with activity or 1 – 2 episodes in 24 hours	Some interference with activity or more than 2 episodes in 24 hours	Significant; prevents daily activity	Emergency department visit or hospitalisation
**Muscle pain**	No symptoms	Present but no interference with activity	Some interference with activity	Significant; any use of codeine phosphate or prevents daily activity	Emergency department visit or hospitalisation
**Joint pain**	No symptoms	Present but no interference with activity	Some interference with activity	Significant; any use of codeine phosphate or prevents daily activity	Emergency department visit or hospitalisation
**Cough**	No symptoms	Present but no interference with activity	Some interference with activity	Significant; any use of codeine phosphate or prevents daily activity	Emergency department visit or hospitalisation

**Diarrhoea**	No symptoms	3-4 loose stools in 24 hrs	5-6 loose stools in 24 hrs	7 or more loose stools in 24 hrs	Emergency department visit or hospitalisation
**Constipation**	No symptoms	Present but no interference with activity or 1 – 2 episodes in 24 hours	Some interference with activity or more than 2 episodes in 24 hours	Significant; prevents daily activity	Emergency department visit or hospitalisation
**Rash**	No symptoms	Yes/No			

Grading the severity of visit observed Adverse Events

Observation	Grade 1	Grade 2	Grade 3	Grade 4
Oral temperature (℃)	37.6 – 38.0	38.1 – 39.0	> 39.0	A&E visit or hospitalisation for hyperpyrexia
Tachycardia (beats/min)	101-115	116-130	>130	A&E visit or hospitalisation for arrhythmia
Bradycardia (beats/min)	50-54	45-49	<45	A&E visit or hospitalisation for arrhythmia
Systolic hyper-tension (mmHg)	141-150	151-155	>155	A&E visit or hospitalization for malignant hypertension
Diastolic hyper-tension (mmHg)	91-95	96-100	>100	A&E visit or hospitalization for malignant hypertension
Systolic hypo-tension (mmHg)	85-89	80-84	<80	A&E visit or hospitalization for hypotensive shock

Grading the severity of laboratory Adverse Events

Parameter	Grade 1	Grade 2	Grade 3	Grade 4*
**Haemoglobin: decrease from baseline value (g/l)**	10 - 15	16-20	21-50	>50
**White cell count: elevated (10^9/L)**	11–15	16–20	21–25	>25
**White cell count: depressed (10^9/L)**	2.5-3.5	1.5-2.4	1.0-1.4	<1.0
**Neutrophil count (10^9/L)**	1.5-2.0	1.0-1.4	0.5-0.9	<0.5
**Platelets (10^9/L)**	125-140	100-124	25-99	<25
**Sodium: hyponatraemia (mmol/L)**	132–134	130–131	125–129	<125
**Sodium: hypernatraemia (mmol/L)**	146	147	148–150	>150
**Potassium: hyperkalaemia (mmol/L)**	5.1–5.2	5.3–5.4	5.5–5.6	>5.6
**Potassium: hypokalaemia (mmol/L)**	3.3–3.4	3.1–3.2	3.0	<3.0
**Urea (mmol/L)**	8.2–8.9	9.0–11	>11	RRT
**Creatinine (µmol/L)**	132-150	151-176	177-221	>221 or RRT
**ALT and/or AST (IU/L)**	1.1–2.5 x ULN	>2.6–5.0 x ULN	5.1-10 x ULN	>10 x ULN
**Bilirubin, with increase in LFTs (µmol/L)**	1.1–1.25 x ULN	1.26–1.5 x ULN	1.51–1.75 x ULN	>1.75 x ULN
**Bilirubin, with normal LFTs (µmol/L)**	1.1–1.5 x ULN	1.6–2.0 x ULN	2.1–3.0 x ULN	>3.0 x ULN
**Alkaline phosphatase (IU/L)**	1.1–2.0 x ULN	2.1–3.0 x ULN	3.1–10 x ULN	>10 x ULN
**Albumin: hypoalbuminaemia (g/L)**	28–31	25–27	<25	Not applicable
**C-reactive protein**	>10-30	31-100	101-200	>200

Grade 4* Potentially life threatening
