# Supplementary material for: Accelerating clinical development of a live attenuated vaccine against Salmonella Paratyphi A (VASP): study protocol for an observer-participant-blind randomised control trial of a novel oral vaccine using a human challenge model of Salmonella Paratyphi A infection in healthy adult volunteers
Source: BMJ Open. 2023 May 23;13(5):e068966. doi: 10.1136/bmjopen-2022-068966 (PMC10230971; doi:10.1136/bmjopen-2022-068966)
Supplement: Author's manuscript [file bmjopen-2022-068966.draft_revisions.pdf]

BMJ Open

BMJ Open is committed to open peer review. As part of this commitment we make the peer review history of every article we publish publicly available.

When an article is published we post the peer reviewers' comments and the authors' responses online. We also post the versions of the paper that were used during peer review. These are the versions that the peer review comments apply to.

The versions of the paper that follow are the versions that were submitted during the peer review process. They are not the versions of record or the final published versions. They should not be cited or distributed as the published version of this manuscript.

BMJ Open is an open access journal and the full, final, typeset and author-corrected version of record of the manuscript is available on our site with no access controls, subscription charges or pay-per-view fees (http://bmjopen.bmj.com).

If you have any questions on BMJ Open's open peer review process please email info.bmjopen@bmj.com

BMJ Open

Accelerating Clinical Development of a Live Attenuated Vaccine against Salmonella Paratyphi A: Study protocol for an observer-participant-blind randomised control trial of a novel oral vaccine using a human challenge model of Salmonella Paratyphi A infection in healthy adult volunteers

Journal:	*BMJ Open*
Manuscript ID	bmjopen-2022-068966
Article Type:	Protocol
Date Submitted by the Author:	07-Oct-2022
Complete List of Authors:	McCann, Naina; University of Oxford Oxford Vaccine Group, Department of Paediatrics; NIHR Oxford Biomedical Research Centre<br>Emary, Katherine; University of Oxford Oxford Vaccine Group, Department of Paediatrics; NIHR Oxford Biomedical Research Centre<br>Singh, Nisha; University of Oxford Oxford Vaccine Group, Department of Paediatrics; NIHR Oxford Biomedical Research Centre<br>Mclean, Florence; University of Oxford Oxford Vaccine Group, Department of Paediatrics; NIHR Oxford Biomedical Research Centre<br>Camara, Susana; University of Oxford, Oxford Vaccine Group, Department of Paediatrics; NIHR Oxford Biomedical Research Centre<br>Jones, Elizabeth; University of Oxford Oxford Vaccine Group, Department of Paediatrics; NIHR Oxford Biomedical Research Centre<br>Kim, Young Chan; University of Oxford Oxford Vaccine Group, Department of Paediatrics; NIHR Oxford Biomedical Research Centre<br>Liu , Xinxue ; University of Oxford Oxford Vaccine Group, Department of Paediatrics ; NIHR Oxford Biomedical Research Centre<br>Greenland, Melanie ; University of Oxford Oxford Vaccine Group, Department of Paediatrics ; NIHR Oxford Biomedical Research Centre<br>Conlin, Kerry; University of Oxford Oxford Vaccine Group, Department of Paediatrics; NIHR Oxford Biomedical Research Centre<br>Hill, Jennifer; University of Oxford Oxford Vaccine Group, Department of Paediatrics ; NIHR Oxford Biomedical Research Centre<br>Verheul, Marije; University of Oxford Oxford Vaccine Group, Department of Paediatrics; NIHR Oxford Biomedical Research Centre<br>Robinson, Hannah; University of Oxford Oxford Vaccine Group, Department of Paediatrics; NIHR Oxford Biomedical Research Centre<br>Angus, Brian; University of Oxford Oxford Vaccine Group, Department of Paediatrics ; NIHR Oxford Biomedical Research Centre<br>Ramasamy, Maheshi ; University of Oxford Oxford Vaccine Group, Department of Paediatrics ; NIHR Oxford Biomedical Research Centre<br>Levine, Myron M ; University of Maryland School of Medicine, Center for Vaccine Development and Global Health<br>Pollard, Andrew; University of Oxford Oxford Vaccine Group, Department of Paediatrics ; NIHR Oxford Biomedical Research Centre
Keywords:	INFECTIOUS DISEASES, MICROBIOLOGY, Clinical trials < THERAPEUTICS

For peer review only - http://bmjopen.bmj.com/site/about/guidelines.xhtml

BMJ Open Access Protocol

Title: Accelerating Clinical Development of a Live Attenuated Vaccine against *Salmonella* Paratyphi A: Study protocol for an observer-participant-blind randomised control trial of a novel oral vaccine using a human challenge model of *Salmonella* Paratyphi A infection in healthy adult volunteers

Authors

Naina McCann [1,2]
Katherine Emary [1,2]
Nisha Singh [1,2]
Florence Mclean [1,2]
Susana Camara [1,2]
Elizabeth Jones [1,2]
Young Chan Kim [1,2]
Xinxue Li [1,2]
Melanie Greenland [1,2]
Kerry Conlin [1,2]
Jennifer Hill [1,2]
Marije Verheul [1,2]
Hannah Robinson[1,2]
Brian Angus [1,2]
Maheshi Ramasamy [1,2]
Myron M Levine[3]
Andrew J Pollard[1,2]

1- Oxford Vaccine Group, Department of Paediatrics, University of Oxford
2- NIHR Oxford Biomedical Research Centre, Oxford, UK
3- Department of Paediatrics, Center for Vaccine Development and Global Health, University of Maryland School of Medicine, Baltimore, MD, USA; Department of Medicine, Center for Vaccine Development and Global Health, University of Maryland School of Medicine, Baltimore, MD, USA

Corresponding author:
Dr Naina McCann
Naina.mccann@paediatrics.ox.ac.uk
07717890910

For peer review only

Present address: Oxford Vaccine Group, Centre for Clinical Vaccinology and Tropical Medicine, (CCVTM), Churchill Hospital, Old Road, Headington, Oxford, United Kingdom OX3 7LE

Keywords

Salmonella Paratyphi

Vaccine

Enteric fever

Challenge model

Word count: 3427

Abstract

Introduction

This is the first efficacy study of an oral live attenuated vaccine against *Salmonella* Paratyphi A using a human challenge model of paratyphoid infection. *Salmonella* Paratyphi A is responsible for 3.3 million cases of enteric fever every year, with over 19,000 deaths. Although improvements to sanitation and access to clean water are vital to reduce the burden of this condition, vaccination offers a cost-effective, medium-term solution. Efficacy trials of potential *S.* Paratyphi vaccine candidates in the field are unlikely to be feasible given the large number of participants required. Human challenge models therefore offer a unique, cost-effective solution to test efficacy of such vaccines.

Methods and analysis

This is an observer-blind, randomised, placebo-controlled trial Phase I/II of the oral live-attenuated vaccine against *S.* Paratyphi A, CVD 1902. Volunteers will be randomised 1:1 to receive two doses of CVD 1902 or placebo, 14 days apart. One month following second vaccination all volunteers will ingest *S.* Paratyphi A bacteria with a bicarbonate buffer solution. They will be reviewed daily in the following 14 days and diagnosed with paratyphoid infection if the pre-defined microbiological or clinical diagnostic criteria are met. All participants will be treated with antibiotics on diagnosis, or at day 14 post challenge if not diagnosed. The vaccine efficacy will be determined by comparing the relative attack rate, i.e. the proportion of those diagnosed with paratyphoid infection, in the vaccine and placebo groups.

Ethics and dissemination

Ethical approval for this study has been obtained from the Berkshire Medical Research Ethics committee (REC ref 21/SC/0330). The results will be disseminated via publication in a peer-reviewed journal and presentation at international conferences.

For peer review only - http://bmjopen.bmj.com/site/about/guidelines.xhtml

Trial registration number: ISRCTN15485902

Strengths and limitations of this study

- This study will provide the first efficacy assessment of an oral live attenuated *Salmonella* Paratyphi A vaccine candidate
- As the largest *S*. Paratyphi A human challenge study to date this study will provide insight into the immune response following oral vaccination and infection with S. Paratyphi A. This will support future vaccine development efforts against this pathogen
- The population in the trial may not be representative of the target population in endemic areas
- The model may under-estimate vaccine efficacy due to stringent diagnostic criteria

Introduction

Background

Enteric fever is a systemic febrile illness affecting 14.3 million individuals annually in 2017 (1). It is principally caused by *Salmonella enterica* serovars Typhi and Paratyphi A and is a common cause of morbidity, particularly in South and South East Asia. The proportion attributable to *Salmonella* Paratyphi A varies widely by geography but may be in the region of 25% (1), and is increasing in some areas (2–4). In the UK, S. Paratyphi represents 40-50% of travel-related enteric fever cases (5–7).

Control of enteric fever in endemic areas is challenged by a lack of sensitive diagnostic tests, rising antimicrobial resistance and limited access to safe drinking water. Vaccines are likely to be a cost-efficient way to reduce disease burden. The recent implementation of the typhoid conjugate vaccine in endemic countries is likely to have a significant impact on reducing *S*. Typhi incidence (8–10) and it is unclear what impact wider typhoid vaccination coverage may have on the rates of *S*. Paratyphi A infection (11). There are no current licensed vaccines against *S*. Paratyphi A.

Vaccine development for enteric fever has historically been hampered by the lack of an effective animal model to study the immunobiology of disease and the need for large sample sizes in efficacy trials. Controlled human infection models (CHIM) offer a unique alternative to study immunological responses to and the efficacy of such vaccines.

The Oxford Vaccine Group (OVG), has developed an enteric fever CHIM where over 400 participants have safely undergone challenge since 2010 (12–15). In recent years the OVG has developed the first

For peer review only - http://bmjopen.bmj.com/site/about/guidelines.xhtml

S. Paratyphi A human challenge model, offering an opportunity to test the efficacy and immunological response to novel *S.* Paratyphi A vaccine candidates (14).

Several vaccines against *S*. Paratyphi A are in development, employing a range of approaches (16,17). Oral live-attenuated vaccines offer several immunological and practical advantages to parenteral vaccines, including ease of administration and induction of a broad immunological response. The University of Maryland has developed such a vaccine, CVD 1902, for which a one-dose regimen has been shown to be safe and immunogenic in humans, although efficacy remains unknown (5).

Study aims and objectives

This study aims to assess the efficacy of the orally administered live-attenuated vaccine CVD 1902 and study the immune response both to *S*. Paratyphi A infection and vaccination. Primary, secondary and exploratory objectives and outcomes are outlined in Table 1.

For peer review only

120 Table 1. Primary, secondary, and exploratory objectives and outcomes of the VASP study

	Objectives	Outcome Measures
Primary	To determine the relative protective effect of two doses of CVD 1902 given 14 days apart compared with placebo (sodium bicarbonate) in a healthy adult paratyphoid challenge model	The proportion of participants developing clinical or microbiologically proven paratyphoid infection following oral challenge with *S*. Paratyphi A in the group who have received two doses of CVD 1902 compared with those who have received two doses of placebo
Secondary	a. To compare the clinical and laboratory features of the host responses following challenge with *Salmonella* Paratyphi A in participants vaccinated with CVD 1902 compared to placebo	Using clinical reporting, physical examination findings, microbiological and laboratory assays to compare the clinical course of paratyphoid infection after challenge between placebo and CVD 1902 groups
	b. To compare the host immune response following vaccination with CVD 1902, compared with placebo including innate, antibody and cell-mediated responses and persistence of immunity and to relate these responses to the protective effect of vaccination.	Immunological laboratory assays to assess innate, humoral, cell-mediated and mucosal responses to vaccination at baseline (Day -42) and post-vaccination time points
	c. To compare the host immune response following *S.* Paratyphi A challenge following vaccination with CVD 1902 or placebo.	Immunological laboratory assays to assess innate, humoral, cell-mediated and mucosal responses to challenge will be taken at various time points following challenge
	d. To assess the safety and tolerability of CVD 1902 including faecal shedding	Clinical observation and participant recording of symptoms, both solicited and unsolicited plus safety laboratory data and microbiological data from blood and stool cultures following vaccination
	e. To investigate immunological correlates of protection for *S*. Paratyphi A infection	Immunological response data post-vaccination will be combined with vaccine efficacy data following *S.* Paratyphi A challenge to investigate if particular immunological markers could be used to predict protection from paratyphoid infection

For peer review only - http://bmjopen.bmj.com/site/about/guidelines.xhtml

Methods

Study design and setting

This is an observer-blind, participant-blind, randomised, placebo-controlled trial of the oral live-attenuated vaccine CVD 1902 using a healthy adult participant controlled human infection model of paratyphoid.

In total 74-76 participants will be randomised in a 1:1 ratio to receive a dose of not less than 2 x 10^{10} CFU (Colony Forming Units) of CVD 1902 or placebo (37-38 participants per group). As outlined in Figure 1, all participants will receive two doses of vaccine or placebo 14 days apart. Twenty-eight days after their second vaccine or placebo dose participants will be challenged with *S*. Paratyphi A (strain NVGH308) at a dose of 1-5 x 10^3 CFU, the dose previously established to give a desired clinical/laboratory 'attack' rate of approximately 60%(14).

Figure 1: Study design of the VASP trial investigating an oral vaccine against S. Paratyphi A

The study will be conducted at the Centre for Clinical Vaccinology and Tropical Medicine, Oxford, UK, which is a fully equipped vaccine research site with available clinical inpatient facilities and a Category III level laboratory on-site. The UK is non-endemic for enteric fever; cases are typically travel related (18).

Recruitment and eligibility

All potential participants may be contacted by methods including but not limited to email, telephone, posters, leaflets, websites, advertisements in newspaper, radio and on social media and/or mail using an approved invitation letter or other approved advertising material to invite them to participate in the study. Participants will be reimbursed for their time, travel and inconvenience.

Male or female participants between 18 and 55 years inclusive who are in good health (as determined by medical history, physical examination and clinical judgement of study team and their general practitioner) and willing and able to provide written informed consent will be eligible for inclusion. Multiple other strict inclusion and exclusion criteria will be applied during the screening process to minimise the risk to participants and to minimise the risk of onward transmission to others. A summarised list of inclusion and exclusion criteria is provided in Boxes 1 and 2 with a complete list provided in supplementary material 1.

For peer review only - http://bmjopen.bmj.com/site/about/guidelines.xhtml

Box 1. Summary of inclusion Criteria

Inclusion Criteria

Participants must satisfy all the following criteria to be considered eligible for the study:

- Willing and able to give informed consent for participation in the study.
- Aged between 18 and 55 years inclusive at time of vaccination.
- In good health as determined by medical history, physical examination and clinical judgment of the study team.
- Willing to be available in Oxford for all required appointments
- Agree to comply with all study requirements, including capacity to adhere to good personal hygiene and infection control precautions.
- Agree to allow study staff to contact his or her GP to access the participant's records and have their opinion solicited as to the participant's appropriateness for inclusion.
- Agree to allow study staff to access NHS health records as required for study purposes.
- Agree to allow his or her GP and UKHSA to be notified of participation in the study.
- Agree to give his or her close household contacts written information informing them of the participants' involvement in the study and offering them voluntary screening for *S.* Paratyphi A carriage.
- Agree to have 24-hour contact with study staff during the four weeks post challenge and are able to ensure that they are contactable by mobile phone for the duration of the vaccination and challenge period until antibiotic completion.
- Have internet access to allow completion of the e-diary and real-time safety monitoring.
- Agree to avoid antipyretic/anti-inflammatory treatment from challenge until advised by a study doctor or until 14 days after challenge.
- Agree to refrain from donating blood for the duration of the study.
- Agree to provide their National Insurance/Passport number for the purposes of TOPS registration and for payment
- Have received at least one dose of a SARS-CoV-2 vaccine $\geq$ four weeks prior to enrollment.
- Agree to not receive other vaccinations during the 7 days before and after study vaccination and during the 7 days before or 21 days post-challenge.

For peer review only - http://bmjopen.bmj.com/site/about/guidelines.xhtml

Box 2. Summary of Exclusion Criteria

Exclusion Criteria

The participant will not be enrolled if any of the following apply:

- History of significant organ/system disease that could interfere with trial conduct or completion
- Have any known or suspected impairment of immune function, alteration of immune function, or prior immune exposure that may alter immune function to paratyphoid
- HLA-B27 positive.
- Moderate or severe depression or anxiety
- Weight less than 50 kg.
- Presence of implants or prosthetic material.
- Anyone taking long-term medication that may affect symptom reporting or interpretation of the study results.
- Contraindication to fluoroquinolones, macrolide antibiotics, co-trimoxazole or ceftriaxone.
- Family history of aneurysmal disease
- Female participants who are pregnant, lactating or who are unwilling to ensure that they use effective contraception
- Full-time, part-time or voluntary occupations involving either direct contact with young children or highly susceptible patients or persons in whom typhoid infection would have particularly serious consequences, or commercial food handling
- Close household contact with young children or individuals who are immunocompromised (including pregnancy).
- Scheduled elective surgery or other procedures requiring general anaesthesia during the study period.
- Participants who have participated in another research study involving an investigational product that might affect risk of paratyphoid infection or compromise the integrity of the study within the 30 days prior to enrolment
- Detection of any abnormal results from screening investigations (at the clinical discretion of the study team).
- Inability to comply with any of the study requirements
- Any other social, psychological or health issues which, in the opinion of the study staff, may
    - put the participant or their contacts at risk because of participation in the study,
    - adversely affect the interpretation of the primary endpoint data,
    - Impair the participant's ability to participate in the study.
- Have any history of allergy to vaccine/placebo components
- Having been resident in an enteric fever endemic country for 6 months or more.
- Have previously been diagnosed with laboratory-confirmed typhoid or paratyphoid infection or been given a diagnosis compatible with enteric fever.
- Have participated in previous typhoid or paratyphoid challenge studies
- Have received any oral typhoid vaccination at any time.
- Have a prolonged corrected QT interval (>450 milliseconds) on ECG screening.
- Significant blood donation or planned blood donation prior to enrolment.

Participants will undergo a three-stage screening process to assess their suitability of the trial and to identify any of the inclusion and exclusion criteria. An online questionnaire will be followed by a telephone screening consultation and then a face-face consultation involving a physical examination, ECG, blood tests, urine dipstick, pregnancy test for females and gallbladder ultrasound examination. Temporary exclusion criteria will also be applied on the days of vaccination and challenge, as outlined in supplementary material 2.

For peer review only - http://bmjopen.bmj.com/site/about/guidelines.xhtml

Trial Procedures

Enrolment into the study will be day of first vaccination (D-42). The detailed visit structure following enrolment can be found in the supplementary material 3.

Randomisation

Participants will be randomised 1:1 to receive CVD 1902 or placebo on Day -42. A statistician at OVG will generate a randomisation list using varying block sizes (2 and 4). The randomisation list aims to ensure equal target sample size is reached per group. The web-based randomisation system will ensure the allocation concealment is valid. Randomisation of participants will be carried out by unblinded study staff who are independent from the blinded team and do not perform any post vaccination procedures (such as ongoing eligibility, sample collection).

Blinding

This study will be conducted observer- and participant-blind from the time of randomisation until participant unblinding which will occur once the *last* participant has completed their Day 28 post-challenge visit. Observer and participant blinding is required to minimise the risk of bias on the reporting of adverse events following the administration of vaccine and challenge.

Vaccination visits

Vaccine or placebo will be administered at the D-42 visit and the D-28 visit. Participants must have fasted for the 90 minutes prior to vaccination and pre-treatment bicarbonate solution will be administered in the minute preceding vaccination to neutralise stomach acid. Participants will be directly observed for a minimum of 30 minutes following vaccination and then asked to complete an e-diary of their symptoms daily for 7 days following each vaccination (supplementary material 4).

An in-person post-vaccination review will occur 7 days following each vaccine where participants will be reviewed for any possible adverse events and provide blood and stool samples.

Challenge visits

S. Paratyphi A challenge will be administered by the oral route following pre-treatment with sodium bicarbonate solution. Participants will be directly observed for 15 minutes following challenge and if they vomit within 60 minutes of challenge will be treated with antibiotics and withdrawn from the study. Following challenge administration participants will be seen daily for 14 days and instructed to complete an online challenge e-diary to record oral temperatures twice- daily and to describe any symptoms or the use of any medications for 28 days (supplementary material 5).

Paratyphoid infection will be diagnosed based on certain pre-specified criteria as seen in Table 2.

For peer review only - http://bmjopen.bmj.com/site/about/guidelines.xhtml

Table 2. Criteria for diagnosis of paratyphoid following human challenge

Paratyphoid fever is diagnosed if ANY of the following apply
A positive blood culture for *S.* Paratyphi A from 72 hours post-challenge
A positive blood culture for *S.* Paratyphi A within 72 hours post-challenge, with one or more signs/symptoms of paratyphoid infection (such as recorded temperature ≥38.0°C)
Persistent positive blood cultures (two or more blood cultures taken at least 4 hours apart) for *S.* Paratyphi A within 72 hours post-challenge.
Oral temperature ≥38.0°C persisting for 12 hours in the 14 days following challenge

Once diagnosed, participants are treated with antibiotics and monitored for severity of infection. Participants will be provided with medications for symptomatic control if required. At any stage, if clinically indicated, an additional review will be arranged by the clinical study team or if severely unwell they will be directed to the local Accident and Emergency department (or appropriate other medical facility) and relevant medical personnel from the trial and secondary care will be made aware. Antibiotics may also be started if felt to be clinically necessary or participant has severe symptoms not meeting diagnostic criteria.

Follow-up visits

Participants will be followed up for 365 days following challenge. To detect chronic carriage of *S*. Paratyphi A and to confirm clearance, all participants are required to produce three stool samples obtained a minimum of 48 hours apart produced at least one week after completion of the antibiotic course. Participants with three successive negative stool samples will be considered to be fully treated for *S.* Paratyphi A infection and no longer an infection risk. The participants GP and local Health Protection Unit will be notified following challenge with *S*. Paratyphi A and then again once the participant has completed their clearance samples.

Laboratory testing

Samples will be taken throughout the trial for safety, microbiological and immunological assays which are summarised in supplementary material 6.

Trial interventions

For peer review only - http://bmjopen.bmj.com/site/about/guidelines.xhtml

Vaccine

CVD 1902 is a live attenuated strain of *Salmonella* Paratyphi A, an unlicensed, experimental oral vaccine for *Salmonella* Paratyphi A infection developed by the Center for Vaccine Development at the University of Maryland School of Medicine in Baltimore. It contains two independently attenuating mutations, one in the *guaBA* locus, and the other in *clpPX*. *GuaBA* encodes genes involved in guanine biosynthesis and *clpPX* encodes a protease which targets the master flagellar regulator. The product is manufactured, tested and labelled according to current EMA guidelines in keeping with Good Manufacturing Practice (GMP) by Bharat Biotech International Limited. The strain is fully sensitive to ciprofloxacin, trimethoprim/sulfamethoxazole and ampicillin (19). This vaccine has been tested in adults in a phase 1 trial at the University of Maryland where a total of 30 healthy young adults received a single oral dose of CVD 1902 in a single-site, randomised, double-blinded phase 1 study (19,20).

In this trial, vaccine recipients will receive 2 doses of CVD 1902 delivered in 30 mL carrier sodium bicarbonate solution, 14 days apart. Each dose will contain not less than 2 x 10^{10} CFU, with an upper limit of 1.7 x10^{11} CFU

The lower limit of the dose range for the vaccine in this trial corresponds to the highest dose administered in the previous Phase 1 trial which was shown to be safe and immunogenic (20). As higher doses were required in the Phase 1 study to elicit immune responses, a two-dose schedule has been chosen to optimise immunogenicity whilst also testing a schedule that ultimately may be feasible at a population level. The rationale for using two doses of CVD 1902 14 days apart is to try and ensure an optimum immune responses and protection as a previous study by Darton et al 2016 showed that a single-dose live attenuated oral vaccine for *Salmonella* Typhi M01ZH09 trialled within in a human challenge model did not provide protection, despite being immunogenic (21).

Placebo

The vaccine placebo is 30ml 1.3% wt/vol sodium bicarbonate solution made up using BP sodium bicarbonate powder with sterile water.

Challenge

The *S*. Paratyphi A challenge strain, NVGH308, was isolated from a participant in a clinical study performed by the Oxford University Clinical Research Unit at Patan Hospital, Kathmandu, Nepal. It has been manufactured into batches to GMP standard by GenIbet BioPharmaceuticals, Portugal, and is supplied to the Oxford Vaccine Group by Novartis Vaccines for Global Health. The strain is fully sensitive to ciprofloxacin, azithromycin, ampicillin and trimethoprim/sulfamethoxazole.

For peer review only - http://bmjopen.bmj.com/site/about/guidelines.xhtml

Antibiotics

Antibiotics are used to treat diagnosed *Salmonella* Paratyphi A infection or at day 14 post challenge for those who have not been diagnosed.

The first line antibiotic will be oral ciprofloxacin 500mg twice daily for 7 days, in-line with guidance on enteric fever treatment (22). In fully susceptible S. Typhi isolates ciprofloxacin was shown to shorten time to resolution of symptoms, fever clearance time and bacteraemia when compared to azithromycin (23). Participants will be given verbal and written information regarding the possible side effects of ciprofloxacin use. For any participant in whom a contraindication to these first line antibiotics becomes apparent oral trimethoprim/sulfamethoxazole, oral azithromycin or oral amoxicillin will be used as second-line therapy.

Other trial interventions

Concomitant medication can be provided for symptomatic control of paratyphoid infection, before and after diagnosis. Concomitant medication after paratyphoid diagnosis includes antibiotics and antipyretics if required (supplementary material 7).

Sample size

Based on findings from the previous challenge studies performed at OVG, the assumption of attack rate of 58% was used in the control group (14,15). To demonstrate a protective effect of 70% (i.e. 30% relative risk in attacking rate), resulting in a reduction in attack rate from 58% in the control group to 17.4% in the vaccine group, 33 participants would be needed per group to achieve 90% power (1-β) at two-sided 5% significance level (α).

We observed less than a 10% of dropout rate from previous vaccine trial using typhoid challenge model (12). The sample size will be inflated to 37-38 participants per group to account for at least a 10%-dropout. We expect to randomise 74-76 participants in total.

Statistical analysis

The analysis of the primary end point will be a calculation of the proportion of participants diagnosed with paratyphoid fever in the CVD 1902 group relative to the placebo group. The proportion of participants diagnosed with paratyphoid fever (i.e., the attack rate) and the associated 95% confidence intervals will be presented by group at Day 28 after challenge. When calculating paratyphoid diagnosis proportions, the numerator will be the number of participants who meet the criteria for diagnosis and the denominator will be the per protocol population. The difference in proportions between the CVD 1902 and placebo groups will be analysed using Pearson's chi-squared test (or Fisher's Exact test if expected counts in any group are less than 5). To fulfil the primary objective, the protective effect of CVD 1902 over placebo will be calculated by:

$$PE = 100 \times (AR_{Placebo} – AR_{CVD\ 1902})/AR_{Placebo} = 100 \times (1 – AR_{CVD\ 1902}/ AR_{Placebo}),$$

Where PE is the protective effect and AR is the attack rate.

For peer review only - http://bmjopen.bmj.com/site/about/guidelines.xhtml

The 95%CI of the $AR_{CVD\ 1902}$/ $AR_{Placebo}$ can be calculated using standard methods for calculation of 95%CI on a rate ratio. The corresponding 95%CI for PE will also be calculated.

A secondary analysis of the primary endpoint will be conducted using the Kaplan-Meier method which will include all participants. Participants who withdrew or had potential interference with vaccine effect or infection challenge, (e.g. treated prior to Day 14 with no diagnosis of paratyphoid) will be censored in the analysis at the time of withdrawal or interference. Non-diagnosed participants will also be censored in the analysis at the time point of final monitoring of blood culture or temperature (Day 28).

Time-to-event analyses of individual components of the primary outcome (e.g. positive blood culture, oral temperature ≥38.0°C etc.) will be conducted using the Kaplan-Meier method and will include all participants. Participants not meeting the criteria for an individual component of the primary endpoint will be censored in the analysis at the final monitoring for those undiagnosed

Immunogenicity data are expected to be highly skewed and will be log-transformed prior to analysis. Results will be presented as geometric means with 95% confidence intervals. Values below the limit of detection (LLOD) will be imputed with half the value of the LLOD.

Ethics and dissemination

Ethical approval, regulation, and governance

Ethical approval for this study has been obtained from the Berkshire Research Ethics Committee and Health Research Authority (REC reference: 21/SC/0330) and the study will run in accordance with the ethical principles of the Declaration of Helsinki.

The use of an unlicensed vaccine in this study, CVD1902, is regulated by the Medicines for Human Use (Clinical Trials) Regulations 2004 (MHRA). As CVD 1902 is a genetically modified organism, (GMO), relevant approvals have been obtained for its use in this study from the Department of Environment, Food and Rural Affairs (DEFRA), Oxford University Hospitals NHS Foundation Trust (OUH) and the Centre for Clinical Vaccinology and Tropical Medicine (CCTVM). The challenge agent falls outside the remit of these regulators and therefore is judged according to common law and best practice (24). Regional ethics committees therefore provide support in governing challenge studies.

An independent Data and Safety Monitoring Committee (DSMC) consisting of an experienced group of infectious disease clinicians, scientists and a statistician will be appointed to provide real-time oversight of safety and trial conduct. The DSMC will review safety data collated from the eCRFs and e-diaries including solicited and unsolicited symptoms, laboratory results and vital signs. Following prime vaccination of a sentinel group of six participants relevant safety data will be reviewed by the DSMC. If there are no safety concerns these participants can proceed to their second vaccination and further participants can begin vaccination. Further DSMC reviews will occur regularly throughout the trial.

For peer review only

For peer review only - http://bmjopen.bmj.com/site/about/guidelines.xhtml

A development safety update report (DSUR) for the IMP will be prepared annually, on the anniversary of the MHRA approval for the trial.

Risks

The general risks to participants in this study are associated with potential side effects from the vaccine or placebo, symptomatic infection following challenge and the small risk of subsequent complications from paratyphoid infection. Additionally, there is a small risk of secondary transmission of *S.* Paratyphi A to close contacts of participants.

Vaccine

In the Phase 1 trial “CVD 1902 was well tolerated without clinically significant adverse reactions attributed to the vaccine” (19). Hypersensitivity to the vaccine is extremely unlikely given the constituents present in the vaccine preparation but there may be unforeseeable side effects. Participants will be asked to keep an e-diary of post-vaccination symptoms which will be checked daily by a study doctor in relation to the individual and trial stopping rules (supplementary material 8). Severe adverse events related to vaccination will undergo further evaluation by a clinician. Subjects will have the details of a 24-hour contact study doctor and can be seen for unscheduled visits as required.

Challenge

Some study participants will develop symptomatic paratyphoid infection following challenge, for which risks include development of severe disease or complications. These risks will be greatly minimised by daily reviews by a clinical study team member, daily review of e-diary entries, regular safety bloods and early treatment with an effective antimicrobial. A previous challenge study using the same strain of *S.* Paratyphi A at OVG has demonstrated a good safety profile (14).

Carriage

The likelihood of developing chronic carriage is extremely low in the challenge setting. Participants are treated with ciprofloxacin, a fluoroquinolone antibiotic which is the preferred class of antibiotics for prevention of chronic carriage (25). To ensure clearance of infection and to exclude chronic carriage, stool samples for culture will be obtained upon completion of the initial antibiotic course. If participants remain positive after a second course of antibiotic treatment (subsequent to the initial course) then participants will be referred to an Infectious Diseases Consultant at the Oxford University Hospitals NHS Foundation Trust for further management.

For peer review only

For peer review only - http://bmjopen.bmj.com/site/about/guidelines.xhtml

Close contacts

In view of the low infectivity of S. Paratyphi A without bicarbonate buffer and the high standard of hygiene and sanitation in the UK, secondary transmission of the challenge strain to household or other close contacts after discharge is highly unlikely. It is thought that typhoidal Salmonellas, unlike *Shigella sp.*, enterohaemorrhagic *Escherichia coli,* or hepatitis A virus, are virtually never transmitted by direct faecal-oral contact. This is in part due to the higher oral inoculum of these bacteria required to cause clinical disease.

It is acknowledged, however, that transmission within households can occur if the individual excreting *S.* Paratyphi A fails to practice effective hand washing after defecation and is subsequently involved in uncooked food preparation. If food is kept at ambient temperatures, bacterial proliferation occurs such that an infective dose level is reached, and the food then may act as a vehicle for paratyphoid transmission.

Throughout the period of possible excretion of the challenge strain, participants must practice stringent hand washing techniques after defecation. Participants will be given soap and paper towels for use at home and detailed advice on how to prevent transmission of *S.* Paratyphi A. Participants will be taught and observed practising good hygiene technique at their initial challenge visit. The importance of adhering to sanitation advice will be emphasised to participants.

Adverse event reporting

All adverse events occurring from first vaccination until 28 days after challenge will be recorded. Solicited and unsolicited adverse events following vaccination and challenge will be recorded and graded in electronic e-diaries. All solicited AEs will be automatically assumed to be related to vaccine/challenge whereas all unsolicited AEs will undergo causality assessment in relation to the vaccine, challenge agent or other medications, depending on when they are reported. Laboratory results and vital signs will also be severity graded and clinically significant results will be reported as adverse events.

All serious adverse events (SAEs) will be recorded from time of consent and reported to the DSMC within 24 hours of notification. All SAEs at least possibly related to CVD 1902 will be considered unexpected and be reported to the MHRA and REC as serious unexpected serious adverse reactions within the regulatory timelines. Adverse events of special interest (AESIs) are listed in table 3. These will be reported to the DSMC as soon as possible but within 7 days of discovery.

Table 3: Adverse events of special interest in the VASP trial

Adverse events of special interest
Severe paratyphoid infection*

For peer review only

For peer review only - http://bmjopen.bmj.com/site/about/guidelines.xhtml

Failure to clinically or bacteriologically cure a participant of paratyphoid infection
Progression to chronic carrier state
Relapse of paratyphoid infection
Transmission of S. Paratyphi A to a contact of a participant
AEs requiring a physician visit or Emergency Department visit which, in opinion of study staff, are related to challenge with S. Paratyphi A
Pregnancy

*Severe paratyphoid infection defined as illness that includes any of the following criteria: oral temperature > 40 degrees celcius, systolic blood pressure <85 mmHg, significant lethargy or confusion, gastrointestinal bleeding, gastrointestinal perforation or any grade 4 or above laboratory abnormality.

Dissemination

The trial protocol is registered on the ISRCTN registry (ISRCTN15485902). Dissemination of the findings is planned for publication in peer-reviewed journals and at scientific international conferences.

Patient and public involvement

The protocol, study information booklet and recruitment materials were reviewed by a local patient consultation group who provided feedback and comments on the initial documents. Their comments led to changes in the participant-facing documents, ensuring they are easy and clear for participants to understand.

Discussion

This study will be the first efficacy analysis of a live-attenuated oral vaccine against *S.* Paratyphi A using a human challenge model. Resulting data has the potential to be a critical step in the future licensure of the first vaccine against *S.* Paratyphi A. The role of a *S.* Paratyphi A vaccine is most likely to be part of a bi- or even multi-valent vaccine against both typhoidal and potentially non-typhoidal serovars. Deployment of a bivalent vaccine against S. Typhi and S. Paratyphi A would address the burden of enteric fever within an Asian context where both pathogens are endemic. Other areas, such as sub-Saharan Africa non-typhoidal serovars cause a significant burden of disease hence a multi-valent vaccine incorporating typhoidal and non-typhoidal serovars may prove more cost-effective as a single approach regardless of geographical setting. However, in order to contribute to this vaccine development, an efficacious S. Paratyphi A vaccine is required. This trial will act as a proof of concept efficacy trial for such a vaccine, CVD 1902.

Reliable immunologic correlates of protection for *S.* Typhi and *S.* Paratyphi A have not yet been identified. Progress has been made in *S.* Typhi with data from prior *S.* Typhi challenge trials identifying anti-Vi IgA and IgG1 antibodies correlated with protection following Vi-TT and Vi-PS

For peer review only

vaccination (26,27). However, *S.* Paratyphi A lacks the Vi antigen and it's immunobiology and pathogenesis is less well understood than it is for *S.* Typhi. The lipopolysaccharide O:2 has been identified as an important virulence factor for *S.* Paratyphi and the flagellar (H) antigens may also play a role in protection. During this trial we will evaluate both the humoral and cellular response to both CVD 1902 and *S.* Paratyphi challenge and in doing so provide invaluable immunological data and further our understanding of immunity to *S*. Paratyphi infection and correlates of protection.

As a controlled human infection model the conditions during this trial will differ from endemic areas. The population in this trial is healthy UK adults whereas the majority of paratyphoid infections in endemic areas occur in young children. Although different physiologically, these groups may be similar from an immunological perspective as they are both paratyphoid naïve. The stringent diagnostic criteria in this trial may also underestimate vaccine efficacy by picking up sub-clinical bacteraemia, whilst patients in endemic areas are more likely to be diagnosed on symptoms alone. Nevertheless, the results of the typhoid conjugate vaccine human challenge study showed that this model can be used to provide early, representative data on vaccine efficacy that does translate to efficacy in field studies (8,12).

Although no licensed vaccines against *S.* Paratyphi A exist there are several other mono and bivalent vaccines in development. Conjugate vaccines use the lipopolysaccharide O-antigen (O:2) of paratyphoid conjugated to a protein carrier, either to tetanus-toxoid or CRM_{197} a nontoxic mutant of diptheria toxin. Phase I/II studies of O:2-TT showed this vaccine was safe and immunogenic and further phase II trials are currently underway(28) with a bivalent formation combined with Vi-TT also in development. Pre-clinical studies demonstrated O:2-CRM_{197} was immunogenic when used alone or with Vi-CRM_{197} Novel vaccines, such as nanoparticle-based vaccines and MAPS-based vaccines are also in development (29).

As the first trial using a human challenge model to test efficacy of a S. Paratyphi vaccine, this trial paves the way for potential testing of other *S*. Paratyphi A vaccine candidates. Alongside improvements in sanitation and access to safe water supply, the development of an effective vaccine against *S*. Paratyphi A, has the potential to significantly impact on the burden of enteric fever in endemic countries.

Contributors

AJP, BA and MNR are the chief and principal investigators, who alongside MML, conceived and developed the study. NM and KE are research fellows who drafted the manuscript and protocol. XL, MG and KC are the study statisticians. FM, SC, EJ, JH, MV contributed to the laboratory aspects of the study design. NS and HR helped in design of the final study protocol and co-ordinated regulatory approvals. All authors read and approved the final manuscript.

For peer review only - http://bmjopen.bmj.com/site/about/guidelines.xhtml

Funding

This work was supported by Medical Research Council, grant number MR/RO25347/1.

Competing interests' statement

AJP is chair of the UK Department of Health and Social Care's (DHSC) Joint Committee on Vaccination and Immunisation (JCVI), was a member of WHOs Strategic Advisory Group of Experts until 2022, receives consulting fees from Shionogi and is in receipt of grants from MRC, AstraZeneca, Gates Foundation, Wellcome Trust, NIHR and European Commission through the University of Oxford. MML is a co-investigator on the MRC grant for this study, holds patents for Attenuated Salmonella Enterica Serovar Paratyphi A and Uses Thereof in the US and UK, Germany, France, Italy and Spain, is a member of the FDA Vaccines and Related Biological Products Advisory Committee (VRBPAC) and a member of the NIH Data and Safety Monitoring Board (DSMB) to review and monitor US-government-supported clinical trials of candidate COVID-19 vaccines (2020 to present). MNR works as a principal investigator on clinical vaccine trials funded by AstraZeneca and Moderna but receives no personal financial payment for this work. All other authors declare no competing interests.

References

1. Stanaway JD, Reiner RC, Blacker BF, Goldberg EM, Khalil IA, Troeger CE, et al. The global burden of typhoid and paratyphoid fevers: a systematic analysis for the Global Burden of Disease Study 2017. Lancet Infect Dis [Internet]. 2019 Apr 1 [cited 2021 Mar 17];19(4):369–81. Available from: http://dx.doi.org/10.1016/
2. Sood S, Kapil A, Dash N, Das BK, Goel V, Seth P. Paratyphoid fever in India: An emerging problem [3]. Emerging Infectious Diseases. 1999.
3. Kuijpers LMF, Veng CH, Sar D, Chung P, Phe T, Kham C, et al. Ongoing outbreak of Salmonella enterica serovar Paratyphi A infections, Phnom Penh, Cambodia. Journal of Infection in Developing Countries. 2015.
4. Tankhiwale SS, Agrawal G, Jalgaonkar S V. An unusually high occurrence of Salmonella enterica serotype Paratyphi A in patients with enteric fever. Indian J Med Res. 2003;
5. Herdman MT, Karo B, Dave J, Katwa P, Freedman J, Nascimento V Do, et al. Increasingly limited options for the treatment of enteric fever in travellers returning to England, 2014-2019: A cross-sectional analytical study. J Med Microbiol. 2021;
6. Typhoid and paratyphoid: laboratory confirmed cases in England, Wales and Northern Ireland - GOV.UK [Internet]. [cited 2022 May 4]. Available from: https://www.gov.uk/government/publications/typhoid-and-paratyphoid-laboratory-confirmed-cases-in-england-wales-and-northern-ireland

For peer review only - http://bmjopen.bmj.com/site/about/guidelines.xhtml

7. McCann N, Nabarro L, Morris-Jones S, Patel T, Godbole G, Heyderman R, et al. Outpatient management of uncomplicated enteric fever: A case series of 93 patients from the Hospital of Tropical Diseases, London. J Infect [Internet]. 2022 Jun [cited 2022 Aug 13];0(0). Available from: http://www.journalofinfection.com/article/S0163445322003814/fulltext

8. Shakya M, Voysey M, Theiss-Nyland K, Colin-Jones R, Pant D, Adhikari A, et al. Efficacy of typhoid conjugate vaccine in Nepal: final results of a phase 3, randomised, controlled trial. Lancet Glob Heal. 2021;

9. Patel PD, Patel P, Liang Y, Meiring JE, Misiri T, Mwakiseghile F, et al. Safety and Efficacy of a Typhoid Conjugate Vaccine in Malawian Children. N Engl J Med. 2021;

10. Qadri F, Khanam F, Liu X, Theiss-Nyland K, Biswas PK, Bhuiyan AI, et al. Protection by vaccination of children against typhoid fever with a Vi-tetanus toxoid conjugate vaccine in urban Bangladesh: a cluster-randomised trial. Lancet. 2021;

11. Dong B-Q, Yang J, Wang X-Y, Gong J, von Seidlein L, Wang M-L, et al. Trends and disease burden of enteric fever in Guangxi province, China, 1994–2004. Bull World Health Organ. 2010;

12. Jin C, Gibani MM, Moore M, Juel HB, Jones E, Meiring J, et al. Efficacy and immunogenicity of a Vi-tetanus toxoid conjugate vaccine in the prevention of typhoid fever using a controlled human infection model of Salmonella Typhi: a randomised controlled, phase 2b trial. Lancet. 2017;

13. Gibani MM, Jones E, Barton A, Jin C, Meek J, Camara S, et al. Investigation of the role of typhoid toxin in acute typhoid fever in a human challenge model. Nat Med. 2019;

14. H.C. D, M.M. G, C. J, H.B. T-B, M. V, T.C. D, et al. Evaluation of the clinical and microbiological response to salmonella paratyphi a infection in the first paratyphoid human challenge model. Clin Infect Dis. 2017;

15. Gibani MM, Jin C, Shrestha S, Moore M, Norman L, Voysey M, et al. Homologous and heterologous re-challenge with salmonella typhi and salmonella paratyphi a in a randomised controlled human infection model. PLoS Negl Trop Dis. 2020;

16. Martin LB, Simon R, MacLennan CA, Tennant SM, Sahastrabuddhe S, Khan MI. Status of paratyphoid fever vaccine research and development. Vaccine. 2016;

17. Shakya M, Neuzil KM, Pollard AJ. Prospects of Future Typhoid and Paratyphoid Vaccines in Endemic Countries. J Infect Dis [Internet]. 2021 Dec 20 [cited 2022 Mar 3];224(Supplement_7):S770–4. Available from: https://academic.oup.com/jid/article/224/Supplement_7/S770/6347515

18. Enteric fever (typhoid and paratyphoid. 2018 [cited 2022 Mar 3]; Available from: www.facebook.com/PublicHealthEngland

19. Center for Vaccine Development. Investigator's Brochure: Strain CVD 1902. Univ Maryl Sch Med. 2018;

20. Wahid R, Kotloff KL, Levine MM, Sztein MB. Cell mediated immune responses elicited in volunteers following immunization with candidate live oral Salmonella enterica serovar Paratyphi A attenuated vaccine strain CVD 1902. Clin Immunol. 2019;

For peer review only - http://bmjopen.bmj.com/site/about/guidelines.xhtml

21. Darton TC, Jones C, Blohmke CJ, Waddington CS, Zhou L, Peters A, et al. Using a Human Challenge Model of Infection to Measure Vaccine Efficacy: A Randomised, Controlled Trial Comparing the Typhoid Vaccines M01ZH09 with Placebo and Ty21a. PLoS Negl Trop Dis [Internet]. 2016 Aug 17 [cited 2022 Mar 3];10(8):e0004926. Available from: https://journals.plos.org/plosntds/article?id=10.1371/journal.pntd.0004926

22. Nabarro LE, McCann N, Herdman MT, Dugan C, Ladhani S, Patel D, et al. British infection association guidelines for the diagnosis and management of enteric fever in England. J Infect [Internet]. 2022 Jan [cited 2022 Feb 2];0(0). Available from: http://www.journalofinfection.com/article/S0163445322000135/fulltext

23. Jin C, Gibani MM, Pennington SH, Liu X, Ardrey A, Aljayyoussi G, et al. Treatment responses to azithromycin and ciprofloxacin in uncomplicated Salmonella Typhi infection: A comparison of clinical and microbiological data from a controlled human infection model. PLoS Negl Trop Dis. 2019;

24. Academy of Medical Sciences. Microbial challenge studies of human volunteers. Acad Med Sci London. 2005;

25. Ferreccio C, Morris JG, Valdivieso C, Prenzel I, Sotomayor V, Drusano GL, et al. Efficacy of ciprofloxacin in the treatment of chronic typhoid carriers. J Infect Dis. 1988;

26. Jin C, Hill J, Gunn BM, Yu WH, Dahora LC, Jones E, et al. Vi-specific serological correlates of protection for typhoid fever. J Exp Med. 2020;

27. Dahora LC, Jin C, Spreng RL, Feely F, Mathura R, Seaton KE, et al. IgA and IgG1 Specific to Vi Polysaccharide of Salmonella Typhi Correlate With Protection Status in a Typhoid Fever Controlled Human Infection Model. Front Immunol. 2019;

28. Konadu E, Shiloach J, Bryla DA, Robbins JB, Szu SC. Synthesis, characterization, and immunological properties in mice of conjugates composed of detoxified lipopolysaccharide of Salmonella paratyphi A bound to tetanus toxoid, with emphasis on the role of O acetyls. Infect Immun. 1996;

29. Immunization, Vaccines and Biologicals [Internet]. [cited 2022 Mar 11]. Available from: https://www.who.int/teams/immunization-vaccines-and-biologicals/diseases/paratyphoid-fever

For peer review only

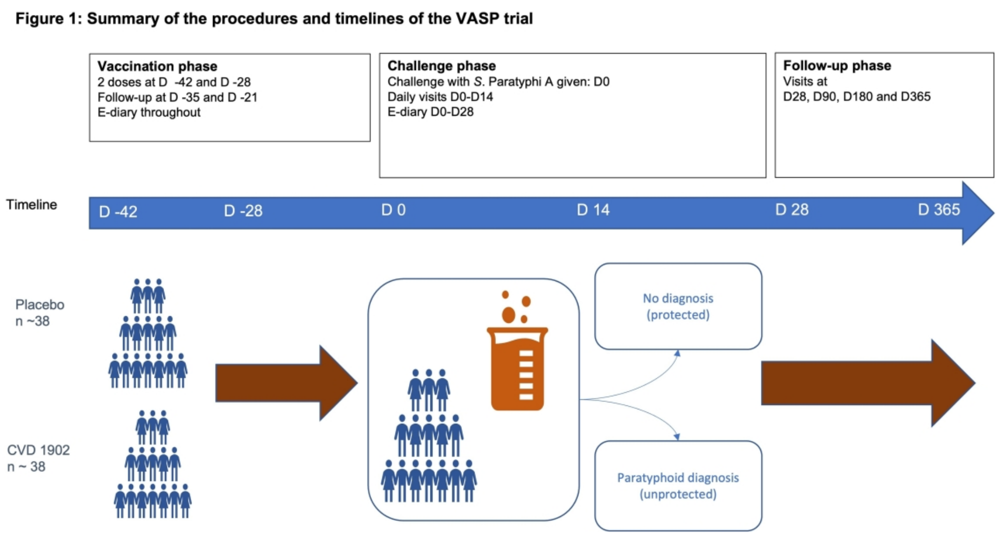

Figure 1: Summary of the procedures and timelines of the VASP Trial

309x163mm (300 x 300 DPI)

Supplementary Material 1: Inclusion and Exclusion Criteria

Inclusion Criteria

Participants must satisfy all the following criteria to be considered eligible for the study:

- Willing and able to give informed consent for participation in the study.
- Aged between 18 and 55 years inclusive at time of vaccination.
- In good health as determined by medical history, physical examination and clinical judgment of the study team.
- Willing to be available in Oxford for all required appointments
- Agree (in the study team's opinion) to comply with all study requirements, including capacity to adhere to good personal hygiene and infection control precautions.
- Agree to allow study staff to contact his or her GP to access the participant's vaccination records, medical history and have their opinion solicited as to the participant's appropriateness for inclusion.
- Agree to allow study staff to access NHS health records as required for study purposes.
- Agree to allow his or her GP (and/or Consultant if appropriate), to be notified of participation in the study.
- Agree to allow UKHSA to be informed of their participation in the study.
- Agree to give his or her close household contacts written information informing them of the participants' involvement in the study and offering them voluntary screening for *S.* Paratyphi A carriage.
- Agree to have 24-hour contact with study staff during the four weeks post challenge and are able to ensure that they are contactable by mobile phone for the duration of the vaccination and challenge period until antibiotic completion.
- Have internet access to allow completion of the e-diary and real-time safety monitoring.
- Agree to avoid antipyretic/anti-inflammatory treatment from challenge until advised by a study doctor or until 14 days after challenge.
- Agree to refrain from donating blood for the duration of the study.
- Agree to provide their National Insurance/Passport number for the purposes of TOPS registration and for payment of reimbursement expenses.
- Participants must have received at least one dose of a SARS-CoV-2 vaccine that has been approved for use by the MHRA (or other national regulatory authority) $\geq$ four weeks prior to enrollment.

For peer review only

- Agree to not receive other vaccinations (eg Covid-19 vaccines) during the 7 days before and after study vaccination and during the 7 days before or 21 days post-challenge.

1.1. Exclusion Criteria

The participant will not be enrolled if any of the following apply:

- History of significant organ/system disease that could interfere with trial conduct or completion. Including, for example, but not restricted to:
    - Cardiovascular disease including a diagnosis of hypertension
    - Respiratory disease
    - Haematological disease[1]
    - Endocrine disorders
    - Renal or bladder disease, including history of renal calculi
    - Biliary tract disease, including biliary colic, asymptomatic gallstones or previous cholecystectomy
    - Gastro-intestinal disease including requirement for antacids, H_2-receptor antagonists, proton pump inhibitors or laxatives
    - Neurological disease
    - Metabolic disease
    - Autoimmune disease
    - Psychiatric illness requiring hospitalisation
    - Known or suspected drug misuse
    - Known or suspected alcohol misuse (alcohol misuse defined as an intake exceeding 42 units per week)
    - Infectious disease
- Have any known or suspected impairment of immune function, alteration of immune function, or prior immune exposure that may alter immune function to paratyphoid resulting from, for example:
    - Congenital or acquired immunodeficiency, including IgA deficiency
    - Human Immunodeficiency Virus infection or symptoms/signs suggestive of an HIV-associated condition

[1] This includes anaemia. The acceptable lower limits for [haemoglobin] are 125 g/L for female participants and 135 g/L for male participants (Guidelines for the Blood Transfusion Services in the UK, 8th edition, 2018 <https://www.transfusionguidelines.org/red-book> Accessed 5th December 2018).

  - Receipt of immunosuppressive therapy such as anti-cancer chemotherapy or radiation therapy within the preceding 12 months or long-term systemic corticosteroid therapy.
  - Receipt of immunoglobulin or any blood product transfusion within 3 months of study start.
  - History of cancer (except squamous cell or basal cell carcinoma of the skin and cervical carcinoma in situ).
- HLA-B27 positive.
- Moderate or severe depression or anxiety as classified by the Hospital Anxiety and Depression Score at screening or challenge that is deemed clinically significant by the study doctors[2].
- Weight less than 50 kg.
- Presence of implants or prosthetic material.
- Anyone taking long-term medication (e.g. analgesia, anti-inflammatories or antibiotics) that may affect symptom reporting or interpretation of the study results.
- Contraindication to fluoroquinolones, macrolide antibiotics, co-trimoxazole or ceftriaxone.
- Family history of aneurysmal disease
- Female participants who are pregnant, lactating or who are unwilling to ensure that they or their partner use effective contraception[3] 30 days prior to vaccination and continue to do so until three negative stool samples have been obtained after completion of antibiotic treatment.
- Full-time, part-time or voluntary occupations involving:
  - Clinical or social work with direct contact with young children (defined as those attending pre-school groups or nursery or aged under 2 years), or
  - Clinical or social work with direct contact with highly susceptible patients or persons in whom typhoid infection would have particularly serious consequences
  - Commercial food handling (involving preparing or serving unwrapped foods not subjected to further heating)

(unless willing to avoid work from vaccination until demonstrated not to be infected with *S.* Paratyphi A after challenge by clearance samples in accordance with guidance from UKHSA and willing to allow study staff to inform their employer).

---

[2] If elevated scores are due to temporary significant life events, the questionnaire may be repeated after resolution of the event with a view to inclusion if normal.

[3] As defined by CTFG Recommendations related to contraception and pregnancy testing in clinical trials, current document: https://www.hma.eu/fileadmin/dateien/Human_Medicines/01-About_HMA/Working_Groups/CTFG/2014_09_HMA_CTFG_Contraception.pdf [accessed 23rd July 2019]

For peer review only

- Close household contact with:
  - Young children (defined as those attending pre-school groups, nursery or those aged less than 2 years)
  - Individuals who are immunocompromised (including pregnancy).
- Scheduled elective surgery or other procedures requiring general anaesthesia during the study period.
- Participants who have participated in another research study involving an investigational product that might affect risk of paratyphoid infection or compromise the integrity of the study within the 30 days prior to enrolment (e.g. significant volumes of blood already taken in previous study)[4].
- Detection of any abnormal results from screening investigations (at the clinical discretion of the study team).
- Inability to comply with any of the study requirements (at the discretion of the study staff and the participant's General Practitioner).
- Any other social, psychological or health issues which, in the opinion of the study staff, may
  - put the participant or their contacts at risk because of participation in the study,
  - adversely affect the interpretation of the primary endpoint data,
  - Impair the participant's ability to participate in the study.
- Have any history of allergy to vaccine/placebo components
- Having been resident in an enteric fever endemic country for 6 months or more.
- Have previously been diagnosed with laboratory-confirmed typhoid or paratyphoid infection or been given a diagnosis compatible with enteric fever.
- Have participated in previous typhoid or paratyphoid challenge studies (with ingestion of challenge agent).
- Have received any oral typhoid vaccination (e.g. Ty21a or M01ZH09) at any time.
- Have a prolonged corrected QT interval (>450 milliseconds) on ECG screening.
- Significant blood donation or planned blood donation prior to enrolment.

[4] As assessed by both participant questioning and registration with The Over Volunteering Prevention System (TOPS) database.

For peer review only

Supplementary Material 2: Temporary Exclusion Criteria

Temporary Exclusion at Vaccination

Participants will be temporarily excluded from receiving vaccination if presenting at a vaccination visit with the following:

- Acute gastrointestinal illness within 24-hours prior to vaccination.
- Significant infection within the previous 7 days.
- Participant has experienced fever (>37.5°C) or subjective febrile symptoms within the previous 3 days (even with a negative COVID-19 test).
- Symptoms of COVID-19, or confirmed infection (according to government guidelines) within 14 days prior to vaccination visit; as per guidance in Section 9.11
- Validated positive SARS-CoV-2 test (NAAT or antigen) within 2 weeks prior to vaccination visit
- History of any antibiotic therapy during the previous 5 days.
- Any systemic corticosteroid (or equivalent) treatment in the previous 14 days, or for more than seven consecutive days within the past 3 months).
- Receipt of another enteric live vaccine within 4 weeks prior to vaccination or an injected live or killed vaccine within 7 days prior to vaccination.
- Plan to receive any vaccine other than the study vaccine within 7 days following vaccination.
- Therapy with antacids, proton pump inhibitors or H_2-receptor antagonists within 24 hours prior to vaccination.
- Unavailable for post-vaccination visits, second vaccination visit, and challenge visit as outlined in study procedures table (see section 7.4 table 1b).

If this is the first vaccine to be received and the temporary exclusion does not result in the participant becoming ineligible, then this vaccine visit can be rescheduled.

If this is the second vaccine to be received and the temporary exclusion does not result in the participant becoming ineligible, then this should be discussed with a study doctor as to whether they can be rescheduled for their second vaccination (see table 2, section 7.5 for visit windows) or withdrawn.

Temporary Exclusion at Challenge

For peer review only

Participants will be temporarily excluded from challenge if presenting at the challenge visit with the following. Participants can be challenged up to 28 days after their original challenge date (see table 2, section 7.5)

- Significant acute or acute-on-chronic infection within the previous 7 days or have experienced fever (>37.5□C) or subjective febrile symptoms within the previous 3 days (even with a negative COVID-19 test).
- Symptoms of COVID-19, or confirmed infection (according to government guidelines) within 14 days prior to challenge visit; as per guidance in Section 9.11
- Validated positive SARS-CoV-2 test (NAAT or antigen) within 2 weeks prior to challenge visit
- History of any antibiotic therapy during the previous 5 days.
- Any systemic corticosteroid (or equivalent) treatment in the previous 14 days, or for more than seven consecutive days within the past 3 months.
- Therapy with antacids, proton pump inhibitors or H_2-receptor antagonists within 24 hours prior to challenge.
- Participant has not received two doses of the study vaccine/placebo.
- Anaemia felt to be clinically significant by the study team.
- Receipt of a COVID-19 vaccine in preceding 7 days
- Plan to receive any vaccine within 21 days following challenge.

For peer review only

Supplementary Material 3: Visit Structure

Figure 1: Visit Structure of Trial

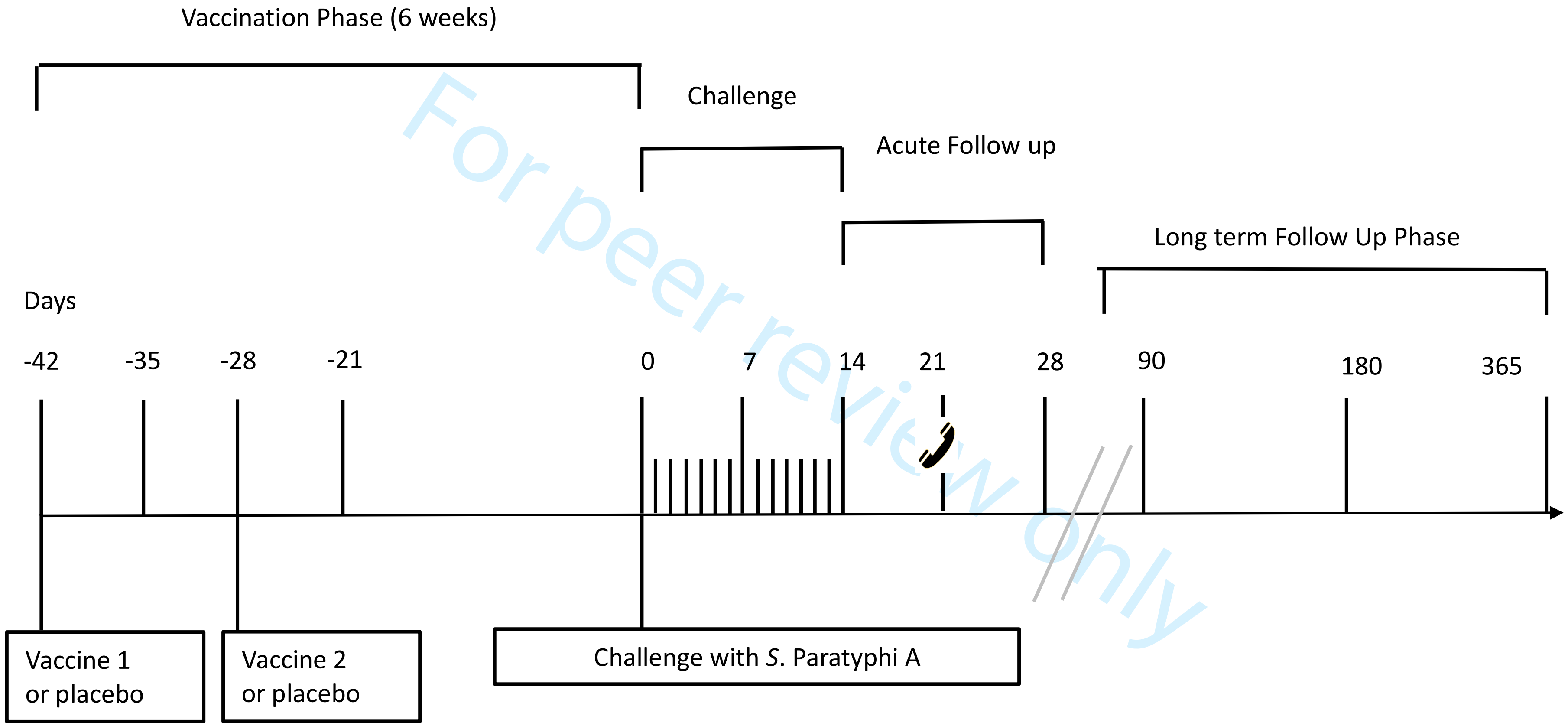

For peer review only

Figure 2: Structure of PD visits

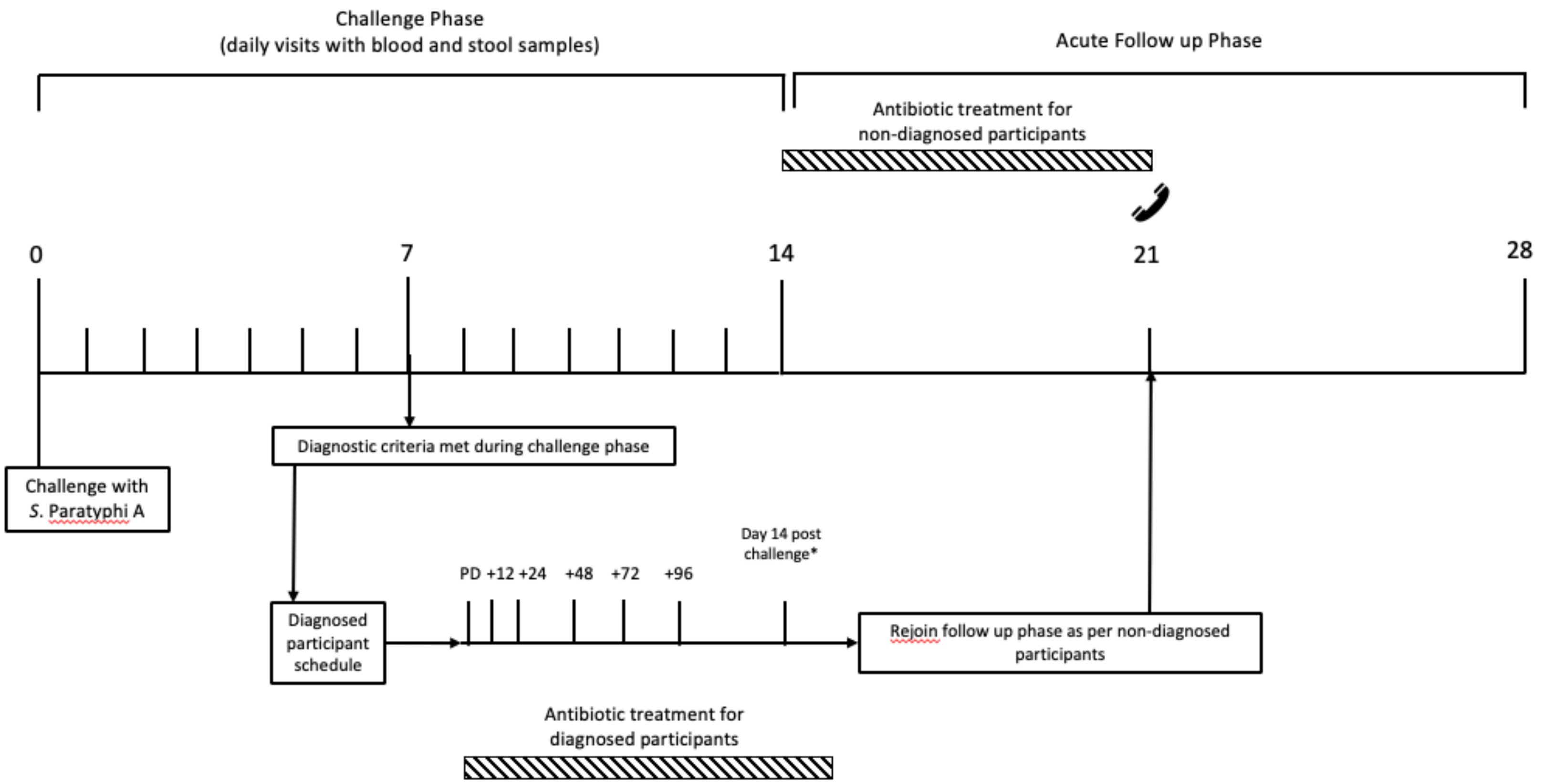

Supplementary Material 4: Vaccination e-diary

Sample of Daily Vaccination e-diary

Remember to measure your temperature **twice a day** following vaccination

	Day of First Vaccine	
Date		
AM/PM	AM	PM
Time		
Oral (mouth) temperature	°C	°C

**Symptom**	**Severity**				
Headache	0	1	2	3	4
Generally unwell	0	1	2	3	4
Loss of appetite	0	1	2	3	4
Abdominal/stomach pain	0	1	2	3	4
Nausea or vomiting	0	1	2	3	4
Diarrhoea	0	1	2	3	4

For peer review only

If you have not experienced a symptom please circle "0" on your diary page for the relevant day. If you have experienced a symptom please circle the number indicating the severity of the symptom using the scale.

Symptom	Severity			
	1	2	3	4
Headache	Present but no interference with activity	Some interference with activity	Significant; prevents daily activity	Emergency department visit or hospitalisation
Generally unwell	Present but no interference with activity	Some interference with activity	Significant; prevents dailyactivity	Emergency department visit or hospitalisation
Eating less than usual or loss of appetite	Eat less than normal for 1-2 meals	Miss 1-2 meals completely	Miss all meals	Emergency department visit or hospitalisation
Abdominal/ stomach pain	Present but no interference with activity	Some interference with activity	Significant; any use of codeine phosphate or prevents daily activity	Emergency department visit or hospitalisation
Nausea/vomiting	Present but no interference with activity or 1 – 2 episodes in 24 hours	Some interference with activity or more than 2 episodes in 24 hours	Significant; Prevents daily activity	Emergency department visit or hospitalisation
Diarrhoea	3-4 loose stools in 24 hrs	5-6 loose stools in 24 hrs	7 or more loose stools in 24 hrs	Emergency department visit or hospitalisation

For peer review only

If you feel feverish at any other time please check your temperature and record below:

Date								
Day (post vaccine)								
Time								
Oral (mouth) temperature	ºC	ºC	ºC	ºC	ºC	ºC	ºC	ºC

Date								
Day (post vaccine)								
Time								
Oral (mouth) temperature	ºC	ºC	ºC	ºC	ºC	ºC	ºC	ºC

For peer review only

Additional symptoms

perience other symptoms such as feeling hot or cold, sweating, shivering or constipat able to cope with any of the symptoms you are experiencing, please contact the study y.

d any additional symptoms on the following tables. Please include any illness/injury ev s not related to the vaccine. Please include the start date, start time, finish date, finish going if not resolved by day 28), and severity of symptoms (see below scale).

e any symptoms using the following scale:

Mild – easily tolerated with no limitation on normal activity

Moderate – some limitation of daily activity

Severe – unable to perform normal daily activity

Emergency department or hospital visit required

For peer review only

Start te/time	Symptom details	Severity (please circle)	Finish date/tin (state ongoing if con
/ __ / __<br>__ : __		1 2 3 4	__ / __ / __<br>__ : __
/ __ / __<br>__ : __		1 2 3 4	__ / __ / __<br>__ : __
/ __ / __<br>__ : __		1 2 3 4	__ / __ / __<br>__ : __
/ __ / __<br>__ : __		1 2 3 4	__ / __ / __<br>__ : __
/ __ / __<br>__ : __		1 2 3 4	__ / __ / __<br>__ : __
/ __ / __<br>__ : __		1 2 3 4	__ / __ / __<br>__ : __

v only

Date	(24 hr clock)	Medication	Dose	Reason

For peer review only

For peer review only

Remember to measure your temperature **twice a day** following challenge

Day 0

	Day 0	
Date		
AM/PM	AM	PM
Time		
Oral (mouth) temperature	°C	°C

**Symptom**	**Severity**				
Headache	0	1	2	3	4
Generally unwell	0	1	2	3	4
Loss of appetite	0	1	2	3	4
Abdominal/stomach pain	0	1	2	3	4
Nausea or vomiting	0	1	2	3	4
Muscle pain	0	1	2	3	4
Joint pain	0	1	2	3	4
Cough	0	1	2	3	4
Diarrhoea	0	1	2	3	4
Constipation	0	1	2	3	4
Rash	YES/ NO delete as appropria te	Describe where, colour, size (mm):			

If you have not experienced a symptom please circle "0" on your diary page for the relevant day. If you have experienced a symptom please circle the number indicating the severity of the symptom using the scale.

Symptom	Severity			
	**1**	**2**	**3**	**4**
Headache	Present but no interference with activity	Some interference with activity	Significant; any use of codeine phosphate or prevents daily activity	Emergency department visit or hospitalisation
Generally unwell	Present but no interference with activity	Some interference with activity	Significant; prevents daily activity	Emergency department visit or hospitalisation
Eating less than usual or loss of appetite	Eat less than normal for 1-2 meals	Miss 1-2 meals completely	Miss all meals	Emergency department visit or hospitalisation
Abdominal/ stomach pain	Present but no interference with activity	Some interference with activity	Significant; any use of codeine phosphate or prevents daily activity	Emergency department visit or hospitalisation
Nausea/ vomiting	Present but no interference with activity or 1 – 2 episodes in 24 hours	Some interference with activity or more than 2 episodes in 24 hours	Significant; Prevents daily activity	Emergency department visit or hospitalisation

Symptom	Severity			
	**1**	**2**	**3**	**4**
Muscle pain	Present but no interference with activity	Some interference with activity	Significant; prevents daily activity	Emergency department visit or hospitalisation
Joint pain	Present but no interference with activity	Some interference with activity	Significant; prevents daily activity	Emergency department visit or hospitalisation

For peer review only

Cough	Present but no interference with activity	Some interference with activity	Significant; prevents daily activity	Emergency department visit or hospitalisation
Diarrhoea	3-4 loose stools in 24 hrs (loose stool is defined as ≥200ml volume)	5-6 loose stools in 24 hrs (loose stool is defined as ≥200ml volume)	7 or more loose stools in 24 hrs (loose stool is defined as ≥200ml volume)	Emergency department visit or hospitalisation
Constipation	Present but no interference with activity	Some interference with activity	Significant; prevents daily activity	Emergency department visit or hospitalisation

For peer review only

If you feel feverish at any other time please check your temperature and record below:

Date								
Day (post challenge)								
Time								
Oral (mouth) temperature	°C	°C	°C	°C	°C	°C	°C	°C

Date								
Day (post challenge)								
Time								
Oral (mouth) temperature	°C	°C	°C	°C	°C	°C	°C	°C

For peer review only

Additional Symptoms

For peer review only

You may experience other symptoms such as feeling hot or cold, sweating or shivering. If you feel unable to manage any of the symptoms you are experiencing, please contact the study team immediately.

Please record any additional symptoms on the following tables. Please include any illness/injury even if you think it is not related to the vaccine or paratyphoid challenge. Please include the start date, start time, finish date, finish time (or state ongoing if not resolved by day 28), and severity of symptoms (see below scale).

Please grade any symptoms using the following scale:

- 1 Mild – easily tolerated with no limitation on normal activity
- 2 Moderate – some limitation of daily activity
- 3 Severe – unable to perform normal daily activity
- 4 Emergency department or hospital visit required

**Start Date/time**	**Symptom details**	**Severity** (please circle)	**Finish date/time** (state ongoing if continuing)
__/__/__ __:__		1 2 3 4	__/__/__ __:__
__/__/__ __:__		1 2 3 4	__/__/__ __:__
__/__/__ __:__		1 2 3 4	__/__/__ __:__
__/__/__ __:__		1 2 3 4	__/__/__ __:__
__/__/__ __:__		1 2 3 4	__/__/__ __:__
__/__/__ __:__		1 2 3 4	__/__/__ __:__

For peer review only

Medications

Please record any medication taken in addition to any regular medication that you take. Please do not tal
agreed by the study team.

Date	Time (24 hr clock)	Medication	Dose	Reason

For peer review only

For peer review only

Supplementary Material 6: Sample testing during VASP Trial

Table 1: Sample collection - screening

Screening		
	Specimen	Container
FBC, ESR	Blood	EDTA vacutainer
Urea, Electrolytes & Creatinine	Blood	Heparin vacutainer
HIV, HBsAg, HCVA	Blood	Serum vacutainer
TTG and IgA	Blood	Serum vacutainer
HLA B27	Blood	EDTA vacutainer
Total blood volume		Maximum 17 mL
Blood glucose	Blood	NA
Urine dipstick	Urine	Standard urine pot
Urine Pregnancy test	Urine	Standard urine pot

For peer re

Table 2: Sample collection – vaccination and post vaccination

Vaccine	Investigation	Pregnancy Test	Saliva	Stool sample	Blood culture	Full blood count	CRP, U+Es, LFTs	Blood for OVG laboratories	
	Sample tube	std urine pot	saliva coll-ection device	Stool Pot	Aerobic BACTEC bottle	EDTA Vacutainer	Heparin Vacutainer		
	**Volume blood (mL)**				10	1 → 3	2		**TOTAL***
	**Day**								
1	**D-42 (Va)**	x	x	x		2	3	74.5	79.5
	**D-35 (Vb)**			x	10	2	3	32.5	47.5
2	**D-28 (Vc)**	x	x	x	10	2	3	71	86
	**D-21 (Vd)**			x	10	2	3	32.5	47.5
								Total	260.5

Specific volumes and sample tubes for different assays for the OVG laboratories will be detailed in the Laboratory Analysis Plan and Clinical Study Plan.

Table 3: Sample collection – challenge period

Challenge	Investigation	SARS-CoV-2 Test	Pregnancy Test	Saliva	Stool sample	Blood culture	Full blood count	CRP, U+Es, LFTs	Blood for OVG laboratories	
	Sample tube	NP swab	std urine pot	saliva coll-ection device	Stool Pot	Aerobic BACTEC bottle	EDTA Vacutainer	Heparin Vacutainer		
	**D-2**	x								
→	**D0**		x	x	x	10	2	3	81	96
	**D1**				x	10			20	30
	**D2**				x	10	2	3	up to 4	19
	**D3**				x	10			0	10
	**D4**				x	10	2	3	up to 4	19
	**D5**				x	10			0	10
	**D6**				x	10	2	3	0	15
	**D7**				x	10	2		58.5	70.5
	**D8**				x	10	2	3	0	15
	**D9**				x	10			0	10
	**D10**				x	10	2	3	0	15
	**D11**				x	10			0	10
	**D12**				x	10	2	3	0	15
	**D13**				x	10			0	10
	**D14**	x	x		x	10	2	3	58.5	73.5
									Total*	418

Specific volumes and sample tubes for different assays for the OVG laboratories will be detailed in the Laboratory Analysis Plan and Clinical Study Plan.

For peer review only

Table 4: Sample collection – paratyphoid diagnosis, follow up and totals

Investigation	SARS-CoV-2 Test	Pregnancy Test	Saliva	Stool sample	Blood culture	Full Blood count	CRP, U+Es, LFTs	Blood for OVG laboratories	
Sample tube	NP swab	std urine pot	saliva coll-ection device	Stool Pot	Aerobic BACTEC bottle	EDTA Vacutain er	Heparin Vacutain er		
**Day**									**TOTAL**
**PD**	x	x		x	10	2	3	12.5	27.5
**PD +12hrs**				x	10	2		50	62
**PD +24hrs**				x	10	2	3	**	15
**PD +48hrs**				x	10	2	3	0	15
**PD +72hrs**				x	10	2	3	0	15
**PD +96hrs**				x	10	2	3	50	65
**D14PD gp**				x		2	3	0	5
**D28**			x	x		2	3	58.5	63.5
								Total	268
	**Maximum total for first 3 months (excluding screening)**			**If Paratyphoid NOT diagnosed**				**Total**	**738**
	**Maximum total for first 3 months (excluding screening)**			**If Paratyphoid Diagnosed (maximum blood volume)**				**Total**	**937.5**
**D90**			x	x		2	3	58.5	63.5
**D180**			x	x		2	3	48.5	53.5
**D365**			x	x		2	3	58.5	63.5
	**Maximum total in 14 months (excluding screening)**			**If Paratyphoid NOT diagnosed**				**Total**	**922.5**
	**Maximum total in 14 months (excluding screening)**			**If Paratyphoid Diagnosed (maximum blood volume)**				**Total**	**1122**

Specific volumes and sample tubes for different assays for the OVG laboratories will be detailed in the Laboratory Analysis Plan and Clinical Study Plan.

For peer review only - http://bmjopen.bmj.com/site/about/guidelines.xhtml

Supplementary Material 7: Medications used in the VASP trial

Table 1: Concomitant medications used

Drug	Indication	Dose	Route	Frequency
Paracetamol	Fever and discomfort (**after antibiotic therapy started**)	500mg - 1 Gram	Oral	PRN, max QDS
Codeine	Pain including headache	15-60mg	Oral	PRN (max. 240mg/24 hours)
Senna	Constipation	2-4 tablets	Oral	PRN
Cyclizine	Nausea and/or vomiting	50mg	Oral	PRN (max. 150mg/24 hours)
Chlorpheniramine	Allergy	4mg	Oral	PRN TDS-QDS (max. 24mg/24 hours)
Oral rehydration salts	Dehydration, vomiting or diarrhoea	1-2 sachets	Oral	PRN
Sando-K	Hypokalaemia	2-4 tablets	Oral	PRN, up to TDS dependent on potassium deficit

Table 2: Antibiotics used

Drug	Dose	Route	Frequency	Duration
Ciprofloxacin	500mg	Oral	BD	7 days
Co-trimoxazole	160/800mg	Oral	BD	7 days
Azithromycin	1g stat followed by 500mg	Oral	OD	7 days
Amoxicillin	500mg	Oral	TDS	7 days

For peer review only

For peer review only

Supplementary Material 8: Trial Stopping Rules

Safety holding Rules
In the event of any of the following, vaccination of further individuals will be paused pending DSMC review:

- New scientific information is published to indicate that subjects in the trial are being exposed to undue risks as a result of administration of the IMP, or as a result of the trial procedures or follow-up schedule.
- Bacteraemia with *Salmonella* Paratyphi A of any participant during the vaccination period
- Serious concerns about the safety of the IMP arise as a result of one or more vaccine related SAE(s) occurring in the subjects enrolled
- If at least two subjects develop a 'severe' adverse event related to the study drug, as assessed by a clinician, independent of within or not within the same-organ-class.

The DSMC chair will then undertake a review of the data to decide whether a temporary halt is required as an urgent safety measure and what the scope of the halt will be. A full meeting of the DSMC can be called at the chair's discretion. If, following DSMC review, a halt is required, the Sponsor, REC and appropriate regulatory authorities will be notified within 3 days and a substantial amendment submitted within 15 days. If the DSMC decide that a halt is not required then the trial may continue.

Following a halt to the trial, if it is decided that the trial may re-start, then a request and substantial amendment will be made to the Sponsor, the REC and the MHRA in order to do so, otherwise the trial will be terminated.

Individual stopping Rules
Stopping rules for individual volunteers will apply (i.e., indications to withdraw individuals from further vaccinations). Study participants who present with at least one of the following stopping rules will trigger a clinical review as to whether the participant should be withdrawn from further vaccination in the study, would not undergo challenge and would be followed up only from a safety perspective if participants consent to this.

- Laboratory Aes: the participant develops a confirmed $\geq$ grade 3 laboratory AE considered possibly, probably or definitely related within 7 days after vaccination
- Solicited adverse events: the participant develops a $\geq$ grade 3 systemic solicited AE considered possibly, probably or definitely related within 2 days after vaccination (day of vaccination and one subsequent day) which is deemed severe by clinician assessment
- Unsolicited adverse events: the participant has a $\geq$ grade 3 adverse event, considered possibly, probably or definitely related to vaccination which is deemed severe by clinician assessment or has a SAE considered possibly, probably or definitely related to vaccination.

For peer review only

- The participant has an acute allergic reaction or anaphylactic shock following the administration of the vaccine investigational product.

For peer review only

BMJ Open

Accelerating Clinical Development of a Live Attenuated Vaccine against Salmonella Paratyphi A (VASP): Study protocol for an observer-participant-blind randomised control trial of a novel oral vaccine using a human challenge model of Salmonella Paratyphi A infection in healthy adult volunteers

Journal:	*BMJ Open*
Manuscript ID	bmjopen-2022-068966.R1
Article Type:	Protocol
Date Submitted by the Author:	12-Apr-2023

Complete List of Authors:	McCann, Naina; University of Oxford Oxford Vaccine Group, Department of Paediatrics; NIHR Oxford Biomedical Research Centre<br>Emary, Katherine; University of Oxford Oxford Vaccine Group, Department of Paediatrics; NIHR Oxford Biomedical Research Centre<br>Singh, Nisha; University of Oxford Oxford Vaccine Group, Department of Paediatrics; NIHR Oxford Biomedical Research Centre<br>Mclean, Florence; University of Oxford Oxford Vaccine Group, Department of Paediatrics; NIHR Oxford Biomedical Research Centre<br>Camara, Susana; University of Oxford, Oxford Vaccine Group, Department of Paediatrics; NIHR Oxford Biomedical Research Centre<br>Jones, Elizabeth; University of Oxford Oxford Vaccine Group, Department of Paediatrics; NIHR Oxford Biomedical Research Centre<br>Kim, Young Chan; University of Oxford Oxford Vaccine Group, Department of Paediatrics; NIHR Oxford Biomedical Research Centre<br>Liu , Xinxue ; University of Oxford Oxford Vaccine Group, Department of Paediatrics ; NIHR Oxford Biomedical Research Centre<br>Greenland, Melanie ; University of Oxford Oxford Vaccine Group, Department of Paediatrics ; NIHR Oxford Biomedical Research Centre<br>Conlin, Kerry; University of Oxford Oxford Vaccine Group, Department of Paediatrics; NIHR Oxford Biomedical Research Centre<br>Hill, Jennifer; University of Oxford Oxford Vaccine Group, Department of Paediatrics ; NIHR Oxford Biomedical Research Centre<br>Verheul, Marije; University of Oxford Oxford Vaccine Group, Department of Paediatrics; NIHR Oxford Biomedical Research Centre<br>Robinson, Hannah; University of Oxford Oxford Vaccine Group, Department of Paediatrics; NIHR Oxford Biomedical Research Centre<br>Angus, Brian; University of Oxford Oxford Vaccine Group, Department of Paediatrics ; NIHR Oxford Biomedical Research Centre<br>Ramasamy, Maheshi ; University of Oxford Oxford Vaccine Group, Department of Paediatrics ; NIHR Oxford Biomedical Research Centre<br>Levine, Myron M ; University of Maryland School of Medicine, Center for Vaccine Development and Global Health<br>Pollard, Andrew; University of Oxford Oxford Vaccine Group, Department of Paediatrics ; NIHR Oxford Biomedical Research Centre

**Primary Subject Heading**:	Infectious diseases
Secondary Subject Heading:	Immunology (including allergy), Paediatrics
Keywords:	INFECTIOUS DISEASES, MICROBIOLOGY, Clinical trials < THERAPEUTICS

BMJ Open Access Protocol

Title: Accelerating Clinical Development of a Live Attenuated Vaccine against *Salmonella* Paratyphi A (VASP): Study protocol for an observer-participant-blind randomised control trial of a novel oral vaccine using a human challenge model of *Salmonella* Paratyphi A infection in healthy adult volunteers

Authors

Naina McCann [1,2]

Katherine Emary [1,2]

Nisha Singh [1,2]

Florence Mclean [1,2]

Susana Camara [1,2]

Elizabeth Jones [1,2]

Young Chan Kim [1,2]

Xinxue Li [1,2]

Melanie Greenland [1,2]

Kerry Conlin [1,2]

Jennifer Hill [1,2]

Marije Verheul [1,2]

Hannah Robinson[1,2]

Brian Angus [1,2]

Maheshi Ramasamy [1,2]

Myron M Levine[3]

Andrew J Pollard[1,2]

1- Oxford Vaccine Group, Department of Paediatrics, University of Oxford
2- NIHR Oxford Biomedical Research Centre, Oxford, UK
3- Department of Paediatrics, Center for Vaccine Development and Global Health, University of Maryland School of Medicine, Baltimore, MD, USA; Department of Medicine, Center for Vaccine Development and Global Health, University of Maryland School of Medicine, Baltimore, MD, USA

Corresponding author:

Dr Naina McCann

Naina.mccann@paediatrics.ox.ac.uk

07717890910

For peer review only

Present address: Oxford Vaccine Group, Centre for Clinical Vaccinology and Tropical Medicine, (CCVTM), Churchill Hospital, Old Road, Headington, Oxford, United Kingdom OX3 7LE

Keywords

Salmonella Paratyphi

Vaccine

Enteric fever

Challenge model

Word count: 4343

Abstract

Introduction

This is the first efficacy study of an oral live attenuated vaccine against *Salmonella* Paratyphi A using a human challenge model of paratyphoid infection. *Salmonella* Paratyphi A is responsible for 3.3 million cases of enteric fever every year, with over 19,000 deaths. Although improvements to sanitation and access to clean water are vital to reduce the burden of this condition, vaccination offers a cost-effective, medium-term solution. Efficacy trials of potential *S*. Paratyphi vaccine candidates in the field are unlikely to be feasible given the large number of participants required. Human challenge models therefore offer a unique, cost-effective solution to test efficacy of such vaccines.

Methods and analysis

This is an observer-blind, randomised, placebo-controlled trial Phase I/II of the oral live-attenuated vaccine against *S.* Paratyphi A, CVD 1902. Volunteers will be randomised 1:1 to receive two doses of CVD 1902 or placebo, 14 days apart. One month following second vaccination all volunteers will ingest *S.* Paratyphi A bacteria with a bicarbonate buffer solution. They will be reviewed daily in the following 14 days and diagnosed with paratyphoid infection if the pre-defined microbiological or clinical diagnostic criteria are met. All participants will be treated with antibiotics on diagnosis, or at day 14 post challenge if not diagnosed. The vaccine efficacy will be determined by comparing the relative attack rate, i.e. the proportion of those diagnosed with paratyphoid infection, in the vaccine and placebo groups.

Ethics and dissemination

Ethical approval for this study has been obtained from the Berkshire Medical Research Ethics committee (REC ref 21/SC/0330). The results will be disseminated via publication in a peer-reviewed journal and presentation at international conferences.

For peer review only - http://bmjopen.bmj.com/site/about/guidelines.xhtml

Trial registration number: ISRCTN15485902

Strengths and limitations of this study

- As a participant and observer blinded randomised control trial this study will provide high-quality evidence of the efficacy of an oral live attenuated *Salmonella* Paratyphi A vaccine candidate
- Immunology sampling throughout the trial will provide insight into the immune response following oral vaccination and infection with *S*. Paratyphi A which is so far poorly understood.
- One limitation of this study is that the trial population of healthy UK adults may not be representative of the target population for a paratyphoid vaccine, namely children in endemic areas.
- The model may under-estimate vaccine efficacy due to stringent diagnostic criteria

Introduction

Background

Enteric fever is a systemic febrile illness affecting 14.3 million individuals annually in 2017 (1). It is principally caused by *Salmonella enterica* serovars Typhi and Paratyphi A and is a common cause of morbidity, particularly in South and South East Asia. The proportion attributable to *Salmonella* Paratyphi A varies widely by geography but may be in the region of 25% (1), and is increasing in some areas (2–4). In the UK, S. Paratyphi represents 40-50% of travel-related enteric fever cases (5–7).

Control of enteric fever in endemic areas is challenged by a lack of sensitive diagnostic tests, rising antimicrobial resistance and limited access to safe drinking water. Vaccines are likely to be a cost-efficient way to reduce disease burden. The recent implementation of the typhoid conjugate vaccine in endemic countries is likely to have a significant impact on reducing *S*. Typhi incidence (8–10) and it is unclear what impact wider typhoid vaccination coverage may have on the rates of *S*. Paratyphi A infection (11). There are no current licensed vaccines against *S*. Paratyphi A.

Vaccine development for enteric fever has historically been hampered by the lack of an effective animal model to study the immunobiology of disease and the need for large sample sizes in efficacy trials. Controlled human infection models (CHIM) offer a unique alternative to study immunological responses to and the efficacy of such vaccines.

For peer review only - http://bmjopen.bmj.com/site/about/guidelines.xhtml

The Oxford Vaccine Group (OVG), has developed an enteric fever CHIM where over 400 participants have safely undergone challenge since 2010 (12–15). In recent years the OVG has developed the first *S.* Paratyphi A human challenge model, offering an opportunity to test the efficacy and immunological response to novel *S.* Paratyphi A vaccine candidates (15).

Several vaccines against *S*. Paratyphi A are in development, employing a range of approaches (16,17). Oral live-attenuated vaccines offer several immunological and practical advantages to parenteral vaccines, including ease of administration and induction of a broad immunological response. The University of Maryland has developed such a vaccine, CVD 1902, for which a one-dose regimen has been shown to be safe and immunogenic in humans, although efficacy remains unknown (5).

Study aims and objectives

This study aims to assess the efficacy of the orally administered live-attenuated vaccine CVD 1902 and study the immune response both to *S*. Paratyphi A infection and vaccination. This manuscript is written in accordance with protocol version 3.1, dated 11th April 2022. Primary, secondary and exploratory objectives and outcomes are outlined in Table 1.

For peer review only

For peer review only - http://bmjopen.bmj.com/site/about/guidelines.xhtml

Table 1. Primary, secondary, and exploratory objectives and outcomes of the VASP study

	**Objectives**	**Outcome Measures**
Primary	To determine the relative protective effect of two doses of CVD 1902 given 14 days apart compared with placebo (sodium bicarbonate) in a healthy adult paratyphoid challenge model	The proportion of participants developing clinical or microbiologically proven paratyphoid infection following oral challenge with *S*. Paratyphi A in the group who have received two doses of CVD 1902 compared with those who have received two doses of placebo
Secondary	a. To compare the clinical and laboratory features of the host responses following challenge with *Salmonella* Paratyphi A in participants vaccinated with CVD 1902 compared to placebo	Using clinical reporting, physical examination findings, microbiological and laboratory assays to compare the clinical course of paratyphoid infection after challenge between placebo and CVD 1902 groups
	b. To compare the host immune response following vaccination with CVD 1902, compared with placebo including innate, antibody and cell-mediated responses and persistence of immunity and to relate these responses to the protective effect of vaccination.	Immunological laboratory assays to assess innate, humoral, cell-mediated and mucosal responses to vaccination at baseline (Day -42) and post-vaccination time points
	c. To compare the host immune response following *S.* Paratyphi A challenge following vaccination with CVD 1902 or placebo.	Immunological laboratory assays to assess innate, humoral, cell-mediated and mucosal responses to challenge will be taken at various time points following challenge
	d. To assess the safety and tolerability of CVD 1902 including faecal shedding	Clinical observation and participant recording of symptoms, both solicited and unsolicited plus safety laboratory data and microbiological data from blood and stool cultures following vaccination
	e. To investigate immunological correlates of protection for *S*. Paratyphi A infection	Immunological response data post-vaccination will be combined with vaccine efficacy data following *S.* Paratyphi A challenge to investigate if particular immunological markers could be used to predict protection from paratyphoid infection

For peer review only

For peer review only - http://bmjopen.bmj.com/site/about/guidelines.xhtml

Methods

Study design and setting

This is an observer-blind, participant-blind, randomised, placebo-controlled trial of the oral live-attenuated vaccine CVD 1902 using a healthy adult participant controlled human infection model of paratyphoid.

In total 74-76 participants will be randomised in a 1:1 ratio to receive a dose of not less than 2 x 10^{10} CFU (Colony Forming Units) of CVD 1902 or placebo (sodium bicarbonate). As outlined in Figure 1, all participants will receive two doses of vaccine or placebo 14 days apart. Twenty-eight days after their second vaccine or placebo dose participants will be challenged with *S*. Paratyphi A (strain NVGH308) at a dose of 1-5 x 10^3 CFU, the dose previously established to give a desired clinical/laboratory 'attack' rate of approximately 60%(15).

Figure 1: Study design of the VASP trial investigating an oral vaccine against S. Paratyphi A

The study will be conducted at the Centre for Clinical Vaccinology and Tropical Medicine, Oxford, UK, which is a fully equipped vaccine research site with available clinical inpatient facilities and a Category III level laboratory on-site. The UK is non-endemic for enteric fever; cases are typically travel related (6).

Recruitment and eligibility

All potential participants may be contacted by methods including but not limited to email, telephone, posters, leaflets, websites, advertisements in newspaper, radio and on social media and/or mail using an approved invitation letter or other approved advertising material to invite them to participate in the study. Participants will be reimbursed for their time, travel and inconvenience.

Male or female participants between 18 and 55 years inclusive who are in good health (as determined by medical history, physical examination and clinical judgement of study team and their general practitioner) and willing and able to provide written informed consent will be eligible for inclusion. Multiple other strict inclusion and exclusion criteria will be applied during the screening process to minimise the risk to participants and to minimise the risk of onward transmission to others. A summarised list of inclusion and exclusion criteria is provided in Boxes 1 and 2 with a complete list provided in supplementary material 1.

For peer review only - http://bmjopen.bmj.com/site/about/guidelines.xhtml

Box 1. Summary of inclusion Criteria

Inclusion Criteria
Participants must satisfy all the following criteria to be considered eligible for the study:

- Willing and able to give informed consent for participation in the study.
- Aged between 18 and 55 years inclusive at time of vaccination.
- In good health as determined by medical history, physical examination and clinical judgment of the study team.
- Willing to be available in Oxford for all required appointments
- Agree to comply with all study requirements, including capacity to adhere to good personal hygiene and infection control precautions.
- Agree to allow study staff to contact his or her GP to access the participant's records and have their opinion solicited as to the participant's appropriateness for inclusion.
- Agree to allow study staff to access NHS health records as required for study purposes.
- Agree to allow his or her GP and UKHSA to be notified of participation in the study.
- Agree to give his or her close household contacts written information informing them of the participants' involvement in the study and offering them voluntary screening for *S.* Paratyphi A carriage.
- Agree to have 24-hour contact with study staff during the four weeks post challenge and are able to ensure that they are contactable by mobile phone for the duration of the vaccination and challenge period until antibiotic completion.
- Have internet access to allow completion of the e-diary and real-time safety monitoring.
- Agree to avoid antipyretic/anti-inflammatory treatment from challenge until advised by a study doctor or until 14 days after challenge.
- Agree to refrain from donating blood for the duration of the study.
- Agree to provide their National Insurance/Passport number for the purposes of TOPS registration and for payment
- Have received at least one dose of a SARS-CoV-2 vaccine $\geq$ four weeks prior to enrollment.
- Agree to not receive other vaccinations during the 7 days before and after study vaccination and during the 7 days before or 21 days post-challenge.

For peer review only

Box 2. Summary of Exclusion Criteria

Exclusion Criteria

The participant will not be enrolled if any of the following apply:

- History of significant organ/system disease that could interfere with trial conduct or completion
- Have any known or suspected impairment of immune function, alteration of immune function, or prior immune exposure that may alter immune function to paratyphoid
- HLA-B27 positive.
- Moderate or severe depression or anxiety
- Weight less than 50 kg.
- Presence of implants or prosthetic material.
- Anyone taking long-term medication that may affect symptom reporting or interpretation of the study results.
- Contraindication to fluoroquinolones, macrolide antibiotics, co-trimoxazole or ceftriaxone.
- Family history of aneurysmal disease
- Female participants who are pregnant, lactating or who are unwilling to ensure that they use effective contraception
- Full-time, part-time or voluntary occupations involving either direct contact with young children or highly susceptible patients or persons in whom typhoid infection would have particularly serious consequences, or commercial food handling
- Close household contact with young children or individuals who are immunocompromised (including pregnancy).
- Scheduled elective surgery or other procedures requiring general anaesthesia during the study period.
- Participants who have participated in another research study involving an investigational product that might affect risk of paratyphoid infection or compromise the integrity of the study within the 30 days prior to enrolment
- Detection of any abnormal results from screening investigations (at the clinical discretion of the study team).
- Inability to comply with any of the study requirements
- Any other social, psychological or health issues which, in the opinion of the study staff, may
    - put the participant or their contacts at risk because of participation in the study,
    - adversely affect the interpretation of the primary endpoint data,
    - Impair the participant's ability to participate in the study.
- Have any history of allergy to vaccine/placebo components
- Having been resident in an enteric fever endemic country for 6 months or more.
- Have previously been diagnosed with laboratory-confirmed typhoid or paratyphoid infection or been given a diagnosis compatible with enteric fever.
- Have participated in previous typhoid or paratyphoid challenge studies
- Have received any oral typhoid vaccination at any time.
- Have a prolonged corrected QT interval (>450 milliseconds) on ECG screening.
- Significant blood donation or planned blood donation prior to enrolment.

Participants will undergo a three-stage screening process to assess their suitability of the trial and to identify any of the inclusion and exclusion criteria. An online questionnaire will be followed by a telephone screening consultation and then a face-face consultation involving a physical examination, ECG, blood tests, urine dipstick, pregnancy test for females and gallbladder ultrasound examination. Temporary exclusion criteria will also be applied on the days of vaccination and challenge, as outlined in supplementary material 2.

For peer review only - http://bmjopen.bmj.com/site/about/guidelines.xhtml

Trial Procedures

Enrolment into the study will be day of first vaccination (D-42). Informed consent will be taken by an appropriately trained clinician (supplementary material 3). The detailed visit structure following enrolment can be found in the supplementary material 4.

Randomisation

Participants will be randomised 1:1 to receive CVD 1902 or placebo on Day -42. A statistician at OVG will generate a randomisation list using varying block sizes (2 and 4). The randomisation list aims to ensure equal target sample size is reached per group. The web-based randomisation system will ensure the allocation concealment is valid. Randomisation of participants will be carried out by unblinded study staff who are independent from the blinded team and do not perform any post vaccination procedures (such as ongoing eligibility, sample collection).

Blinding

This study will be conducted observer- and participant-blind from the time of randomisation until participant unblinding which will occur once the *last* participant has completed their Day 28 post-challenge visit. Observer and participant blinding is required to minimise the risk of bias on the reporting of adverse events following the administration of vaccine and challenge. Unblinding may occur at an earlier time point in the event of participant withdrawal, or the occurrence of any serious adverse events or reactions. This will be conducted under the guidance of the Data and Safety Monitoring Committee (DSMC).

Vaccination visits

Vaccine or placebo will be administered at the D-42 visit and the D-28 visit. Participants must have fasted for the 90 minutes prior to vaccination and pre-treatment bicarbonate solution will be administered in the minute preceding vaccination to neutralise stomach acid. Participants will be directly observed for a minimum of 30 minutes following vaccination and then asked to complete an e-diary of their symptoms daily for 7 days following each vaccination (supplementary material 5).

An in-person post-vaccination review will occur 7 days following each vaccine where participants will be reviewed for any possible adverse events and provide blood and stool samples.

Challenge visits

S. Paratyphi A challenge will be administered by the oral route following pre-treatment with sodium bicarbonate solution. Participants will be directly observed for 15 minutes following challenge and if they vomit within 60 minutes of challenge will be treated with antibiotics and withdrawn from the study.

For peer review only - http://bmjopen.bmj.com/site/about/guidelines.xhtml

Following challenge administration participants will be seen daily for 14 days and instructed to complete an online challenge e-diary to record oral temperatures twice- daily and to describe any symptoms or the use of any medications for 28 days (supplementary material 6).

Paratyphoid infection will be diagnosed based on certain pre-specified criteria as seen in Table 2.

Table 2. Criteria for diagnosis of paratyphoid following human challenge

Paratyphoid fever is diagnosed if ANY of the following apply
A positive blood culture for *S.* Paratyphi A from 72 hours post-challenge
A positive blood culture for *S.* Paratyphi A within 72 hours post-challenge, with one or more signs/symptoms of paratyphoid infection (such as recorded temperature $\geq 38.0^{o}$C)
Persistent positive blood cultures (two or more blood cultures taken at least 4 hours apart) for *S.* Paratyphi A within 72 hours post-challenge.
Oral temperature $\geq 38.0^{o}$C persisting for 12 hours in the 14 days following challenge

For peer review only

Once diagnosed, participants are treated with antibiotics and monitored for severity of infection. Participants will be provided with medications for symptomatic control if required. At any stage, if clinically indicated, an additional review will be arranged by the clinical study team or if severely unwell they will be directed to the local Accident and Emergency department (or appropriate other medical facility) and relevant medical personnel from the trial and secondary care will be made aware. Antibiotics may also be started if felt to be clinically necessary or participant has severe symptoms not meeting diagnostic criteria.

Follow-up visits

Participants will be followed up for 365 days following challenge. To detect chronic carriage of *S.* Paratyphi A and to confirm clearance, all participants are required to produce three stool samples obtained a minimum of 48 hours apart produced at least one week after completion of the antibiotic course. Participants with three successive negative stool samples will be considered to be fully treated for *S.* Paratyphi A infection and no longer an infection risk. The participants GP and local Health Protection Unit will be notified following challenge with *S*. Paratyphi A and then again once the participant has completed their clearance samples.

For peer review only - http://bmjopen.bmj.com/site/about/guidelines.xhtml

Laboratory testing

Samples will be taken throughout the trial for safety, microbiological and immunological assays which are summarised in supplementary material 7. Participants will be separately consented for their samples to be transferred to Oxford Vaccine Centre Biobank at the end of the study.

Data management

Data collection

Data will be collected on case report forms (CRFs) and all the study data will be recorded directly into REDCap or onto a paper source document for later entry if direct entry is not available.

Data confidentiality

The study will comply with the UK General Data Protection Regulation (GDPR) and Data Protection Act 2018. Participant data will be de-identified other than for uses about which the participants will be specifically consented for. Any electronic databases and documents with participant identifying details will be stored securely and will only be accessible by study staff and authorised personnel.

Monitoring & Audit

Data collection and storage will be inspected throughout the study by the quality assurance team at OVG and monitoring will be carried out by the study Sponsor, University of Oxford Research Governance, Ethics and Assurance (RGEA).

Trial interventions

Vaccine

CVD 1902 is a live attenuated strain of *Salmonella* Paratyphi A, an unlicensed, experimental oral vaccine for *Salmonella* Paratyphi A infection developed by the Center for Vaccine Development at the University of Maryland School of Medicine in Baltimore. It contains two independently attenuating mutations, one in the *guaBA* locus, and the other in *clpPX*. *GuaBA* encodes genes involved in guanine biosynthesis and *clpPX* encodes a protease which targets the master flagellar regulator. The product is manufactured, tested and labelled according to current EMA guidelines in keeping with Good Manufacturing Practice (GMP) by Bharat Biotech International Limited. The strain is fully sensitive to ciprofloxacin, trimethoprim/sulfamethoxazole and ampicillin (18). This vaccine has been tested in adults in a phase 1 trial at the University of Maryland where a total of 30 healthy young adults received a single oral dose of CVD 1902 in a single-site, randomised, double-blinded phase 1 study (18,19).

For peer review only - http://bmjopen.bmj.com/site/about/guidelines.xhtml

In this trial, vaccine recipients will receive 2 doses of CVD 1902 delivered in 30 mL carrier sodium bicarbonate solution, 14 days apart. Each dose will contain not less than 2 x 10^{10} CFU, with an upper limit of 1.7 x10^{11} CFU

The lower limit of the dose range for the vaccine in this trial corresponds to the highest dose administered in the previous Phase 1 trial which was shown to be safe and immunogenic (19). As higher doses were required in the Phase 1 study to elicit immune responses, a two-dose schedule has been chosen to optimise immunogenicity whilst also testing a schedule that ultimately may be feasible at a population level. The rationale for using two doses of CVD 1902 14 days apart is to try and ensure an optimum immune responses and protection as a previous study by Darton et al 2016 showed that a single-dose live attenuated oral vaccine for *Salmonella* Typhi M01ZH09 trialled within in a human challenge model did not provide protection, despite being immunogenic (20).

Placebo

The vaccine placebo is 30ml 1.3% wt/vol sodium bicarbonate solution made up using BP sodium bicarbonate powder with sterile water.

Challenge

The *S*. Paratyphi A challenge strain, NVGH308, was isolated from a participant in a clinical study performed by the Oxford University Clinical Research Unit at Patan Hospital, Kathmandu, Nepal. It has been manufactured into batches to GMP standard by GenIbet BioPharmaceuticals, Portugal, and is supplied to OVG by Novartis Vaccines for Global Health. The strain is fully sensitive to ciprofloxacin, azithromycin, ampicillin and trimethoprim/sulfamethoxazole.

Antibiotics

Antibiotics are used to treat diagnosed *Salmonella* Paratyphi A infection or at day 14 post challenge for those who have not been diagnosed.

The first line antibiotic will be oral ciprofloxacin 500mg twice daily for 7 days, in-line with guidance on enteric fever treatment (21). In fully susceptible *S*. Typhi isolates ciprofloxacin was shown to shorten time to resolution of symptoms, fever clearance time and bacteraemia when compared to azithromycin (22). Participants will be given verbal and written information regarding the possible side effects of ciprofloxacin use. For any participant in whom a contraindication to these first line antibiotics becomes apparent oral trimethoprim/sulfamethoxazole, oral azithromycin or oral amoxicillin will be used as second-line therapy.

Other trial interventions

Concomitant medication can be provided for symptomatic control of paratyphoid infection, before and after diagnosis. Concomitant medication after paratyphoid diagnosis includes antibiotics and antipyretics if required (supplementary material 8).

For peer review only

Sample size

Based on findings from the previous challenge studies performed at OVG, the assumption of attack rate of 58% was used in the control group (14,15). To demonstrate a protective effect of 70% (i.e. 30% relative risk in attacking rate), resulting in a reduction in attack rate from 58% in the control group to 17.4% in the vaccine group, 33 participants would be needed per group to achieve 90% power (1-β) at two-sided 5% significance level (α).

We observed less than a 10% of dropout rate from previous vaccine trial using typhoid challenge model (12). The sample size will be inflated to 37-38 participants per group to account for at least a 10%-dropout. We expect to randomise 74-76 participants in total.

Statistical analysis

The analysis of the primary end point will be a calculation of the proportion of participants diagnosed with paratyphoid fever in the CVD 1902 group relative to the placebo group. The proportion of participants diagnosed with paratyphoid fever (i.e., the attack rate) and the associated 95% confidence intervals will be presented by group at Day 28 after challenge. When calculating paratyphoid diagnosis proportions, the numerator will be the number of participants who meet the criteria for diagnosis and the denominator will be the per protocol population. The difference in proportions between the CVD 1902 and placebo groups will be analysed using Pearson's chi-squared test (or Fisher's Exact test if expected counts in any group are less than 5). To fulfil the primary objective, the protective effect of CVD 1902 over placebo will be calculated by:

$$PE = 100 \times (AR_{Placebo} – AR_{CVD\ 1902})/AR_{Placebo} = 100 \times (1 – AR_{CVD\ 1902}/ AR_{Placebo}),$$

Where PE is the protective effect and AR is the attack rate.

Time-to-event analyses of individual components of the primary outcome (positive blood culture and oral temperature ≥38.0°C) will be conducted using the Kaplan-Meier method and will include all participants. Participants not meeting the criteria for an individual component of the primary endpoint will be censored in the analysis at the final monitoring for those undiagnosed. Participants who withdrew or had potential interference with vaccine effect or infection challenge, (e.g. treated prior to Day 14 with no diagnosis of paratyphoid) will be censored in the analysis at the time of withdrawal or interference.

Immunogenicity data are expected to be highly skewed and will be log-transformed prior to analysis. Results will be presented as geometric means with 95% confidence intervals. Values below the limit of detection (LLOD) will be imputed with half the value of the LLOD.

For peer review only

Ethics and dissemination

Ethical approval, regulation, and governance

Ethical approval for this study has been obtained from the Berkshire Research Ethics Committee and Health Research Authority (REC reference: 21/SC/0330) and the study will run in accordance with the ethical principles of the Declaration of Helsinki.

The use of an unlicensed vaccine in this study, CVD1902, is regulated by the Medicines for Human Use (Clinical Trials) Regulations 2004 (MHRA). As CVD 1902 is a genetically modified organism, (GMO), relevant approvals have been obtained for its use in this study from the Department of Environment, Food and Rural Affairs (DEFRA), Oxford University Hospitals NHS Foundation Trust (OUH) and the Centre for Clinical Vaccinology and Tropical Medicine (CCTVM). The challenge agent falls outside the remit of these regulators and therefore is judged according to common law and best practice (23). Regional ethics committees therefore provide support in governing challenge studies.

An independent DSMC consisting of an experienced group of infectious disease clinicians, scientists and a statistician will be appointed to provide real-time oversight of safety and trial conduct. The DSMC will review safety data collated from the eCRFs and e-diaries including solicited and unsolicited symptoms, laboratory results and vital signs. Following prime vaccination of a sentinel group of six participants relevant safety data will be reviewed by the DSMC. If there are no safety concerns these participants can proceed to their second vaccination and further participants can begin vaccination. Further DSMC reviews will occur regularly throughout the trial.

A development safety update report (DSUR) for the IMP will be prepared annually, on the anniversary of the MHRA approval for the trial.

Risks

The general risks to participants in this study are associated with potential side effects from the vaccine or placebo, symptomatic infection following challenge and the small risk of subsequent complications from paratyphoid infection. Additionally, there is a small risk of secondary transmission of *S*. Paratyphi A to close contacts of participants. The University has a specialist insurance policy in place, which would operate in the event of any participant suffering harm as a result of their involvement in the research.

Vaccine

In the Phase 1 trial "CVD 1902 was well tolerated without clinically significant adverse reactions attributed to the vaccine" (18). Hypersensitivity to the vaccine is extremely unlikely given the constituents present in the vaccine preparation but there may be unforeseeable side effects. A study

doctor will check participants post-vaccination symptoms daily in relation to the individual and trial stopping rules (supplementary material 9).

Challenge

Some study participants will develop symptomatic paratyphoid infection following challenge, for which risks include development of severe disease or complications. These risks will be greatly minimised by daily reviews by a clinical study team member, daily review of e-diary entries, regular safety bloods and early treatment with an effective antimicrobial. A previous challenge study using the same strain of *S.* Paratyphi A at OVG has demonstrated a good safety profile (15).

Carriage

The likelihood of developing chronic carriage is extremely low in the challenge setting. Participants are treated with ciprofloxacin, a fluoroquinolone antibiotic which is the preferred class of antibiotics for prevention of chronic carriage (24). To ensure clearance of infection and to exclude chronic carriage, stool samples for culture will be obtained upon completion of the initial antibiotic course. If participants remain positive after a second course of antibiotic treatment then participants will be referred to an Infectious Diseases Consultant at the Oxford University Hospitals NHS Foundation Trust for further management.

Close contacts

In view of the low infectivity of *S.* Paratyphi A without bicarbonate buffer and the high standard of hygiene and sanitation in the UK, secondary transmission of the challenge strain to household or other close contacts after discharge is highly unlikely. It is thought that typhoidal Salmonellas, unlike *Shigella sp.*, enterohaemorrhagic *Escherichia coli,* or hepatitis A virus, are virtually never transmitted by direct faecal-oral contact. This is in part due to the higher oral inoculum of these bacteria required to cause clinical disease.

It is acknowledged, however, that transmission within households can occur if the individual excreting *S.* Paratyphi A fails to practice effective hand washing after defecation and is subsequently involved in uncooked food preparation. Therefore, throughout the period of possible excretion of the challenge strain, participants must practice stringent hand washing techniques after defecation. Participants will be given soap and paper towels for use at home and detailed advice on how to prevent transmission of *S.* Paratyphi A. Participants will be taught and observed practising good hygiene technique at their initial challenge visit.

Adverse event reporting

All adverse events occurring from enrolment to day 28 post-challenge will be recorded and graded (supplementary material 10). Solicited adverse events (AEs) will be recorded and graded (0-4) by the

For peer review only

participant in a vaccine e-diary for 7 days post-vaccination and a challenge e-diary 21 days post-challenge. Solicited AEs will be reviewed daily during the periods of recording by a study clinician and if they have concerns about the severity or frequency of an event this will be followed up with the participant. Unsolicited AEs will be recorded by the participant in the e-diaries any time from first vaccine administration until day 28 post-challenge.

All solicited AEs recorded in the e-diary will be automatically assumed to be related to the vaccine or challenge (depending on whether recorded in vaccine diary or challenge diary respectively) whereas all unsolicited AEs will undergo causality assessment by a clinician as to whether related to the vaccine, challenge agent or other medications.

Laboratory results and vital signs will be entered directly into an eCRF and automatically severity graded. Any clinically significant results will be reported as adverse events and causality assessed.

Medically attended non-serious AEs occurring between day 28 and day 90 elicited at the day 90 visit will not receive a causality assessment. All serious adverse events (SAEs) will be recorded from time of consent and reported to the DSMC within 24 hours of notification. SAEs will receive a causality assessment throughout the study, at the time of reporting. All SAEs at least possibly related to CVD 1902 will be considered unexpected and be reported to the MHRA and REC as serious unexpected serious adverse reactions within the regulatory timelines. Adverse events of special interest (AESIs) are listed in table 3. These will be reported to the DSMC as soon as possible but within 7 days of discovery.

Table 3: Adverse events of special interest in the VASP trial

Adverse events of special interest
Severe paratyphoid infection*
Failure to clinically or bacteriologically cure a participant of paratyphoid infection
Progression to chronic carrier state
Relapse of paratyphoid infection
Transmission of S. Paratyphi A to a contact of a participant
AEs requiring a physician visit or Emergency Department visit which, in opinion of study staff, are related to challenge with S. Paratyphi A
Pregnancy

*Severe paratyphoid infection defined as illness that includes any of the following criteria: oral temperature > 40 degrees celcius, systolic blood pressure <u><85</u> mmHg, significant lethargy or confusion, gastrointestinal bleeding, gastrointestinal perforation or any grade 4 or above laboratory abnormality.

For peer review only - http://bmjopen.bmj.com/site/about/guidelines.xhtml

Dissemination

The trial protocol is registered on the ISRCTN registry (ISRCTN15485902). Dissemination of the findings is planned for publication in peer-reviewed journals and at scientific international conferences.

Patient and public involvement

The protocol, study information booklet and recruitment materials were reviewed by a local patient consultation group who provided feedback and comments on the initial documents. Their comments led to changes in the participant-facing documents, ensuring they are easy and clear for participants to understand.

Discussion

This study will be the first efficacy analysis of a live-attenuated oral vaccine against *S.* Paratyphi A using a human challenge model. Resulting data has the potential to be a critical step in the future licensure of the first vaccine against *S.* Paratyphi A. The role of a *S.* Paratyphi A vaccine is most likely to be part of a bi- or even multi-valent vaccine against both typhoidal and potentially non-typhoidal serovars. Deployment of a bivalent vaccine against S. Typhi and S. Paratyphi A would address the burden of enteric fever within an Asian context where both pathogens are endemic. Other areas, such as sub-Saharan Africa non-typhoidal serovars cause a significant burden of disease hence a multi-valent vaccine incorporating typhoidal and non-typhoidal serovars may prove more cost-effective as a single approach regardless of geographical setting. However, in order to contribute to this vaccine development, an efficacious S. Paratyphi A vaccine is required. This trial will act as a proof of concept efficacy trial for such a vaccine, CVD 1902.

Reliable immunologic correlates of protection for *S*. Typhi and *S*. Paratyphi A have not yet been identified. Progress has been made in *S*. Typhi with data from prior *S*. Typhi challenge trials identifying anti-Vi IgA and IgG1 antibodies correlated with protection following Vi-TT and Vi-PS vaccination (25,26). However, *S*. Paratyphi A lacks the Vi antigen and it's immunobiology and pathogenesis is less well understood than it is for *S*. Typhi. The lipopolysaccharide O:2 has been identified as an important virulence factor for *S*. Paratyphi and the flagellar (H) antigens may also play a role in protection. During this trial we will evaluate both the humoral and cellular response to both CVD 1902 and *S*. Paratyphi challenge and in doing so provide invaluable immunological data and further our understanding of immunity to *S*. Paratyphi infection and correlates of protection.

As a controlled human infection model the conditions during this trial will differ from endemic areas. The population in this trial is healthy UK adults whereas the majority of paratyphoid infections in endemic areas occur in young children. Although different physiologically, these groups may be similar from an immunological perspective as they are both paratyphoid naïve. The stringent

For peer review only - http://bmjopen.bmj.com/site/about/guidelines.xhtml

diagnostic criteria in this trial may also underestimate vaccine efficacy by picking up sub-clinical bacteraemia, whilst patients in endemic areas are more likely to be diagnosed on symptoms alone. Nevertheless, the results of the typhoid conjugate vaccine human challenge study showed that this model can be used to provide early, representative data on vaccine efficacy that does translate to efficacy in field studies (8,12).

Although no licensed vaccines against *S.* Paratyphi A exist there are several other mono and bivalent vaccines in development. Conjugate vaccines use the lipopolysaccharide O-antigen (O:2) of paratyphoid conjugated to a protein carrier, either to tetanus-toxoid or CRM_{197} a nontoxic mutant of diptheria toxin. Phase I/II studies of O:2-TT showed this vaccine was safe and immunogenic and further phase II trials are currently underway(27) with a bivalent formation combined with Vi-TT also in development. Pre-clinical studies demonstrated O:2-CRM_{197} was immunogenic when used alone or with Vi-CRM_{197} Novel vaccines, such as nanoparticle-based vaccines and MAPS-based vaccines are also in development (28).

As the first trial using a human challenge model to test efficacy of a S. Paratyphi vaccine, this trial paves the way for potential testing of other *S*. Paratyphi A vaccine candidates. Alongside improvements in sanitation and access to safe water supply, the development of an effective vaccine against *S*. Paratyphi A, has the potential to significantly impact on the burden of enteric fever in endemic countries.

Contributors

AJP, BA and MNR are the chief and principal investigators, who alongside MML, conceived and developed the study. NM and KE are research fellows who drafted the manuscript and protocol. XL, MG and KC are the study statisticians. FM, SC, EJ, YCK, JH, MV contributed to the laboratory aspects of the study design. NS and HR helped in design of the final study protocol and co-ordinated regulatory approvals. All authors read and approved the final manuscript.

Funding

This work was supported by Medical Research Council, grant number MR/RO25347/1.

Competing interests' statement

AJP is chair of the UK Department of Health and Social Care's (DHSC) Joint Committee on Vaccination and Immunisation (JCVI), was a member of WHOs Strategic Advisory Group of Experts until 2022, receives consulting fees from Shionogi and is in receipt of grants from MRC, AstraZeneca, Gates Foundation, Wellcome Trust, NIHR and European Commission through the University of

For peer review only

For peer review only - http://bmjopen.bmj.com/site/about/guidelines.xhtml

Oxford. MML is a co-investigator on the MRC grant for this study, holds patents for Attenuated Salmonella Enterica Serovar Paratyphi A and Uses Thereof in the US and UK, Germany, France, Italy and Spain, is a member of the FDA Vaccines and Related Biological Products Advisory Committee (VRBPAC) and a member of the NIH Data and Safety Monitoring Board (DSMB) to review and monitor US-government-supported clinical trials of candidate COVID-19 vaccines (2020 to present). MNR works as a principal investigator on clinical vaccine trials funded by AstraZeneca and Moderna but receives no personal financial payment for this work. All other authors declare no competing interests.

References

1. Stanaway JD, Reiner RC, Blacker BF, Goldberg EM, Khalil IA, Troeger CE, et al. The global burden of typhoid and paratyphoid fevers: a systematic analysis for the Global Burden of Disease Study 2017. Lancet Infect Dis [Internet]. 2019 Apr 1 [cited 2021 Mar 17];19(4):369–81. Available from: http://dx.doi.org/10.1016/
2. Sood S, Kapil A, Dash N, Das BK, Goel V, Seth P. Paratyphoid fever in India: An emerging problem [3]. Emerging Infectious Diseases. 1999.
3. Kuijpers LMF, Veng CH, Sar D, Chung P, Phe T, Kham C, et al. Ongoing outbreak of Salmonella enterica serovar Paratyphi A infections, Phnom Penh, Cambodia. Journal of Infection in Developing Countries. 2015.
4. Tankhiwale SS, Agrawal G, Jalgaonkar S V. An unusually high occurrence of Salmonella enterica serotype Paratyphi A in patients with enteric fever. Indian J Med Res. 2003;
5. Herdman MT, Karo B, Dave J, Katwa P, Freedman J, Nascimento V Do, et al. Increasingly limited options for the treatment of enteric fever in travellers returning to England, 2014-2019: A cross-sectional analytical study. J Med Microbiol. 2021;
6. Typhoid and paratyphoid: laboratory confirmed cases in England, Wales and Northern Ireland - GOV.UK [Internet]. [cited 2022 May 4]. Available from: https://www.gov.uk/government/publications/typhoid-and-paratyphoid-laboratory-confirmed-cases-in-england-wales-and-northern-ireland
7. McCann N, Nabarro L, Morris-Jones S, Patel T, Godbole G, Heyderman R, et al. Outpatient management of uncomplicated enteric fever: A case series of 93 patients from the Hospital of Tropical Diseases, London. J Infect [Internet]. 2022 Jun [cited 2022 Aug 13];0(0). Available from: http://www.journalofinfection.com/article/S0163445322003814/fulltext
8. Shakya M, Voysey M, Theiss-Nyland K, Colin-Jones R, Pant D, Adhikari A, et al. Efficacy of typhoid conjugate vaccine in Nepal: final results of a phase 3, randomised, controlled trial. Lancet Glob Heal. 2021;
9. Patel PD, Patel P, Liang Y, Meiring JE, Misiri T, Mwakiseghile F, et al. Safety and Efficacy of a Typhoid Conjugate Vaccine in Malawian Children. N Engl J Med. 2021;
10. Qadri F, Khanam F, Liu X, Theiss-Nyland K, Biswas PK, Bhuiyan AI, et al. Protection by

For peer review only

vaccination of children against typhoid fever with a Vi-tetanus toxoid conjugate vaccine in urban Bangladesh: a cluster-randomised trial. Lancet. 2021;

11. Dong B-Q, Yang J, Wang X-Y, Gong J, von Seidlein L, Wang M-L, et al. Trends and disease burden of enteric fever in Guangxi province, China, 1994–2004. Bull World Health Organ. 2010;
12. Jin C, Gibani MM, Moore M, Juel HB, Jones E, Meiring J, et al. Efficacy and immunogenicity of a Vi-tetanus toxoid conjugate vaccine in the prevention of typhoid fever using a controlled human infection model of Salmonella Typhi: a randomised controlled, phase 2b trial. Lancet. 2017;
13. Gibani MM, Jones E, Barton A, Jin C, Meek J, Camara S, et al. Investigation of the role of typhoid toxin in acute typhoid fever in a human challenge model. Nat Med. 2019;
14. Gibani MM, Jin C, Shrestha S, Moore M, Norman L, Voysey M, et al. Homologous and heterologous re-challenge with salmonella typhi and salmonella paratyphi a in a randomised controlled human infection model. PLoS Negl Trop Dis. 2020;
15. Dobinson HC, Gibani MM, Jones C, Thomaides-Brears HB, Voysey M, Darton TC, et al. Evaluation of the clinical and microbiological response to salmonella paratyphi a infection in the first paratyphoid human challenge model. Clin Infect Dis. 2017;
16. Martin LB, Simon R, MacLennan CA, Tennant SM, Sahastrabuddhe S, Khan MI. Status of paratyphoid fever vaccine research and development. Vaccine. 2016;
17. Shakya M, Neuzil KM, Pollard AJ. Prospects of Future Typhoid and Paratyphoid Vaccines in Endemic Countries. J Infect Dis [Internet]. 2021 Dec 20 [cited 2022 Mar 3];224(Supplement_7):S770–4. Available from: https://academic.oup.com/jid/article/224/Supplement_7/S770/6347515
18. Center for Vaccine Development. Investigator's Brochure: Strain CVD 1902. Univ Maryl Sch Med. 2018;
19. Wahid R, Kotloff KL, Levine MM, Sztein MB. Cell mediated immune responses elicited in volunteers following immunization with candidate live oral Salmonella enterica serovar Paratyphi A attenuated vaccine strain CVD 1902. Clin Immunol. 2019;
20. Darton TC, Jones C, Blohmke CJ, Waddington CS, Zhou L, Peters A, et al. Using a Human Challenge Model of Infection to Measure Vaccine Efficacy: A Randomised, Controlled Trial Comparing the Typhoid Vaccines M01ZH09 with Placebo and Ty21a. PLoS Negl Trop Dis [Internet]. 2016 Aug 17 [cited 2022 Mar 3];10(8):e0004926. Available from: https://journals.plos.org/plosntds/article?id=10.1371/journal.pntd.0004926
21. Nabarro LE, McCann N, Herdman MT, Dugan C, Ladhani S, Patel D, et al. British infection association guidelines for the diagnosis and management of enteric fever in England. J Infect [Internet]. 2022 Jan [cited 2022 Feb 2];0(0). Available from: http://www.journalofinfection.com/article/S0163445322000135/fulltext
22. Jin C, Gibani MM, Pennington SH, Liu X, Ardrey A, Aljayyoussi G, et al. Treatment responses to azithromycin and ciprofloxacin in uncomplicated Salmonella Typhi infection: A comparison of clinical and microbiological data from a controlled human infection model. PLoS Negl Trop

For peer review only

For peer review only - http://bmjopen.bmj.com/site/about/guidelines.xhtml

Dis. 2019;

23. Academy of Medical Sciences. Microbial challenge studies of human volunteers. Acad Med Sci London. 2005;

24. Ferreccio C, Morris JG, Valdivieso C, Prenzel I, Sotomayor V, Drusano GL, et al. Efficacy of ciprofloxacin in the treatment of chronic typhoid carriers. J Infect Dis. 1988;

25. Dahora LC, Jin C, Spreng RL, Feely F, Mathura R, Seaton KE, et al. IgA and IgG1 Specific to Vi Polysaccharide of Salmonella Typhi Correlate With Protection Status in a Typhoid Fever Controlled Human Infection Model. Front Immunol. 2019;

26. Jin C, Hill J, Gunn BM, Yu WH, Dahora LC, Jones E, et al. Vi-specific serological correlates of protection for typhoid fever. J Exp Med. 2020;

27. Konadu E, Shiloach J, Bryla DA, Robbins JB, Szu SC. Synthesis, characterization, and immunological properties in mice of conjugates composed of detoxified lipopolysaccharide of Salmonella paratyphi A bound to tetanus toxoid, with emphasis on the role of O acetyls. Infect Immun. 1996;

28. Immunization, Vaccines and Biologicals [Internet]. [cited 2022 Mar 11]. Available from: https://www.who.int/teams/immunization-vaccines-and-biologicals/diseases/paratyphoid-fever

For peer review only

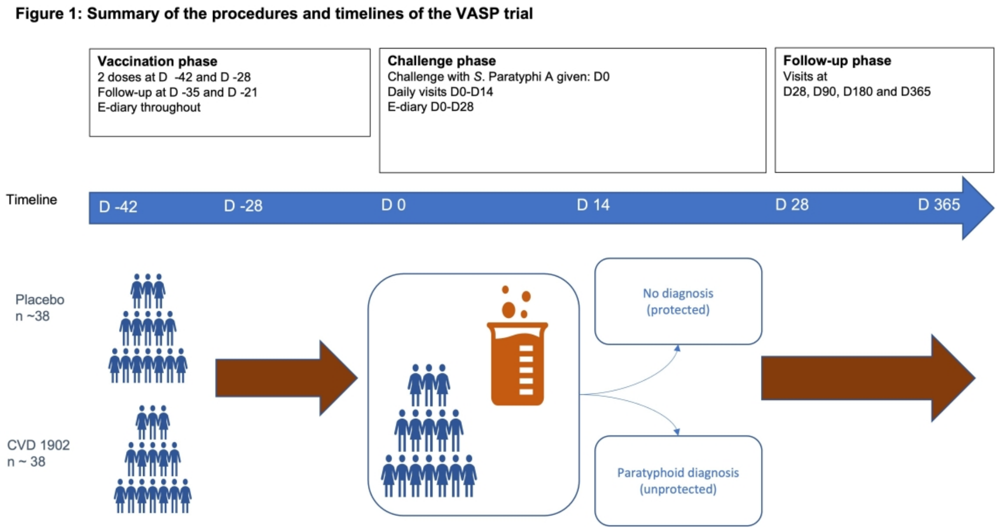

Figure 1: Summary of the procedures and timelines of the VASP Trial

309x163mm (330 x 330 DPI)

Supplementary Material 1: Inclusion and Exclusion Criteria

Inclusion Criteria

Participants must satisfy all the following criteria to be considered eligible for the study:

- Willing and able to give informed consent for participation in the study.
- Aged between 18 and 55 years inclusive at time of vaccination.
- In good health as determined by medical history, physical examination and clinical judgment of the study team.
- Willing to be available in Oxford for all required appointments
- Agree (in the study team's opinion) to comply with all study requirements, including capacity to adhere to good personal hygiene and infection control precautions.
- Agree to allow study staff to contact his or her GP to access the participant's vaccination records, medical history and have their opinion solicited as to the participant's appropriateness for inclusion.
- Agree to allow study staff to access NHS health records as required for study purposes.
- Agree to allow his or her GP (and/or Consultant if appropriate), to be notified of participation in the study.
- Agree to allow UKHSA to be informed of their participation in the study.
- Agree to give his or her close household contacts written information informing them of the participants' involvement in the study and offering them voluntary screening for *S.* Paratyphi A carriage.
- Agree to have 24-hour contact with study staff during the four weeks post challenge and are able to ensure that they are contactable by mobile phone for the duration of the vaccination and challenge period until antibiotic completion.
- Have internet access to allow completion of the e-diary and real-time safety monitoring.
- Agree to avoid antipyretic/anti-inflammatory treatment from challenge until advised by a study doctor or until 14 days after challenge.
- Agree to refrain from donating blood for the duration of the study.
- Agree to provide their National Insurance/Passport number for the purposes of TOPS registration and for payment of reimbursement expenses.
- Participants must have received at least one dose of a SARS-CoV-2 vaccine that has been approved for use by the MHRA (or other national regulatory authority) $\geq$ four weeks prior to enrollment.

For peer review only

- Agree to not receive other vaccinations (eg Covid-19 vaccines) during the 7 days before and after study vaccination and during the 7 days before or 21 days post-challenge.

1.1. Exclusion Criteria

The participant will not be enrolled if any of the following apply:

- History of significant organ/system disease that could interfere with trial conduct or completion. Including, for example, but not restricted to:
  - Cardiovascular disease including a diagnosis of hypertension
  - Respiratory disease
  - Haematological disease[1]
  - Endocrine disorders
  - Renal or bladder disease, including history of renal calculi
  - Biliary tract disease, including biliary colic, asymptomatic gallstones or previous cholecystectomy
  - Gastro-intestinal disease including requirement for antacids, H_2-receptor antagonists, proton pump inhibitors or laxatives
  - Neurological disease
  - Metabolic disease
  - Autoimmune disease
  - Psychiatric illness requiring hospitalisation
  - Known or suspected drug misuse
  - Known or suspected alcohol misuse (alcohol misuse defined as an intake exceeding 42 units per week)
  - Infectious disease
- Have any known or suspected impairment of immune function, alteration of immune function, or prior immune exposure that may alter immune function to paratyphoid resulting from, for example:
  - Congenital or acquired immunodeficiency, including IgA deficiency
  - Human Immunodeficiency Virus infection or symptoms/signs suggestive of an HIV-associated condition

---

[1] This includes anaemia. The acceptable lower limits for [haemoglobin] are 125 g/L for female participants and 135 g/L for male participants (Guidelines for the Blood Transfusion Services in the UK, 8th edition, 2018 <https://www.transfusionguidelines.org/red-book> Accessed 5th December 2018).

For peer review only

- Receipt of immunosuppressive therapy such as anti-cancer chemotherapy or radiation therapy within the preceding 12 months or long-term systemic corticosteroid therapy.
- Receipt of immunoglobulin or any blood product transfusion within 3 months of study start.
- History of cancer (except squamous cell or basal cell carcinoma of the skin and cervical carcinoma in situ).

- HLA-B27 positive.
- Moderate or severe depression or anxiety as classified by the Hospital Anxiety and Depression Score at screening or challenge that is deemed clinically significant by the study doctors[2].
- Weight less than 50 kg.
- Presence of implants or prosthetic material.
- Anyone taking long-term medication (e.g. analgesia, anti-inflammatories or antibiotics) that may affect symptom reporting or interpretation of the study results.
- Contraindication to fluoroquinolones, macrolide antibiotics, co-trimoxazole or ceftriaxone.
- Family history of aneurysmal disease
- Female participants who are pregnant, lactating or who are unwilling to ensure that they or their partner use effective contraception[3] 30 days prior to vaccination and continue to do so until three negative stool samples have been obtained after completion of antibiotic treatment.
- Full-time, part-time or voluntary occupations involving:
  - Clinical or social work with direct contact with young children (defined as those attending pre-school groups or nursery or aged under 2 years), or
  - Clinical or social work with direct contact with highly susceptible patients or persons in whom typhoid infection would have particularly serious consequences
  - Commercial food handling (involving preparing or serving unwrapped foods not subjected to further heating)

(unless willing to avoid work from vaccination until demonstrated not to be infected with *S.* Paratyphi A after challenge by clearance samples in accordance with guidance from UKHSA and willing to allow study staff to inform their employer).

---

[2] If elevated scores are due to temporary significant life events, the questionnaire may be repeated after resolution of the event with a view to inclusion if normal.

[3] As defined by CTFG Recommendations related to contraception and pregnancy testing in clinical trials, current document: https://www.hma.eu/fileadmin/dateien/Human_Medicines/01-About_HMA/Working_Groups/CTFG/2014_09_HMA_CTFG_Contraception.pdf [accessed 23rd July 2019]

- Close household contact with:
    - Young children (defined as those attending pre-school groups, nursery or those aged less than 2 years)
    - Individuals who are immunocompromised (including pregnancy).
- Scheduled elective surgery or other procedures requiring general anaesthesia during the study period.
- Participants who have participated in another research study involving an investigational product that might affect risk of paratyphoid infection or compromise the integrity of the study within the 30 days prior to enrolment (e.g. significant volumes of blood already taken in previous study)[4].
- Detection of any abnormal results from screening investigations (at the clinical discretion of the study team).
- Inability to comply with any of the study requirements (at the discretion of the study staff and the participant's General Practitioner).
- Any other social, psychological or health issues which, in the opinion of the study staff, may
    - put the participant or their contacts at risk because of participation in the study,
    - adversely affect the interpretation of the primary endpoint data,
    - Impair the participant's ability to participate in the study.
- Have any history of allergy to vaccine/placebo components
- Having been resident in an enteric fever endemic country for 6 months or more.
- Have previously been diagnosed with laboratory-confirmed typhoid or paratyphoid infection or been given a diagnosis compatible with enteric fever.
- Have participated in previous typhoid or paratyphoid challenge studies (with ingestion of challenge agent).
- Have received any oral typhoid vaccination (e.g. Ty21a or M01ZH09) at any time.
- Have a prolonged corrected QT interval (>450 milliseconds) on ECG screening.
- Significant blood donation or planned blood donation prior to enrolment.

[4] As assessed by both participant questioning and registration with The Over Volunteering Prevention System (TOPS) database.

For peer review only

Supplementary Material 2: Temporary Exclusion Criteria

Temporary Exclusion at Vaccination

Participants will be temporarily excluded from receiving vaccination if presenting at a vaccination visit with the following:

- Acute gastrointestinal illness within 24-hours prior to vaccination.
- Significant infection within the previous 7 days.
- Participant has experienced fever (>37.5°C) or subjective febrile symptoms within the previous 3 days (even with a negative COVID-19 test).
- Symptoms of COVID-19, or confirmed infection (according to government guidelines) within 14 days prior to vaccination visit; as per guidance in Section 9.11
- Validated positive SARS-CoV-2 test (NAAT or antigen) within 2 weeks prior to vaccination visit
- History of any antibiotic therapy during the previous 5 days.
- Any systemic corticosteroid (or equivalent) treatment in the previous 14 days, or for more than seven consecutive days within the past 3 months).
- Receipt of another enteric live vaccine within 4 weeks prior to vaccination or an injected live or killed vaccine within 7 days prior to vaccination.
- Plan to receive any vaccine other than the study vaccine within 7 days following vaccination.
- Therapy with antacids, proton pump inhibitors or H_2-receptor antagonists within 24 hours prior to vaccination.
- Unavailable for post-vaccination visits, second vaccination visit, and challenge visit as outlined in study procedures table (see section 7.4 table 1b).

If this is the first vaccine to be received and the temporary exclusion does not result in the participant becoming ineligible, then this vaccine visit can be rescheduled.

If this is the second vaccine to be received and the temporary exclusion does not result in the participant becoming ineligible, then this should be discussed with a study doctor as to whether they can be rescheduled for their second vaccination (see table 2, section 7.5 for visit windows) or withdrawn.

Temporary Exclusion at Challenge

For peer review only

Participants will be temporarily excluded from challenge if presenting at the challenge visit with the following. Participants can be challenged up to 28 days after their original challenge date (see table 2, section 7.5)

- Significant acute or acute-on-chronic infection within the previous 7 days or have experienced fever (>37.5□C) or subjective febrile symptoms within the previous 3 days (even with a negative COVID-19 test).
- Symptoms of COVID-19, or confirmed infection (according to government guidelines) within 14 days prior to challenge visit; as per guidance in Section 9.11
- Validated positive SARS-CoV-2 test (NAAT or antigen) within 2 weeks prior to challenge visit
- History of any antibiotic therapy during the previous 5 days.
- Any systemic corticosteroid (or equivalent) treatment in the previous 14 days, or for more than seven consecutive days within the past 3 months.
- Therapy with antacids, proton pump inhibitors or H_2-receptor antagonists within 24 hours prior to challenge.
- Participant has not received two doses of the study vaccine/placebo.
- Anaemia felt to be clinically significant by the study team.
- Receipt of a COVID-19 vaccine in preceding 7 days
- Plan to receive any vaccine within 21 days following challenge.

For peer review only

Oxford Vaccine Group
University of Oxford
Centre for Clinical Vaccinology and Tropical Medicine,
Churchill Hospital, Headington, Oxford OX3 7LE
Tel: 01865 611400 info@ovg.ox.ac.uk www.ovg.ox.ac.uk

Development of a vaccine against *Salmonella* Paratyphi A (VASP)

Informed Consent Form

Participant's Name: ____

Participant Initials: __________

Participant screening number: | S | P | T | — | 0 | 6 | — | | | | |

Name of Researcher: ***If you agree, please initial box:***

**Section 1: Study Procedures.**	
1. I confirm that I have read the information sheet dated................... (version............) for this study. I have had the opportunity to consider the information, ask questions and have had these answered satisfactorily	
2. I have spoken with Dr/Nurse_______________________________________	
3. I understand that my participation is voluntary and that I am free to withdraw at any time without giving any reason, without my medical care or legal rights being affected.	
4. I have received detailed information about the treatment schedule and potential side effects.	
5. I agree to be randomised to receive either the experimental vaccine CVD 1902 or the placebo and I am aware of the risks and side effects associated with vaccination	
6. Should I wish to withdraw after I have been challenged with *Salmonella* Paratyphi A, I understand that I must take a course of antibiotics and will be asked to attend for further visits for safety reasons and that if I fail to do so United Kingdom Health Security Agency (UKHSA) will be informed.	
7. I understand that should I fail to return for review or to take the full course of antibiotics I may become seriously ill and could even die.	
8. I will bring the 24-hour contact reply slip to the first study visit, signed by my 24-hour contact. I agree that the study team may contact this person if I cannot be contacted during the study	

For peer review only
For peer review only - http://bmjopen.bmj.com/site/about/guidelines.xhtml

9. I agree to refrain from donating blood for the duration of the study.	
**Section 2: Personal Information:**	
10. I agree to my GP being informed of my participation in this study including information about diagnosis, treatment and clearance samples.	
11. I agree to my GP and/or other treating doctors being approached for additional information regarding my medical and vaccination history and study staff to access my NHS medical records.	
12. I understand that United Kingdom Health Security Agency (UKHSA) will be informed of my participation in this study including information about diagnosis, treatment and clearance samples.	
13. I understand that relevant sections of my medical notes and data collected during the study may be looked at by individuals from University of Oxford, from regulatory authorities [and from the NHS Trust(s)], where it is relevant to my taking part in this research. I give permission for these individuals to have access to my records.	
14. I understand TOPS is the Health Research Authority database that aims to prevent healthy volunteers from taking part in too many studies. I understand that only staff at OVG and other research units can use the database and OVG may call other units, or OVG may be called, to check volunteer details. I agree to my National Insurance (if UK citizen) or Passport number being used to register me on TOPS. I understand that it will be stored electronically for the duration of the study.	
**Section 3: Research Samples**	
15. I agree to donate saliva, blood and stool samples. I consider these samples a gift to the University of Oxford and I understand I will not gain any direct personal or financial benefit from them.	
16. I understand that my anonymised data and biological samples may be sent and stored within and outside of the European Union for analysis by collaborating research groups as described in the information booklet	
17. I understand and agree that my samples will be used in research aimed at understanding the genetic influences on *Salmonella* Paratyphi A and that the results of these investigations are unlikely to have any implications for me personally.	
**Section 4: Occupational**	
18. I understand that I should not be involved in commercial food handling until I am shown not to be infected with *Salmonella* Paratyphi A	

For peer review only - http://bmjopen.bmj.com/site/about/guidelines.xhtml

19. I understand my occupation must not involve direct contact with young children (defined as those attending pre-school groups or nursery or aged under 2 years) or patient contact in a health or social care setting until I am shown not to be infected with *Salmonella* Paratyphi A		
**Section 5: Additional**:		
**20.** I understand I should not have a household contact who is immunocompromised or children under the age of 2 or older children who are attending pre-school or nursery		
21. **For those involved in the provision of health or social care to vulnerable groups only:** I agree to my employer being informed of my participation in the trial.	 N/A	
22. **Women only:** I understand the need to ensure that I or my partner use effective contraception one month prior to vaccination and continue to do so until I am shown not to be infected with *Salmonella* Paratyphi A. I also understand that if I use oral hormonal contraception there is a need to use barrier contraception from vaccination until I am shown to be cleared of infection.	 N/A	
23. I agree to take part in this study.		
24. I agree to be contacted about other ethically approved research studies for which I may be suitable. I understand that agreeing to be contacted does not oblige me to participate in any further studies.	Yes	No

Name of Participant *Date* *Signature*

Name of Person taking Consent *Date* *Signature*

**1 copy for participant; Original for participant CRF;*

For peer review only - http://bmjopen.bmj.com/site/about/guidelines.xhtml

Supplementary Material 4: Visit Structure

Figure 1: Visit Structure of Trial

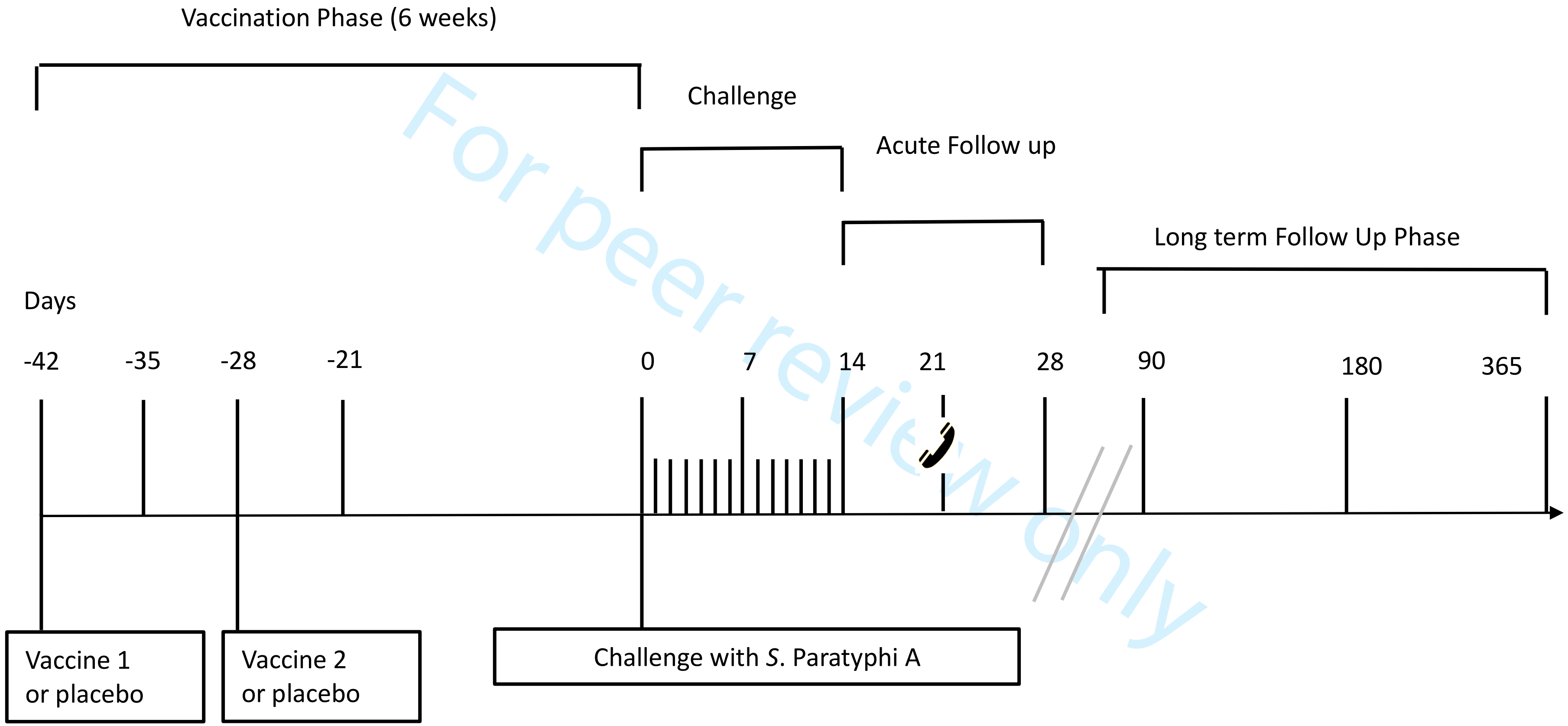

For peer review only

Figure 2: Structure of PD visits

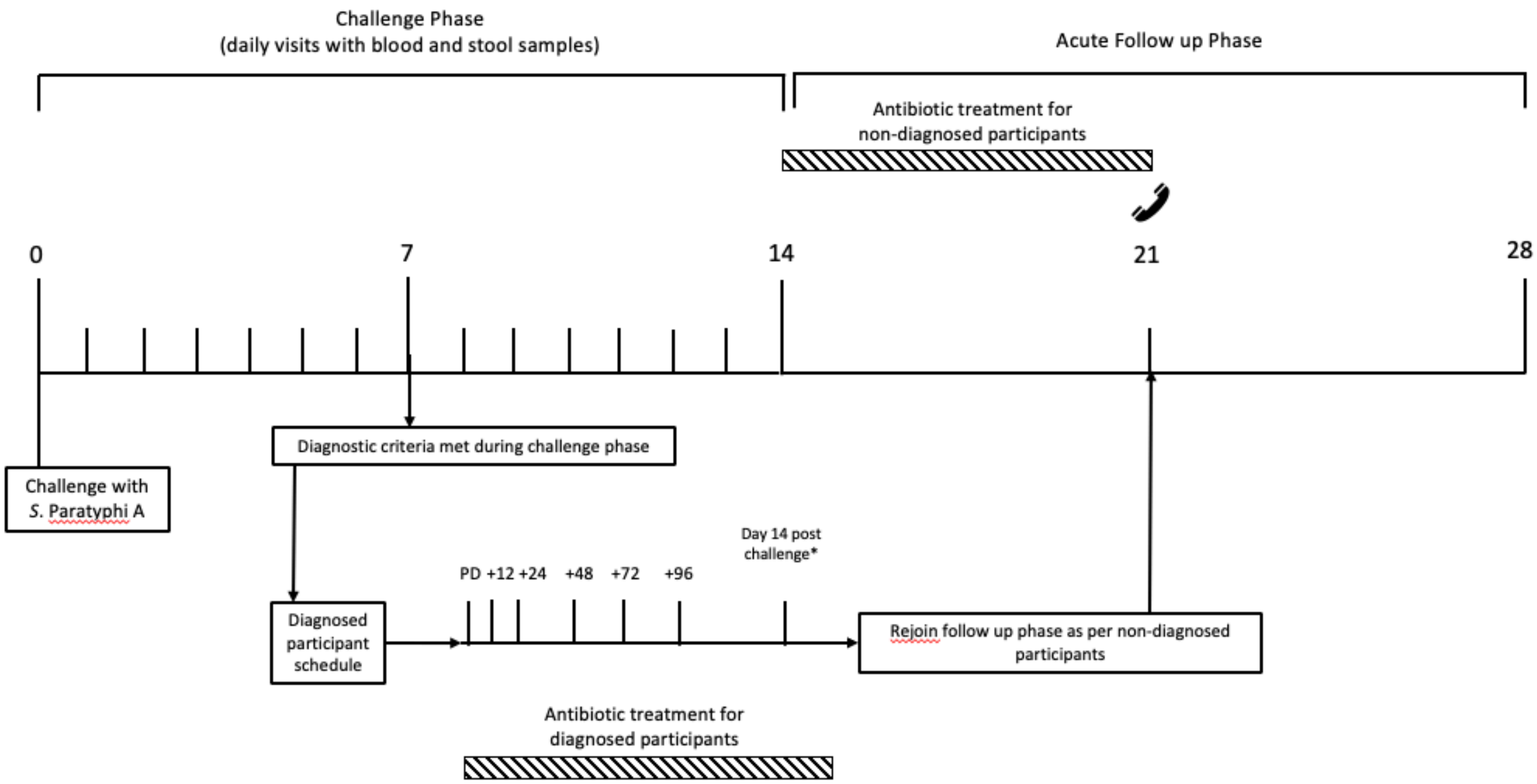

Supplementary Material 5: Vaccination e-diary

Sample of Daily Vaccination e-diary

Remember to measure your temperature **twice a day** following vaccination

	Day of First Vaccine	
Date		
AM/PM	AM	PM
Time		
Oral (mouth) temperature	°C	°C

**Symptom**	**Severity**				
Headache	0	1	2	3	4
Generally unwell	0	1	2	3	4
Loss of appetite	0	1	2	3	4
Abdominal/stomach pain	0	1	2	3	4
Nausea or vomiting	0	1	2	3	4
Diarrhoea	0	1	2	3	4

For peer review only

If you have not experienced a symptom please circle "0" on your diary page for the relevant day. If you have experienced a symptom please circle the number indicating the severity of the symptom using the scale.

Symptom	Severity			
	1	2	3	4
Headache	Present but no interference with activity	Some interference with activity	Significant; prevents daily activity	Emergency department visit or hospitalisation
Generally unwell	Present but no interference with activity	Some interference with activity	Significant; prevents dailyactivity	Emergency department visit or hospitalisation
Eating less than usual or loss of appetite	Eat less than normal for 1-2 meals	Miss 1-2 meals completely	Miss all meals	Emergency department visit or hospitalisation
Abdominal/ stomach pain	Present but no interference with activity	Some interference with activity	Significant; any use of codeine phosphate or prevents daily activity	Emergency department visit or hospitalisation
Nausea/vomiting	Present but no interference with activity or 1 – 2 episodes in 24 hours	Some interference with activity or more than 2 episodes in 24 hours	Significant; Prevents daily activity	Emergency department visit or hospitalisation
Diarrhoea	3-4 loose stools in 24 hrs	5-6 loose stools in 24 hrs	7 or more loose stools in 24 hrs	Emergency department visit or hospitalisation

For peer review only

If you feel feverish at any other time please check your temperature and record below:

Date								
Day (post vaccine)								
Time								
Oral (mouth) temperature	ºC	ºC	ºC	ºC	ºC	ºC	ºC	ºC

Date								
Day (post vaccine)								
Time								
Oral (mouth) temperature	ºC	ºC	ºC	ºC	ºC	ºC	ºC	ºC

For peer review only

Additional symptoms

perience other symptoms such as feeling hot or cold, sweating, shivering or constipat
able to cope with any of the symptoms you are experiencing, please contact the study
y.

rd any additional symptoms on the following tables. Please include any illness/injury ev
s not related to the vaccine. Please include the start date, start time, finish date, finish
going if not resolved by day 28), and severity of symptoms (see below scale).

e any symptoms using the following scale:
Mild – easily tolerated with no limitation on normal activity
Moderate – some limitation of daily activity
Severe – unable to perform normal daily activity
Emergency department or hospital visit required

For peer review only

**Start**<br>**te/time**	**Symptom details**	**Severity**<br>(please circle)	**Finish date/tin**<br>(state ongoing if con
/ __ / __<br>__ : __		1 2 3 4	__ / __ / __<br>__ : __
/ __ / __<br>__ : __		1 2 3 4	__ / __ / __<br>__ : __
/ __ / __<br>__ : __		1 2 3 4	__ / __ / __<br>__ : __
/ __ / __<br>__ : __		1 2 3 4	__ / __ / __<br>__ : __
/ __ / __<br>__ : __		1 2 3 4	__ / __ / __<br>__ : __
/ __ / __<br>__ : __		1 2 3 4	__ / __ / __<br>__ : __

v only

[illegible]	(24 hr clock)	[illegible]	[illegible]	[illegible]

For peer review only

For peer review only - http://bmjopen.bmj.com/site/about/guidelines.xhtml

For peer review only

Remember to measure your temperature **twice a day** following challenge

Day 0

	Day 0	
Date		
AM/PM	AM	PM
Time		
Oral (mouth) temperature	°C	°C

**Symptom**	**Severity**				
Headache	0	1	2	3	4
Generally unwell	0	1	2	3	4
Loss of appetite	0	1	2	3	4
Abdominal/stomach pain	0	1	2	3	4
Nausea or vomiting	0	1	2	3	4
Muscle pain	0	1	2	3	4
Joint pain	0	1	2	3	4
Cough	0	1	2	3	4
Diarrhoea	0	1	2	3	4
Constipation	0	1	2	3	4
Rash	YES/ NO delete as appropriate	Describe where, colour, size (mm):			

For peer review only

If you have not experienced a symptom please circle "0" on your diary page for the relevant day. If you have experienced a symptom please circle the number indicating the severity of the symptom using the scale.

Symptom	Severity			
	**1**	**2**	**3**	**4**
Headache	Present but no interference with activity	Some interference with activity	Significant; any use of codeine phosphate or prevents daily activity	Emergency department visit or hospitalisation
Generally unwell	Present but no interference with activity	Some interference with activity	Significant; prevents daily activity	Emergency department visit or hospitalisation
Eating less than usual or loss of appetite	Eat less than normal for 1-2 meals	Miss 1-2 meals completely	Miss all meals	Emergency department visit or hospitalisation
Abdominal/ stomach pain	Present but no interference with activity	Some interference with activity	Significant; any use of codeine phosphate or prevents daily activity	Emergency department visit or hospitalisation
Nausea/ vomiting	Present but no interference with activity or 1 – 2 episodes in 24 hours	Some interference with activity or more than 2 episodes in 24 hours	Significant; Prevents daily activity	Emergency department visit or hospitalisation

Symptom	Severity			
	**1**	**2**	**3**	**4**
Muscle pain	Present but no interference with activity	Some interference with activity	Significant; prevents daily activity	Emergency department visit or hospitalisation
Joint pain	Present but no interference with activity	Some interference with activity	Significant; prevents daily activity	Emergency department visit or hospitalisation

For peer review only

For peer review only - http://bmjopen.bmj.com/site/about/guidelines.xhtml

Cough	Present but no interference with activity	Some interference with activity	Significant; prevents daily activity	Emergency department visit or hospitalisation
Diarrhoea	3-4 loose stools in 24 hrs (loose stool is defined as ≥200ml volume)	5-6 loose stools in 24 hrs (loose stool is defined as ≥200ml volume)	7 or more loose stools in 24 hrs (loose stool is defined as ≥200ml volume)	Emergency department visit or hospitalisation
Constipation	Present but no interference with activity	Some interference with activity	Significant; prevents daily activity	Emergency department visit or hospitalisation

For peer review only

If you feel feverish at any other time please check your temperature and record below:

Date								
Day (post challenge)								
Time								
Oral (mouth) temperature	ºC	ºC	ºC	ºC	ºC	ºC	ºC	ºC

Date								
Day (post challenge)								
Time								
Oral (mouth) temperature	ºC	ºC	ºC	ºC	ºC	ºC	ºC	ºC

For peer review only

Additional Symptoms

You may experience other symptoms such as feeling hot or cold, sweating or shivering. If you feel unable to manage any of the symptoms you are experiencing, please contact the study team immediately.

Please record any additional symptoms on the following tables. Please include any illness/injury even if you think it is not related to the vaccine or paratyphoid challenge. Please include the start date, start time, finish date, finish time (or state ongoing if not resolved by day 28), and severity of symptoms (see below scale).

Please grade any symptoms using the following scale:

- 1 Mild – easily tolerated with no limitation on normal activity
- 2 Moderate – some limitation of daily activity
- 3 Severe – unable to perform normal daily activity
- 4 Emergency department or hospital visit required

For peer review only

**Start Date/time**	**Symptom details**	**Severity** (please circle)	**Finish date/time** (state ongoing if continuing)
__/__/__ __:__		1 2 3 4	__/__/__ __:__
__/__/__ __:__		1 2 3 4	__/__/__ __:__
__/__/__ __:__		1 2 3 4	__/__/__ __:__
__/__/__ __:__		1 2 3 4	__/__/__ __:__
__/__/__ __:__		1 2 3 4	__/__/__ __:__
__/__/__ __:__		1 2 3 4	__/__/__ __:__

For peer review only

Medications

Please record any medication taken in addition to any regular medication that you take. Please do not tal
agreed by the study team.

Date	Time (24 hr clock)	Medication	Dose	Reason

For peer review only

For peer review only

Supplementary Material 7: Sample testing during VASP Trial

Table 1: Sample collection - screening

Screening		
	Specimen	Container
FBC, ESR	Blood	EDTA vacutainer
Urea, Electrolytes & Creatinine	Blood	Heparin vacutainer
HIV, HBsAg, HCVA	Blood	Serum vacutainer
TTG and IgA	Blood	Serum vacutainer
HLA B27	Blood	EDTA vacutainer
Total blood volume		Maximum 17 mL
Blood glucose	Blood	NA
Urine dipstick	Urine	Standard urine pot
Urine Pregnancy test	Urine	Standard urine pot

Table 2: Sample collection – vaccination and post vaccination

Vaccine	Investigation	Pregnancy Test	Saliva	Stool sample	Blood culture	Full blood count	CRP, U+Es, LFTs	Blood for OVG laboratories	
	Sample tube	std urine pot	saliva coll-ection device	Stool Pot	Aerobic BACTEC bottle	EDTA Vacutainer	Heparin Vacutainer		
	**Volume blood (mL)**				10	1 → 3	2		**TOTAL***
	**Day**								
1	**D-42 (Va)**	x	x	x		2	3	74.5	79.5
	**D-35 (Vb)**			x	10	2	3	32.5	47.5
2	**D-28 (Vc)**	x	x	x	10	2	3	71	86
	**D-21 (Vd)**			x	10	2	3	32.5	47.5
								Total	260.5

Specific volumes and sample tubes for different assays for the OVG laboratories will be detailed in the Laboratory Analysis Plan and Clinical Study Plan.

For peer review

Table 3: Sample collection – challenge period

Challenge	Investigation	SARS-CoV-2 Test	Pregnancy Test	Saliva	Stool sample	Blood culture	Full blood count	CRP, U+Es, LFTs	Blood for OVG laboratories	
	Sample tube	NP swab	std urine pot	saliva coll-ection device	Stool Pot	Aerobic BACTEC bottle	EDTA Vacutainer	Heparin Vacutainer		
	**D-2**	x								
→	**D0**		x	x	x	10	2	3	81	96
	**D1**				x	10			20	30
	**D2**				x	10	2	3	up to 4	19
	**D3**				x	10			0	10
	**D4**				x	10	2	3	up to 4	19
	**D5**				x	10			0	10
	**D6**				x	10	2	3	0	15
	**D7**				x	10	2		58.5	70.5
	**D8**				x	10	2	3	0	15
	**D9**				x	10			0	10
	**D10**				x	10	2	3	0	15
	**D11**				x	10			0	10
	**D12**				x	10	2	3	0	15
	**D13**				x	10			0	10
	**D14**	x	x		x	10	2	3	58.5	73.5
									Total*	418

Specific volumes and sample tubes for different assays for the OVG laboratories will be detailed in the Laboratory Analysis Plan and Clinical Study Plan.

Table 4: Sample collection – paratyphoid diagnosis, follow up and totals

Investigation	SARS-CoV-2 Test	Pregnancy Test	Saliva	Stool sample	Blood culture	Full Blood count	CRP, U+Es, LFTs	Blood for OVG laboratories	
Sample tube	NP swab	std urine pot	saliva coll-ection device	Stool Pot	Aerobic BACTEC bottle	EDTA Vacutain er	Heparin Vacutain er		
**Day**									**TOTAL**
**PD**	x	x		x	10	2	3	12.5	27.5
**PD +12hrs**				x	10	2		50	62
**PD +24hrs**				x	10	2	3	**	15
**PD +48hrs**				x	10	2	3	0	15
**PD +72hrs**				x	10	2	3	0	15
**PD +96hrs**				x	10	2	3	50	65
**D14PD gp**				x		2	3	0	5
**D28**			x	x		2	3	58.5	63.5
								Total	268
	**Maximum total for first 3 months (excluding screening)**			**If Paratyphoid NOT diagnosed**				**Total**	**738**
	**Maximum total for first 3 months (excluding screening)**			**If Paratyphoid Diagnosed (maximum blood volume)**				**Total**	**937.5**
**D90**			x	x		2	3	58.5	63.5
**D180**			x	x		2	3	48.5	53.5
**D365**			x	x		2	3	58.5	63.5
	**Maximum total in 14 months (excluding screening)**			**If Paratyphoid NOT diagnosed**				**Total**	**922.5**
	**Maximum total in 14 months (excluding screening)**			**If Paratyphoid Diagnosed (maximum blood volume)**				**Total**	**1122**

Specific volumes and sample tubes for different assays for the OVG laboratories will be detailed in the Laboratory Analysis Plan and Clinical Study Plan.

Supplementary Material 8: Medications used in the VASP trial

Table 1: Concomitant medications used

Drug	Indication	Dose	Route	Frequency
Paracetamol	Fever and discomfort (**after antibiotic therapy started**)	500mg - 1 Gram	Oral	PRN, max QDS
Codeine	Pain including headache	15-60mg	Oral	PRN (max. 240mg/24 hours)
Senna	Constipation	2-4 tablets	Oral	PRN
Cyclizine	Nausea and/or vomiting	50mg	Oral	PRN (max. 150mg/24 hours)
Chlorpheniramine	Allergy	4mg	Oral	PRN TDS-QDS (max. 24mg/24 hours)
Oral rehydration salts	Dehydration, vomiting or diarrhoea	1-2 sachets	Oral	PRN
Sando-K	Hypokalaemia	2-4 tablets	Oral	PRN, up to TDS dependent on potassium deficit

Table 2: Antibiotics used

Drug	Dose	Route	Frequency	Duration
Ciprofloxacin	500mg	Oral	BD	7 days
Co-trimoxazole	160/800mg	Oral	BD	7 days
Azithromycin	1g stat followed by 500mg	Oral	OD	7 days
Amoxicillin	500mg	Oral	TDS	7 days

For peer review only

For peer review only

Supplementary Material 9: Trial Stopping Rules

Safety holding Rules

In the event of any of the following, vaccination of further individuals will be paused pending DSMC review:

- New scientific information is published to indicate that subjects in the trial are being exposed to undue risks as a result of administration of the IMP, or as a result of the trial procedures or follow-up schedule.
- Bacteraemia with *Salmonella* Paratyphi A of any participant during the vaccination period
- Serious concerns about the safety of the IMP arise as a result of one or more vaccine related SAE(s) occurring in the subjects enrolled
- If at least two subjects develop a 'severe' adverse event related to the study drug, as assessed by a clinician, independent of within or not within the same-organ-class.

The DSMC chair will then undertake a review of the data to decide whether a temporary halt is required as an urgent safety measure and what the scope of the halt will be. A full meeting of the DSMC can be called at the chair's discretion. If, following DSMC review, a halt is required, the Sponsor, REC and appropriate regulatory authorities will be notified within 3 days and a substantial amendment submitted within 15 days. If the DSMC decide that a halt is not required then the trial may continue.

Following a halt to the trial, if it is decided that the trial may re-start, then a request and substantial amendment will be made to the Sponsor, the REC and the MHRA in order to do so, otherwise the trial will be terminated.

Individual stopping Rules

Stopping rules for individual volunteers will apply (i.e., indications to withdraw individuals from further vaccinations). Study participants who present with at least one of the following stopping rules will trigger a clinical review as to whether the participant should be withdrawn from further vaccination in the study, would not undergo challenge and would be followed up only from a safety perspective if participants consent to this.

- Laboratory Aes: the participant develops a confirmed $\geq$ grade 3 laboratory AE considered possibly, probably or definitely related within 7 days after vaccination
- Solicited adverse events: the participant develops a $\geq$ grade 3 systemic solicited AE considered possibly, probably or definitely related within 2 days after vaccination (day of vaccination and one subsequent day) which is deemed severe by clinician assessment
- Unsolicited adverse events: the participant has a $\geq$ grade 3 adverse event, considered possibly, probably or definitely related to vaccination which is deemed severe by clinician assessment or has a SAE considered possibly, probably or definitely related to vaccination.

For peer review only

- The participant has an acute allergic reaction or anaphylactic shock following the administration of the vaccine investigational product.

For peer review only

Supplementary material 10: Adverse Event grading

Adverse event	Grade	Definition (in degrees Celsius)
Temperature	0	< 37.6
	1	37.6 – 38.0
	2	38.1 – 39.0
	3	> 39.0

Participant grading of severity (vaccine phase)

	0	1	2	3	4
**Nausea /vomiting**	No symptoms	Present but no interference with activity or 1 – 2 episodes in 24 hours	Some interference with activity or more than 2 episodes in 24 hours	Significant; prevents daily activity	Emergency department visit or hospitalisation
**Diarrhoea**	No symptoms	3-4 loose stools in 24 hrs	5-6 loose stools in 24 hrs	7 or more loose stools in 24 hrs	Emergency department visit or hospitalisation
**Eating less than usual or loss of appetite**	No symptoms	Eat less than normal for 1-2 meals	Miss 1-2 meals completely	Miss all meals	Emergency department or hospital visit required
**Generally unwell**	No symptoms	Present but no interference with activity	Some interference with activity	Significant; prevents daily activity	Emergency department visit or hospitalisation
**Abdominal / stomach pain**	No symptoms	Present but no interference with activity	Some interference with activity	Significant; prevents daily activity	Emergency department visit or hospitalisation
**Headache**	No symptoms	Present but no interference with activity	Some interference with activity	Significant; prevents daily activity	Emergency department visit or hospitalisation

For peer review only

Participant grading of severity (challenge phase)

	0	1	2	3	4
**Headache**	No symptoms	Present but no interference with activity	Some interference with activity	Significant; any use of codeine phosphate or prevents daily activity	Emergency department visit or hospitalisation
**Generally unwell**	No symptoms	Present but no interference with activity	Some interference with activity	Significant; prevents daily activity	Emergency department visit or hospitalisation
**Eating less than usual or loss of appetite**	No symptoms	Eat less than normal for 1-2 meals	Miss 1-2 meals completely	Miss all meals	Emergency department or hospital visit required
**Abdominal/ stomach pain**	No symptoms	Present but no interference with activity	Some interference with activity	Significant; any use of codeine phosphate or prevents daily activity	Emergency department visit or hospitalisation
**Nausea/vomiting**	No symptoms	Present but no interference with activity or 1 – 2 episodes in 24 hours	Some interference with activity or more than 2 episodes in 24 hours	Significant; prevents daily activity	Emergency department visit or hospitalisation
**Muscle pain**	No symptoms	Present but no interference with activity	Some interference with activity	Significant; any use of codeine phosphate or prevents daily activity	Emergency department visit or hospitalisation
**Joint pain**	No symptoms	Present but no interference with activity	Some interference with activity	Significant; any use of codeine phosphate or prevents daily activity	Emergency department visit or hospitalisation
**Cough**	No symptoms	Present but no interference with activity	Some interference with activity	Significant; any use of codeine phosphate or prevents daily activity	Emergency department visit or hospitalisation

For peer review only

**Diarrhoea**	No symptoms	3-4 loose stools in 24 hrs	5-6 loose stools in 24 hrs	7 or more loose stools in 24 hrs	Emergency department visit or hospitalisatio n
**Constipatio n**	No symptoms	Present but no interference with activity or 1 – 2 episodes in 24 hours	Some interference with activity or more than 2 episodes in 24 hours	Significant; prevents daily activity	Emergency department visit or hospitalisatio n
**Rash**	No symptoms	Yes/No			

For peer review only

Grading the severity of visit observed Adverse Events

Observation	Grade 1	Grade 2	Grade 3	Grade 4
Oral temperature (℃)	37.6 – 38.0	38.1 – 39.0	> 39.0	A&E visit or hospitalisation for hyperpyrexia
Tachycardia (beats/min)	101-115	116-130	>130	A&E visit or hospitalisation for arrhythmia
Bradycardia (beats/min)	50-54	45-49	<45	A&E visit or hospitalisation for arrhythmia
Systolic hyper-tension (mmHg)	141-150	151-155	>155	A&E visit or hospitalization for malignant hypertension
Diastolic hyper-tension (mmHg)	91-95	96-100	>100	A&E visit or hospitalization for malignant hypertension
Systolic hypo-tension (mmHg)	85-89	80-84	<80	A&E visit or hospitalization for hypotensive shock

For peer review only

Grading the severity of laboratory Adverse Events

Parameter	Grade 1	Grade 2	Grade 3	Grade 4*
**Haemoglobin: decrease from baseline value (g/l)**	10 - 15	16-20	21-50	>50
**White cell count: elevated (10^9/L)**	11–15	16–20	21–25	>25
**White cell count: depressed (10^9/L)**	2.5-3.5	1.5-2.4	1.0-1.4	<1.0
**Neutrophil count (10^9/L)**	1.5-2.0	1.0-1.4	0.5-0.9	<0.5
**Platelets (10^9/L)**	125-140	100-124	25-99	<25
**Sodium: hyponatraemia (mmol/L)**	132–134	130–131	125–129	<125
**Sodium: hypernatraemia (mmol/L)**	146	147	148–150	>150
**Potassium: hyperkalaemia (mmol/L)**	5.1–5.2	5.3–5.4	5.5–5.6	>5.6
**Potassium: hypokalaemia (mmol/L)**	3.3–3.4	3.1–3.2	3.0	<3.0
**Urea (mmol/L)**	8.2–8.9	9.0–11	>11	RRT
**Creatinine (µmol/L)**	132-150	151-176	177-221	>221 or RRT
**ALT and/or AST (IU/L)**	1.1–2.5 x ULN	>2.6–5.0 x ULN	5.1-10 x ULN	>10 x ULN
**Bilirubin, with increase in LFTs (µmol/L)**	1.1–1.25 x ULN	1.26–1.5 x ULN	1.51–1.75 x ULN	>1.75 x ULN
**Bilirubin, with normal LFTs (µmol/L)**	1.1–1.5 x ULN	1.6–2.0 x ULN	2.1–3.0 x ULN	>3.0 x ULN
**Alkaline phosphatase (IU/L)**	1.1–2.0 x ULN	2.1–3.0 x ULN	3.1–10 x ULN	>10 x ULN
**Albumin: hypoalbuminaemia (g/L)**	28–31	25–27	<25	Not applicable
**C-reactive protein**	>10-30	31-100	101-200	>200

Grade 4* Potentially life threatening

For peer review only - http://bmjopen.bmj.com/site/about/guidelines.xhtml

STANDARD PROTOCOL ITEMS: RECOMMENDATIONS FOR INTERVENTIONAL TRIALS

SPIRIT 2013 Checklist: Recommended items to address in a clinical trial protocol and related documents*

Section/item	Item No	Description	Page number in manuscript
**Administrative information**			
Title	1	Descriptive title identifying the study design, population, interventions, and, if applicable, trial acronym	1
Trial registration	2a	Trial identifier and registry name. If not yet registered, name of intended registry	2
	2b	All items from the World Health Organization Trial Registration Data Set	NA
Protocol version	3	Date and version identifier	4
Funding	4	Sources and types of financial, material, and other support	17
Roles and responsibilities	5a	Names, affiliations, and roles of protocol contributors	17
	5b	Name and contact information for the trial sponsor	17
	5c	Role of study sponsor and funders, if any, in study design; collection, management, analysis, and interpretation of data; writing of the report; and the decision to submit the report for publication, including whether they will have ultimate authority over any of these activities	17
	5d	Composition, roles, and responsibilities of the coordinating centre, steering committee, endpoint adjudication committee, data management team, and other individuals or groups overseeing the trial, if applicable (see Item 21a for data monitoring committee)	17
**Introduction**			
Background and rationale	6a	Description of research question and justification for undertaking the trial, including summary of relevant studies (published and unpublished) examining benefits and harms for each intervention	3-4
	6b	Explanation for choice of comparators	6

For peer review only

Objectives	7	Specific objectives or hypotheses	4-5<br>Table 1
Trial design	8	Description of trial design including type of trial (eg, parallel group, crossover, factorial, single group), allocation ratio, and framework (eg, superiority, equivalence, noninferiority, exploratory)	6
**Methods: Participants, interventions, and outcomes**			
Study setting	9	Description of study settings (eg, community clinic, academic hospital) and list of countries where data will be collected. Reference to where list of study sites can be obtained	6
Eligibility criteria	10	Inclusion and exclusion criteria for participants. If applicable, eligibility criteria for study centres and individuals who will perform the interventions (eg, surgeons, psychotherapists)	Boxes 1 & 2, supplementary material 1 & 2
Interventions	11a	Interventions for each group with sufficient detail to allow replication, including how and when they will be administered	10-12
	11b	Criteria for discontinuing or modifying allocated interventions for a given trial participant (eg, drug dose change in response to harms, participant request, or improving/worsening disease)	14, supplementary material 8
	11c	Strategies to improve adherence to intervention protocols, and any procedures for monitoring adherence (eg, drug tablet return, laboratory tests)	15-16
	11d	Relevant concomitant care and interventions that are permitted or prohibited during the trial	12-13
Outcomes	12	Primary, secondary, and other outcomes, including the specific measurement variable (eg, systolic blood pressure), analysis metric (eg, change from baseline, final value, time to event), method of aggregation (eg, median, proportion), and time point for each outcome. Explanation of the clinical relevance of chosen efficacy and harm outcomes is strongly recommended	Table 1
Participant timeline	13	Time schedule of enrolment, interventions (including any run-ins and washouts), assessments, and visits for participants. A schematic diagram is highly recommended (see Figure)	Figure 1

For peer review only - http://bmjopen.bmj.com/site/about/guidelines.xhtml

Sample size	14	Estimated number of participants needed to achieve study objectives and how it was determined, including clinical and statistical assumptions supporting any sample size calculations	12
Recruitment	15	Strategies for achieving adequate participant enrolment to reach target sample size	6

Methods: Assignment of interventions (for controlled trials)

Allocation:

Sequence generation	16a	Method of generating the allocation sequence (eg, computer-generated random numbers), and list of any factors for stratification. To reduce predictability of a random sequence, details of any planned restriction (eg, blocking) should be provided in a separate document that is unavailable to those who enrol participants or assign interventions	9
Allocation concealment mechanism	16b	Mechanism of implementing the allocation sequence (eg, central telephone; sequentially numbered, opaque, sealed envelopes), describing any steps to conceal the sequence until interventions are assigned	9
Implementation	16c	Who will generate the allocation sequence, who will enrol participants, and who will assign participants to interventions	9
Blinding (masking)	17a	Who will be blinded after assignment to interventions (eg, trial participants, care providers, outcome assessors, data analysts), and how	9
	17b	If blinded, circumstances under which unblinding is permissible, and procedure for revealing a participant's allocated intervention during the trial	9

Methods: Data collection, management, and analysis

Data collection methods	18a	Plans for assessment and collection of outcome, baseline, and other trial data, including any related processes to promote data quality (eg, duplicate measurements, training of assessors) and a description of study instruments (eg, questionnaires, laboratory tests) along with their reliability and validity, if known. Reference to where data collection forms can be found, if not in the protocol	11, Supplementary materials 5-7

	18b	Plans to promote participant retention and complete follow-up, including list of any outcome data to be collected for participants who discontinue or deviate from intervention protocols	13
Data management	19	Plans for data entry, coding, security, and storage, including any related processes to promote data quality (eg, double data entry; range checks for data values). Reference to where details of data management procedures can be found, if not in the protocol	11,13
Statistical methods	20a	Statistical methods for analysing primary and secondary outcomes. Reference to where other details of the statistical analysis plan can be found, if not in the protocol	12-13
	20b	Methods for any additional analyses (eg, subgroup and adjusted analyses)	12-13
	20c	Definition of analysis population relating to protocol non-adherence (eg, as randomised analysis), and any statistical methods to handle missing data (eg, multiple imputation)	
**Methods: Monitoring**			
Data monitoring	21a	Composition of data monitoring committee (DMC); summary of its role and reporting structure; statement of whether it is independent from the sponsor and competing interests; and reference to where further details about its charter can be found, if not in the protocol. Alternatively, an explanation of why a DMC is not needed	14
	21b	Description of any interim analyses and stopping guidelines, including who will have access to these interim results and make the final decision to terminate the trial	Supplementary table 8
Harms	22	Plans for collecting, assessing, reporting, and managing solicited and spontaneously reported adverse events and other unintended effects of trial interventions or trial conduct	15-16, Table 3
Auditing	23	Frequency and procedures for auditing trial conduct, if any, and whether the process will be independent from investigators and the sponsor	11
**Ethics and dissemination**			
Research ethics approval	24	Plans for seeking research ethics committee/institutional review board (REC/IRB) approval	13

For peer review only - http://bmjopen.bmj.com/site/about/guidelines.xhtml

Protocol amendments	25	Plans for communicating important protocol modifications (eg, changes to eligibility criteria, outcomes, analyses) to relevant parties (eg, investigators, REC/IRBs, trial participants, trial registries, journals, regulators)	14
Consent or assent	26a	Who will obtain informed consent or assent from potential trial participants or authorised surrogates, and how (see Item 32)	6
	26b	Additional consent provisions for collection and use of participant data and biological specimens in ancillary studies, if applicable	11
Confidentiality	27	How personal information about potential and enrolled participants will be collected, shared, and maintained in order to protect confidentiality before, during, and after the trial	11
Declaration of interests	28	Financial and other competing interests for principal investigators for the overall trial and each study site	18
Access to data	29	Statement of who will have access to the final trial dataset, and disclosure of contractual agreements that limit such access for investigators	11
Ancillary and post-trial care	30	Provisions, if any, for ancillary and post-trial care, and for compensation to those who suffer harm from trial participation	14
Dissemination policy	31a	Plans for investigators and sponsor to communicate trial results to participants, healthcare professionals, the public, and other relevant groups (eg, via publication, reporting in results databases, or other data sharing arrangements), including any publication restrictions	16
	31b	Authorship eligibility guidelines and any intended use of professional writers	16
	31c	Plans, if any, for granting public access to the full protocol, participant-level dataset, and statistical code	NA
**Appendices**			
Informed consent materials	32	Model consent form and other related documentation given to participants and authorised surrogates	Supplementary material 2

For peer review only - http://bmjopen.bmj.com/site/about/guidelines.xhtml

Biological specimens	33	Plans for collection, laboratory evaluation, and storage of biological specimens for genetic or molecular analysis in the current trial and for future use in ancillary studies, if applicable	11

*It is strongly recommended that this checklist be read in conjunction with the SPIRIT 2013 Explanation & Elaboration for important clarification on the items. Amendments to the protocol should be tracked and dated. The SPIRIT checklist is copyrighted by the SPIRIT Group under the Creative Commons "Attribution-NonCommercial-NoDerivs 3.0 Unported" license.

For peer review only
